# A common gene signature of the right ventricle in failing rat and human hearts

**DOI:** 10.1038/s44161-024-00485-1

**Published:** 2024-07-05

**Authors:** Liane Jurida, Sebastian Werner, Fabienne Knapp, Bernd Niemann, Ling Li, Dimitri Grün, Stefanie Wirth, Axel Weber, Knut Beuerlein, Christoph Liebetrau, Christoph B. Wiedenroth, Stefan Guth, Baktybek Kojonazarov, Leili Jafari, Norbert Weissmann, Stefan Günther, Thomas Braun, Marek Bartkuhn, Ralph T. Schermuly, Peter Dorfmüller, Xiaoke Yin, Manuel Mayr, M. Lienhard Schmitz, Laureen Czech, Klaus-Dieter Schlüter, Rainer Schulz, Susanne Rohrbach, Michael Kracht

**Affiliations:** 1 Rudolf Buchheim Institute of Pharmacology, Justus Liebig University, Giessen, Germany; 2 Department of Physiology, Justus Liebig University, Giessen, Germany; 3 Department of Cardiac and Vascular Surgery, Justus Liebig University, Giessen, Germany; 4 Department of Cardiology and Angiology, Justus Liebig University, Giessen, Germany; 5 Department of Cardiology, Kerckhoff Heart and Lung Center, Bad Nauheim, Germany; 6 Department of Thoracic Surgery, Kerckhoff Heart and Lung Center, Bad Nauheim, Germany; 7 Institute for Lung Health, Justus Liebig University, Giessen, Germany; 8 Medical Clinic II, Justus Liebig University, Giessen, Germany; 9 Cardio-Pulmonary Institute, Giessen, Germany; 10 Universities of Giessen and Marburg Lung Center (UGMLC), Giessen, Germany; 11 German Center for Lung Research (DZL), Giessen, Germany; 12 Department of Lung Development and Remodeling, Max Planck Institute for Heart and Lung Research, Bad Nauheim, Germany; 13 Department of Cardiac Development and Remodeling, Max Planck Institute for Heart and Lung Research, Bad Nauheim, Germany; 14 Biomedical Informatics and Systems Medicine, Science Unit for Basic and Clinical Medicine, Institute for Lung Health, Justus Liebig University Giessen, Giessen, Germany; 15 Department of Internal Medicine, Justus Liebig University Giessen, Giessen, Germany; 16 Institute of Pathology, Justus Liebig University Giessen, Giessen, Germany; 17 National Heart and Lung Institute, Faculty of Medicine,Imperial College London, London, UK; 18 Institute of Biochemistry, Justus Liebig University, Giessen, Germany

**Keywords:** Heart failure, Predictive markers

## Abstract

The molecular mechanisms of progressive right heart failure are incompletely understood. In this study, we systematically examined transcriptomic changes occurring over months in isolated cardiomyocytes or whole heart tissues from failing right and left ventricles in rat models of pulmonary artery banding (PAB) or aortic banding (AOB). Detailed bioinformatics analyses resulted in the identification of gene signature, protein and transcription factor networks specific to ventricles and compensated or decompensated disease states. Proteomic and RNA-FISH analyses confirmed PAB-mediated regulation of key genes and revealed spatially heterogeneous mRNA expression in the heart. Intersection of rat PAB-specific gene sets with transcriptome datasets from human patients with chronic thromboembolic pulmonary hypertension (CTEPH) led to the identification of more than 50 genes whose expression levels correlated with the severity of right heart disease, including multiple matrix-regulating and secreted factors. These data define a conserved, differentially regulated genetic network associated with right heart failure in rats and humans.

## Abstract (web summary)

Using bulk heart transcriptomics of rat models of right and left ventricle failure, Jurida et al. examined transcriptional changes in cardiomyocytes during the progression of heart failure and the overlap with transcriptomics from humans with chronic thromboembolic pulmonary hypertension (CTEPH), identifying more than 50 genes whose expression levels correlate with the severity of right heart disease.

## Main

Heart failure (HF) is a common clinical syndrome characterized by the progressive inability of the heart to pump sufficient blood volume to the body’s organs^1^. HF is a major global health problem, with a prevalence of approximately 1–2% in developed countries and increasing mortality rates^2,3^. Compared to left heart failure (LHF), available epidemiological evidence on right heart failure (RHF) is scarce^4^.

RHF occurs when the right ventricle (RV) fails to pump blood effectively through the lungs, leading to backward failure causing congestion in the systemic circulation^5^. RHF can be caused by pulmonary hypertension (PH), chronic pulmonary disease, ischemia, tricuspid valve disease or left-sided heart failure^6^.

Compared to the left ventricle (LV), the RV has a thinner myocardium, a lower contractile force and a more compliant wall. These properties reflect the lower pressure load placed on it by the pulmonary circulation^7^. The RV has a complex three-dimensional anatomy and a very distinct contraction pattern^8^. Long perceived as the ‘low pressure bystander’ of the LV that largely consists of the same cardiomyocytes, the RV is, in fact, derived from a different set of precursor cells during embryonic development^9^.

These differences offer possible explanations for the observation that treatments developed for LHF are often not effective in RHF^9,10^. To date, mechanisms of RHF, and its specific different functional and molecular responses to pressure versus volume overload, are still incompletely understood^9^.

Advances in surgical, medical and device therapies have demonstrated the capacity of the heart to reverse, at least in part, the failing phenotype. However, a more careful characterization of the molecular changes associated with this effect is necessary to define true myocardial recovery, in which a failing heart regains both normal function and molecular makeup^11^.

Transcriptomic studies have contributed fundamentally to knowledge on myocardial remodeling during HF, but key genes reported have been often inconsistent between studies^12–14^. To resolve some of these issues, a recent meta-analysis curated and uniformly processed 16 transcriptomic studies comprising 263 healthy and 653 failing human hearts, collected during heart transplantation, LV assist device implantation or surgical ventricular restoration, to derive a consensus signature of LHF^15^.

Similar resources and datasets are not available for RHF. In the present study, we performed a systematic investigation of rat models of chronic RHF (pulmonary artery banding (PAB)) or LHF (aortic banding (AOB)) to uncover the transcriptomic and proteomic changes that occur over months in the failing RV compared to the failing LV. Deep bioinformatics analyses, including comparisons of rat PAB-specific gene sets with transcriptomic data from patients with chronic thromboembolic pulmonary hypertension (CTEPH) before and after pulmonary endarterectomy (pre/post PEA) resulted in the identification of more than 50 genes whose expression levels correlated with the severity of right heart disease in humans. Together, these data define a genetic network representing a first version of a core gene signature of the failing RV that appears to coordinate progressive RHF.

## Results

### Transcriptomic changes in cardiomyocytes from rat HF models

To identify chamber-specific molecular pathways in RVF compared to LVF, we established rat models of chronic, progressive heart disease using PAB, to induce compensatory RV hypertrophy and failure (RVH and RVF), or, AOB, to induce compensatory LV hypertrophy and failure (LVH and LVF)^16^.

**Fig. 1:**
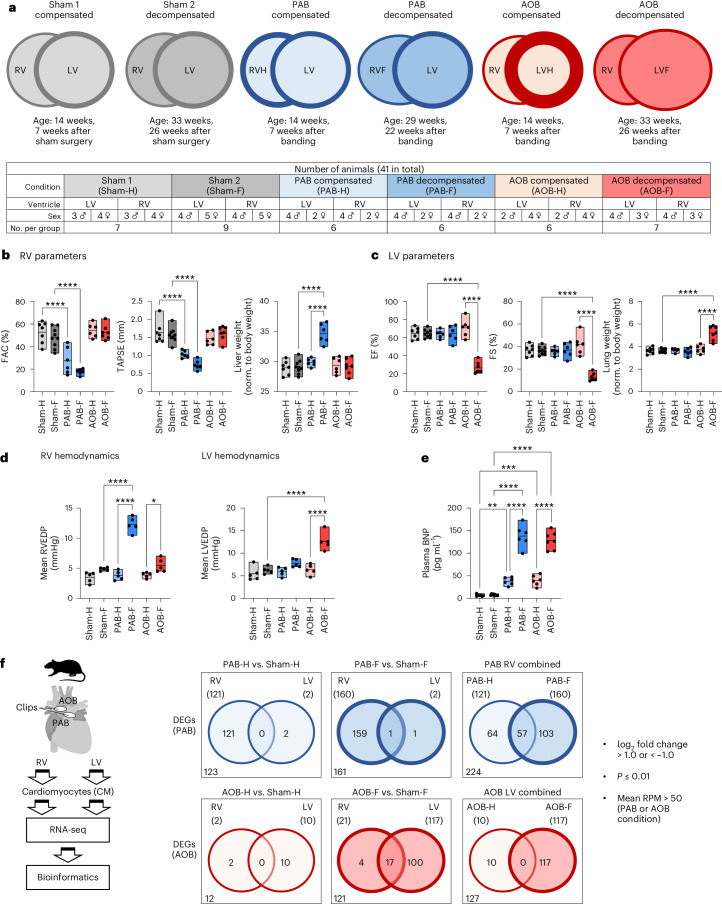
Identification of differentially regulated gene sets in cardiomyocytes from rat models of progressive RHF and LHF. **a**, Overview of animal study design. PAB or AOB was performed in rats to slowly induce RHF or LHF. Sham-operated animals served as controls. **b**, Echocardiographic validation of RV function and liver weight of rat models used for CM isolation. **c**, Echocardiographic validation of LV function and lung weight of rat models used for cardiomyocyte isolation. **d**, Sham, PAB and AOB surgery was performed in an independent animal cohort. End-diastolic pressure (EDP) was determined by right heart (left graphs) or left heart (right graphs) catheterization in sham controls and after banding at week 14 (compensated stage, PAB-H, AOB-H), at week 29 (PAB decompensated stage, PAB-F) or week 33 (AOB decompensated stage, AOB-F). *n* = 5 animals per group. **e**, Plasma BNP levels of compensated and decompensated states in response to PAB or AOB in the rat models from **b** and **c**. **f**, Total RNA from RV and LV cardiomyocytes was isolated, and 17,341 rat genes were analyzed by RNA-seq. Based on two-fold changes, mean read counts in disease conditions of more than 50 and *P* ≤ 0.01, DEGs were identified in each condition by comparing the PAB or AOB groups to their corresponding sham groups. Venn diagrams indicate overlapping and distinct groups of DEGs for PAB (224 genes) and AOB (127 genes) that were further systematically analyzed in this study. **b**,**c**,**e**,**f**, *n* = 6–9 animals per group as shown in **a**. Box plots in **b**–**e** show data points from all individual animals with means and minimum/maximum values. Asterisks indicate significant changes according to one-way ANOVA (*P ≤ 0.05, **P ≤ 0.01, ***P ≤ 0.001, ****P ≤ 0.0001). RPM, reads per million. Source data

**Extended Data Fig. 1:**
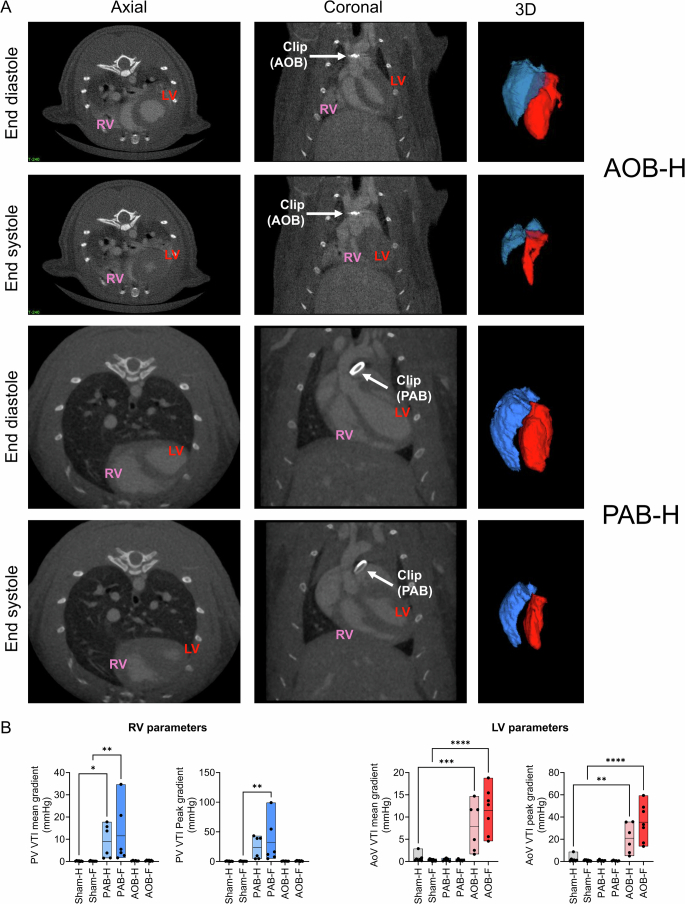
Functional validation of PAB or AOB rat models. (**a**) µCT images with contrast agent visualizing the position of the clip upon AOB or PAB of rats in the compensated states (AOB-H, PAB-H). (**b**) Further echocardiographic validation of compensated and decompensated states in response to banding in the rat models of RHF or LHF as assessed by stenosis parameters. PV VTI, pulmonary valve velocity time integral mean or peak gradient and AoV VTI, aortic valve velocity time integral mean or peak gradient. Box plots show data points from all individual animals with means and min / max values. Asterisks indicate significant changes according to one-way ANOVA (*p ≤ 0.05, **p ≤ 0.01, ***p ≤ 0.001, ****p ≤ 0.0001). n= 6–9 animals per group as shown in Fig. 1a. Source data

Initially, non-constricting clips placed around the pulmonary artery (for PAB) or the aorta (for AOB) of 7-week-old weanling rats resulted in subsequent vasoconstriction and compensatory heart hypertrophy at week 14 (PAB comp. or PAB-H, AOB comp. or AOB-H) as exemplified by micro-computed tomography (microCT) (Extended Data Fig. 1a). Decompensated HF developed at week 29 (PAB decomp. or PAB-F) or week 33 (AOB decomp. or AOB-F). Controls for disease-specific and age-specific changes included sham-operated animals euthanized at week 14 (Sham 1 or Sham-H) or at week 33 (Sham 2 or Sham-F) (Fig. 1a).

**Extended Data Fig. 2:**
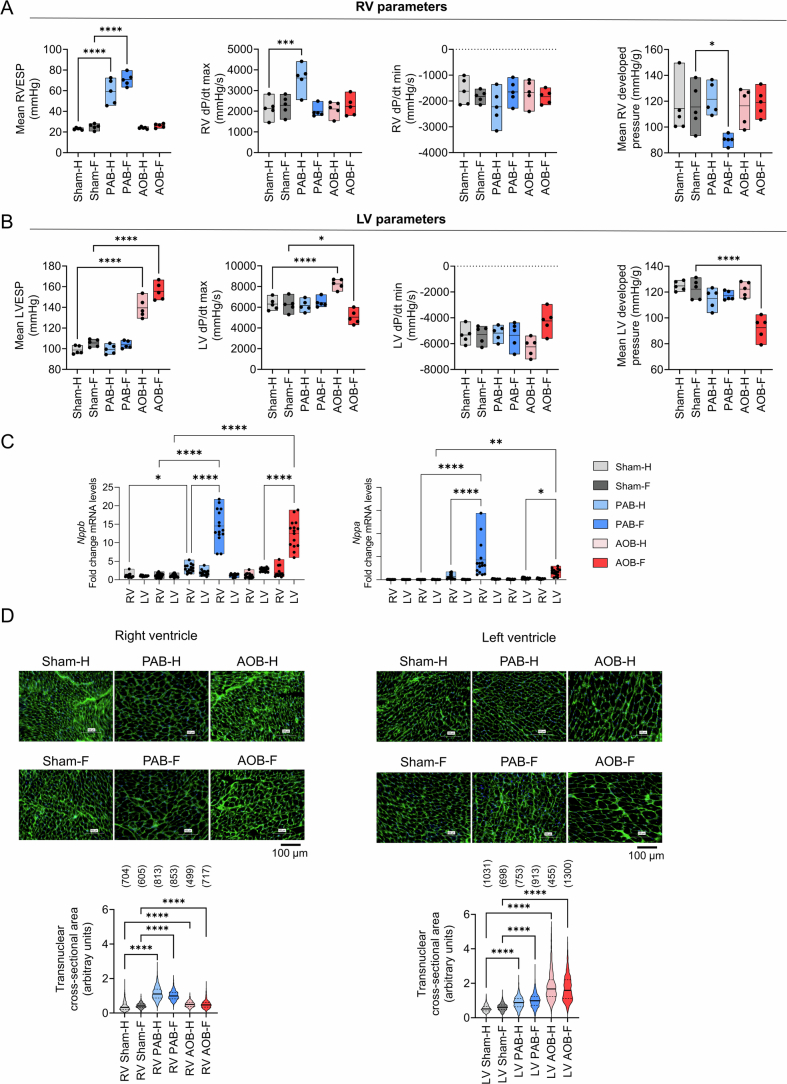
Hemodynamic and further functional validation of PAB and AOB models in other cohorts. (**a, b**) Sham, PAB and AOB surgery was performed in an independent animal cohort. At week 14 (PAB-H / AOB-H, compensated stage), week 29 (PAB-F, decompensated stage) or week 33 (AOB-F, decompensated stage) hemodynamic parameters were obtained by left or right heart catheterization. In (a), right ventricular (RV) endsystolic pressure (RVESP), maximum rate of RV pressure increase (+dP/dt max) and maximum rate of RV pressure decrease (−dP/dt max) and mean RV developed pressure were assessed. In (b), left ventricular (LV) endsystolic pressure (LVESP), maximum rate of LV pressure increase (+dP/dt max), maximum rate of LV pressure decrease (−dP/dt max) and mean LV developed pressure were determined. Box plots show data points with means and min / max values from individual animals. Asterisks indicate significant changes according to one-way ANOVA (*p ≤ 0.05, **p ≤ 0.01, ***p ≤ 0.001, ****p ≤ 0.0001). n=5 animals per group. (**c**) Total RNA isolated from individual ventricles of animals subjected to PAB or AOB according to the scheme shown in Fig. 1a was used to determine the expression of *Nppa* and *Nppb* by RT-qPCR. Relative mRNA levels compared to the mean of the Sham conditions for each ventricle are shown. Box plots show data points from five individual animals with means and min / max values, including three technical replicates, per group. Asterisks indicate significant changes according to one-way ANOVA (*p ≤ 0.05, **p ≤ 0.01, ***p ≤ 0.001, ****p ≤ 0.0001). (**d**) Heart sections from rats treated as in Fig. 1a were stained with FITC-coupled wheat germ agglutinin (green). Nuclei were stained with DAPI (blue). Upper parts: Representative micrographs are shown for each condition. Scale bars indicate 100 µm. Lower parts: Cardiomyocyte cross sectional areas were quantified in these tissue sections with ImageJ. Violin plots show medians, whiskers show 1^st^ and 3^rd^ quartiles. Total cell numbers from five animals per group are indicated in brackets. Significant changes were identified by one-way ANOVA; asterisks indicate p values (*p ≤ 0.05, **p ≤ 0.01, ***p ≤ 0.001, ****p ≤ 0.0001). Source data

Disease progression was monitored by echocardiography and clinical parameters (Fig. 1b,c,e and Extended Data Fig. 1b). Catheter-based hemodynamic measurements were obtained from a similar cohort of animals (Fig. 1d and Extended Data Fig. 2a,b). Fractional area change (FAC) and tricuspid annular plane systolic excursion (TAPSE) confirmed the onset of compromised RV function. RHF in PAB decomp. groups was evident from increased liver weight and right ventricle end-diastolic pressure (RVEDP) and decrease in the maximal rate of rise of right ventricular pressure (RV dp/dt maximum values) (Fig. 1b,d and Extended Data Figs. 1b and 2a).

Likewise, LV parameters (ejection fraction (EF) and fractional shortening (FS)) demonstrated altered LV function, whereas, only in the AOB decomp. group, increased lung weight, increased LV end-diastolic pressure (LVEDP) and decreased LV dp/dt maximum values clearly demarcated the transition to LHF (Fig. 1c,d and Extended Data Figs. 1b and 2b).

Progressive increases in serum levels of brain (or B-type) natriuretic peptide (BNP) (Fig. 1e), elevated mRNA expression levels of established markers *Nppb* or *Nppa* (Extended Data Fig. 2c) and increased cardiomyocyte hypertrophy (Extended Data Fig. 2d) confirmed that both PAB and AOB resulted in terminal HF that proceeded through an intermediate compensatory state.

**Extended Data Fig. 3:**
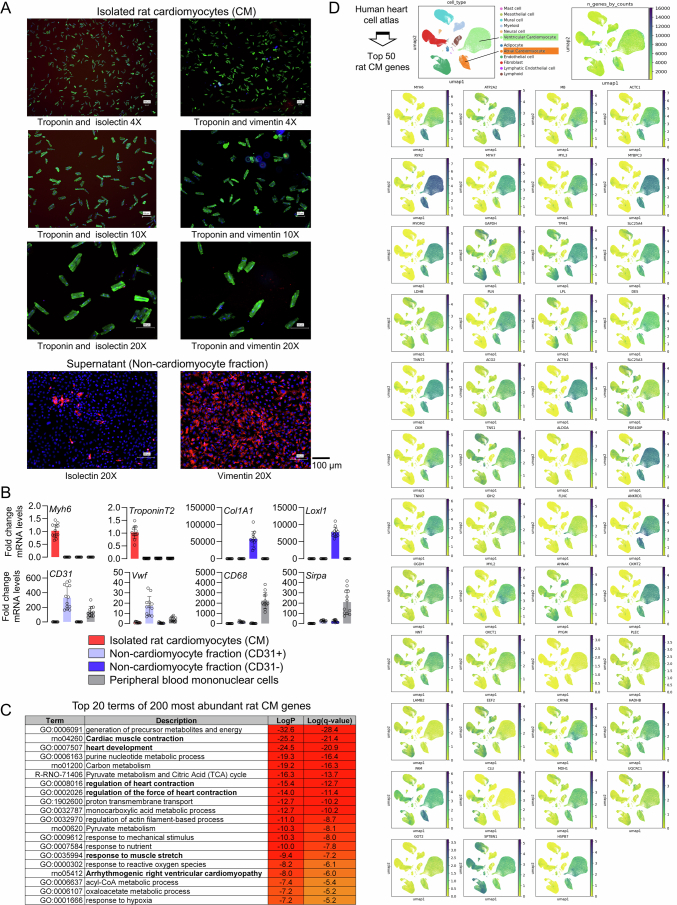
Purity of cardiomyocyte preparations. (**a**) Upper panel: Freshly isolated rat cardiomyocytes were seeded on laminin-treated glass bottom dishes and the expression of marker proteins for cardiomyocytes (cardiac troponin T, cTnT, green), endothelial cells (isolectin B4, red) or fibroblasts (vimentin, red) was analyzed by indirect immunofluorescence microscopy. Nuclei were stained with DAPI. Representative micrographs at 4x, 10x and 20x magnification are shown. Lower panel: The same analysis was performed with cells contained in the supernatant (non-cardiomyocyte fraction). Data show a representative cell preparation from a single animal. Scale bars indicate 100 µm. The analysis was repeated four times independently with similar results. (**b**) Total RNA isolated from cardiomyocytes (CM), CD31+ and CD31- cells isolated form the non-cardiomyocyte fraction (the supernatant) and CD14*+* peripheral mononuclear cells were analyzed by RT-qPCR for the expression of marker genes for cardiomyocytes (*Myh6*, *Troponin T2*), fibroblasts (*Col1a1*, *Loxl1*), endothelial cells (*CD31*, *Vwf*) and immune cells (*CD68*, *Sirpa)*. Graphs show individual values (including technical duplicates) and means ± s.d. relative to the mean expression levels of CM. n= 12 biologically independent samples from 4 independent experiments. (**c**) The top 200 most abundantly expressed rat CM genes were selected and analysed for overrepresented pathway terms using Metascape. The heatmap shows the top 20 most enriched functional terms along with p and q values. Bold font highlights terms related to the heart. (**d**) The top 50 most abundantly expressed rat CM genes from Sham-H and Sham-F conditions were plotted on single cell / single nucleus RNAseq data from the human heart cell atlas, version 2 (https://www.heartcellatlas.org/index.html)^17,23^. Uniform manifold approximation and projection (UMAP) maps show expression of 47 matched genes, coloured by expression level, in 704,296 individual cells representing 12 cardiac cell types. Source data

**Extended Data Fig. 4:**
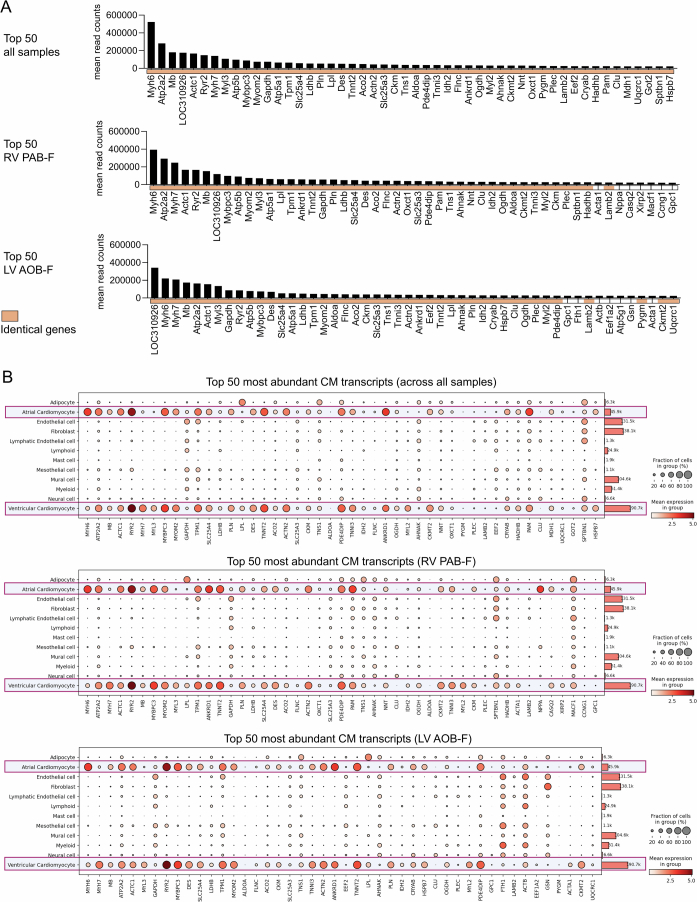
Purity of cardiomyocyte preparations in sham, PAB and AOB conditions. (**a**) Bar graphs showing normalized mean read counts from the top 50 most abundantly expressed rat CM genes across all samples and from the RV or LV from PAB-F or AOB-F conditions, respectively. Genes are ordered according to transcript abundance. Identical genes between groups are colored in beige. (**b**) The groups of genes from (A) were projected on single cell RNAseq data from the human heart cell atlas. Dot plots illustrate per group, the fraction of cells expressing a gene (dot size) and the mean expression of the gene in those cells (color scale). Numbers in side bars chart absolute numbers of cells assigned to each cell type category. Purple frames highlight atrial and ventricular cardiomyocytes. Source data

Total RNA sequencing (RNA-seq) was performed from cardiomyocytes isolated from LVs and RVs of all conditions, resulting in 82 datasets (Fig. 1f). Immunofluorescence and RT–qPCR analysis of markers for adult ventricular cardiomyocytes (troponin and *Myh6*), fibroblasts (vimentin, *Col1a1* and *Loxl1*), endothelial cells (isolectin, *CD31* and *Vwf*) or peripheral blood mononuclear cells (PBMCs) (*CD68* and *Sirpa*) indicated the quantitative removal of other heart cell types (Extended Data Fig. 3a,b). This was corroborated by a strong enrichment for heart-associated, multiple (energy) metabolism-related pathway terms among the top 200 most strongly expressed genes in cardiomyocyte and single-cell RNA-seq (scRNA-seq)/single-nucleus RNA-seq (snRNA-seq) data from the human heart cell atlas showing that the 50 most strongly expressed rat cardiomyocyte genes were largely confined to ventricular or atrial cardiomyocytes (Extended Data Figs. 3c,d and 4a,b)^17^.

**Extended Data Fig. 5:**
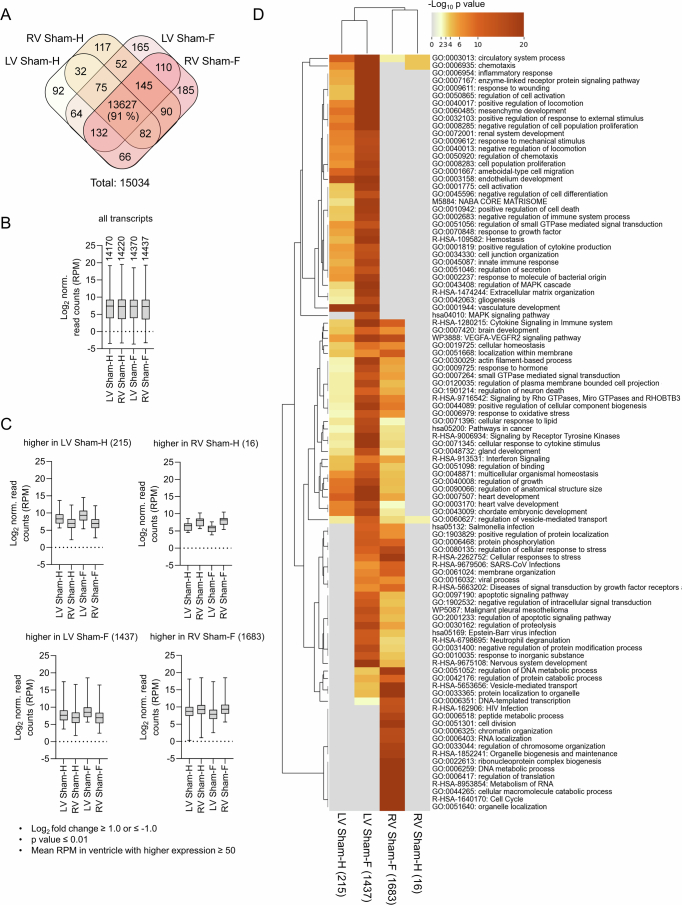
Common and ventricle-specific sets of genes in healthy rat hearts at sham 1 and sham 2 conditions. (**a**) Venn diagram showing the number of overlapping and distinct sets of genes in left (LV) or right ventricle (RV) of young (Sham-H) and older (Sham-F) rats based on normalized mean read counts (reads per million, RPM). (**b**) Graph showing the distribution of mRNA expression levels of all assessed genes. Boxes show 1^st^ to 3^rd^ quartile, whiskers show minimum to maximum of all values. n= 7 Sham-H animals and n=9 Sham-F animals as shown in Fig. 1a. (**c**) Summary of all genes with differential expression between LV or RV based on a Log_2_ fold change of ≥ 1.0 or ≤ −1, a p value ≤ 0.01 and mean RPM ≥ 50 in the ventricle with higher expression. Boxes show 1^st^ to 3^rd^ quartile, whiskers show minimum to maximum of all values. (**d**) Clustered heat map showing the top 100 overrepresented pathway terms for all genes with differential expression between LV or RV. Source data

In non-diseased conditions, 13,627 (91%) expressed genes overlapped, defining the common gene sets of RV and LV cardiomyocytes (Extended Data Fig. 5a,b). In total, 231 differentially expressed genes (DEGs) in the Sham-H and 3,120 DEGs in the Sham-F conditions, belonging to a plethora of biological processes, were found in the RV and LV of growing-up animals (Extended Data Fig. 5c,d). These results define the basal juvenile and adult chamber-dependent and age-dependent transcriptomes of healthy rat cardiomyocytes.

In decompensated HF conditions, by applying stringent filtering criteria (mean read counts in disease >50, two-fold changes and *P* ≤ 0.01), we found 160 DEGs in the RV upon PAB and 117 DEGs in the LV upon AOB, with few genes changing in the corresponding other ventricle (Fig. 1f, middle Venn diagrams).

In compensated conditions, 121 genes were specifically deregulated in the RV (but not in the LV) upon PAB, whereas only 10 genes were deregulated in the LV upon AOB (Fig. 1f, left Venn diagrams). Upon PAB, twice as many genes (224 compared to 127) were deregulated in directly affected ventricles, and only 25% (57 genes) were shared between the compensated and decompensated states (Fig. 1f, right Venn diagrams).

These datasets from two well-defined rat models of chronic RHF or LHF clearly demonstrated (1) the banding model-specific transcriptomic response of the directly affected ventricle adjacent to the banded vessel, (2) disease-specific and ventricle-specific sets of genes and (3) evidence for profound shifts in gene expression programs during the transition from heart hypertrophy to HF that appeared to be stronger in the RV.

### Different gene responses of LVs and RVs to PAB

**Fig. 2:**
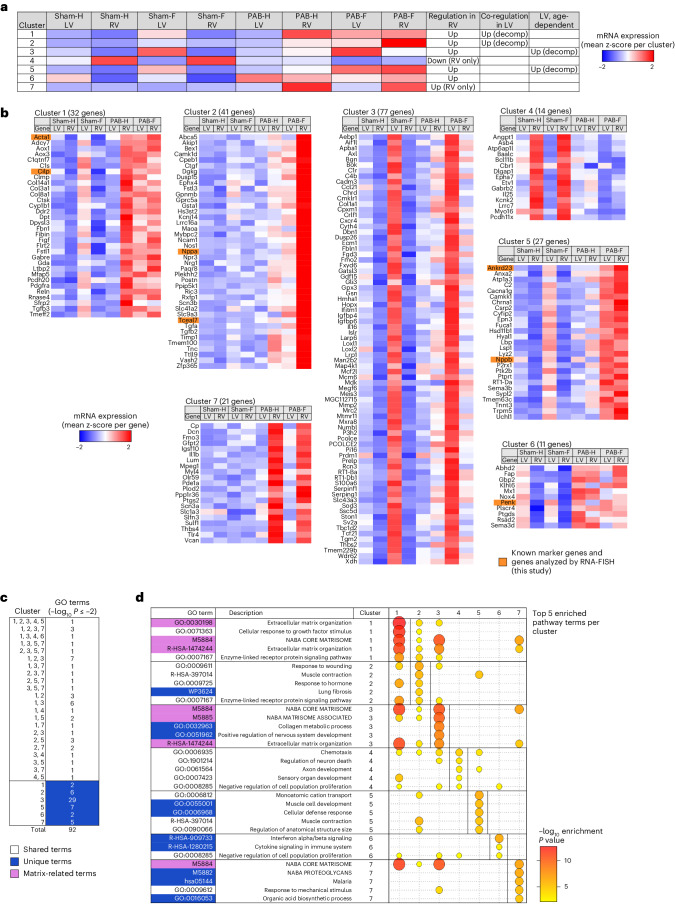
Different transcriptomic responses of LVs and RVs to PAB. **a**, The mRNA expression values of all 224 PAB-regulated genes (Fig. 1f) from RVs and LVs were hierarchically clustered by *k*-means. The heatmap shows the averaged z-score-normalized read counts for all genes belonging to each of the seven clusters across all conditions in both LVs and RVs. The prevailing gene regulatory phenotype is indicated in the three right columns. **b**, Heatmaps of z-scaled mean expression values for all individual 224 genes across the seven clusters and all conditions. Orange colors mark known (*Acta1*, *Cilp*, *Nppa* and *Nppb*) and new PAB-regulated genes that were highlighted in following analyses or further investigated throughout this study. **c**, The genes of each cluster were examined for the top 100 overrepresented pathway terms using Metascape (https://www.metascape.org)^71^, and multiple Venn analysis was performed to identify overlapping and distinct pathway terms for the gene sets of all seven clusters. Only pathway terms with enrichment −log_10_ *P* ≤ 2 were considered. **d**, Graphical representation of the top five enriched pathways for each cluster along with the corresponding enrichment *P* values (highlighted by bubble size and color). If below the threshold, values are also included for the other six clusters. GO, Gene Ontology. Source data

A focused analysis of the 224 cardiomyocyte genes deregulated in PAB conditions revealed seven distinct patterns of gene expression and gene regulation in the RV compared to the LV according to age, RVH and RVF (clusters 1–7; Fig. 2a,b).

Clusters 1 and 2 characterized PAB-induced genes that were strongly upregulated in both the compensated and decompensated RV as well as in the decompensated LV (Fig. 2a,b).

Cluster 3 gene sets were selectively upregulated in the LV of the oldest sham group and comprise age-dependent LV-specific genes. On average, their expression was lower in the decompensating RV but still induced compared to the corresponding RV sham group (Fig. 2a,b).

Cluster 4 comprised the only set of genes that was more highly expressed in the RV (compared to the LV) of the sham groups. These genes were downregulated under PAB conditions (Fig. 2a,b).

Genes from cluster 5 were strongly induced during decompensation in both the LV and RV. Similar to cluster 3 genes, their expression increased strongly only in the LV with age in the corresponding sham group (Fig. 2a,b).

Cluster 6 included genes that were strongly expressed only in the LV of the youngest sham group and whose expression decreased with age. These genes were already strongly induced in the compensated state in both the LV and RV under PAB conditions (Fig. 2a,b).

Finally, cluster 7 included the most specific PAB-induced genes, as their expression increased only in the RV during RVH and RVF (Fig. 2a,b). These genes encoded extracellular matrix (ECM) glycoproteins, such as versican (*Vcan*), decorin (*Dcn*), lumican (*Lum*) and thrombospondin-4 (*Thbs4*). Additionally, this cluster included several factors involved in inflammatory signaling (interleukin 1 beta (*Il1b*), Toll-like receptor 4 (*Tlr4*), prostaglandin G/H synthase 2 (*Ptgs2*) and the enzymes calcium/calmodulin-dependent 3′,5′-cyclic nucleotide phosphodiesterase 1A (*Pde1a*) and procollagen-lysine, 2-oxoglutarate 5-dioxygenase 2 (*Plod2*)), the latter forming hydroxylysine residues in Xaa-Lys-Gly sequences in collagens.

The 224 genes mapped to 92 pathway ontology terms, 51 of which were unique (Fig. 2c, dark blue part of the table). Examples are lung fibrosis (WP3624, cluster 2), muscle development (GO:0006968, cluster 5) and IFNα/β signaling (R-HSA-90973, cluster 6) (Fig. 2d). There was a strong overrepresentation of pathway terms annotated to regulation of ECM in several gene clusters, such as clusters 1, 3 and 7 (Fig. 2d). This result was in line with a progressive, increased deposition of interstitial collagen in representative sections of the heart from PAB and AOB animals (Supplementary Fig. 1).

In summary, these data show that many genes that are activated or repressed in the hypertrophied or failing RV are already highly expressed in the LV in an age-dependent manner and may become additionally co-regulated, especially in the decompensating heart. Examples are known marker genes such as *Acta1*, *Cilp*, *Nppa* or *Nppb* (highlighted in orange in Fig. 2b). Functionally, the different expression patterns of these genes are most likely associated with diverse molecular remodeling processes (including the ECM) that are coordinated at the transcriptome level under PAB conditions.

### Gene regulatory networks in the transition from RVH to RVF

**Fig. 3:**
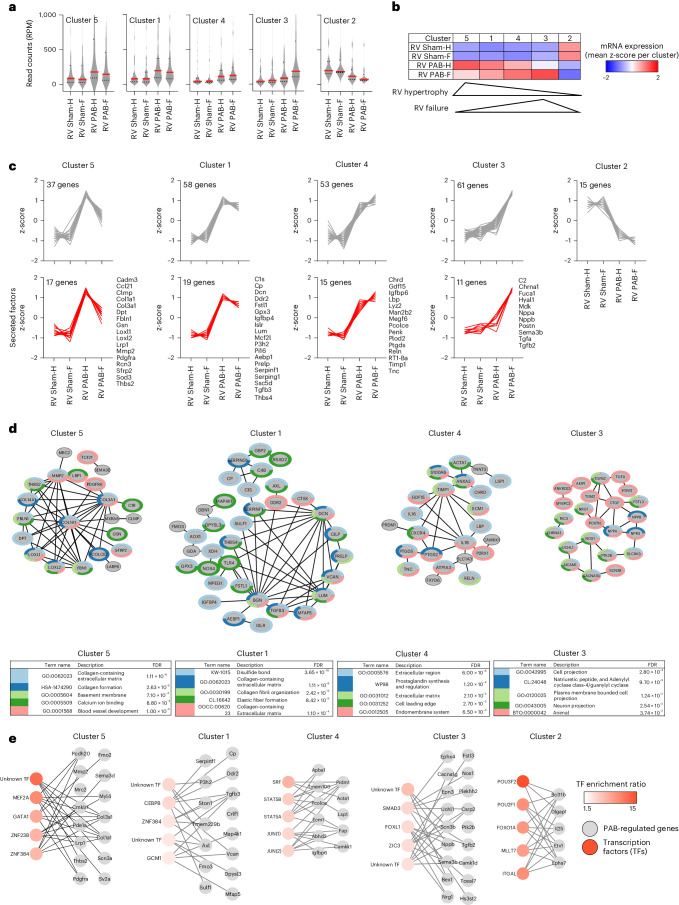
Identification of gene regulatory networks underlying the transition from RVH to RVF. **a**, The mRNA expression values of all 224 PAB-regulated genes from RVs were segregated into five groups by hierarchical *k*-means clustering. Violin plots show all normalized read counts (reads per million (RPM)), medians (solid red lines) and 1st and 3rd quartiles (dotted red lines). **b**, z-scaled heatmap of mean mRNA expression for the genes of the five clusters from **a**. Triangles indicate the overall regulation of clusters 5 and 3, which most specifically characterize the transition from the compensatory to the decompensated states. **c**, Upper panels: z-scaled expression changes of all individual genes per cluster. Lower panels: subgroups of genes from each cluster (also shown by gene name) encoding secreted factors according to a recent annotation of the secreted proteome^18^. **d**, Upper part: physical and functional protein networks for all genes (gray nodes) from each cluster shown in **c** based on STRING database annotations^80^. All PPI categories of STRING (text mining, experiments, databases, co-expression, neighborhood, gene fusion and co-occurrence) and all pathway categories were selected. Depicted are only genes with at least one documented PPI (dark gray edges). All edges had a STRING confidence score greater than 0.4. Lower part: top five enriched pathway terms associated with the genes of each cluster as determined by Cytoscape and the integrated STRING application. Colors of tables and node borders indicate the top five enriched pathway terms associated with each individual gene. FDR indicates the false discovery rate for pathway enrichment. **e**, All genes of each cluster were examined for their overrepresentation in annotated TF gene sets of the MSigDB (https://www.gsea-msigdb.org/gsea/msigdb)^73^. Shown are the top five significantly enriched TFs (reddish nodes) that are connected to their target genes (gray nodes) by gray connecting edges. The complete lists of the top 10 enriched unique TFs and all unique target genes are shown in Supplementary Fig. 2a. The mRNA expression values for these TFs in the rat heart are shown in Supplementary Fig. 2b. Source data

Next, the 224 PAB-regulated genes of the RV only were segregated into five distinct patterns according to their changes during transition from healthy to diseased states (Fig. 3a–c).

Cluster 5, 1 and 4 genes increased strongly upon PAB, whereby cluster 5 and 1 genes were highest at the compensatory state and decreased thereafter. In contrast, cluster 4 genes further increased upon decompensation. Cluster 3 genes were not or not as strongly increased at the compensatory state but were strongly induced in the failing RV. Cluster 2 genes comprised the smallest group of genes, which all were strongly downregulated during RVH and RVF (Fig. 3a–c).

Genes of clusters 1, 3, 4 and 5 (but not cluster 2) encoded 11–19 secreted proteins according to a recently published annotation of the secreted proteome^18^. Examples are *Penk* and *Timp1* (cluster 4) and *Nppa*, *Nppb*, *Tgfa* and *Tgfb3* (cluster 3) (Fig. 3c, lower graphs).

Many genes from clusters 1, 3, 4 and 5 encoded proteins that have known protein–protein interactions (PPIs) and assemble into complex functional interaction networks (Fig. 3d). Annotating individual network nodes with the five most strongly enriched pathway terms revealed multiple terms associated with collagen formation and ECM (clusters 1, 4 and 5) but also terms specific for one cluster, such as prostaglandin metabolism (cluster 4) or calcium ion binding or blood vessel development (both associated with cluster 5) (Fig. 3d).

Genes from clusters 1–5 were overrepresented in gene sets containing annotated binding sites for specific transcription factors (TFs). We identified 32 TFs regulating 106 of the 244 PAB genes (Supplementary Fig. 2a). Exemplified by the top five most enriched TFs (colored in red in Fig. 3e), the genes of each cluster are predicted to be regulated by specific combinations of TFs—for example, the cluster 5 genes *Col1a1*, *Col3a1*, *Myl4*, *Pcdh20*, *Pdgfra* and *Sv2a* are regulated by the TF MEF2a, whereas the cluster 4 genes *Abhd2*, *Camkk1*, *Prdm1*, *Ecm1*, *Fap* and *Igfbp6* are regulated by the TF JUN (Fig. 3e). Seventeen of the predicted TFs were found in the RV, of which *Jun*, *Srf*, *Nlf1*, *Mef2a*, *Cebpb* and *Stat5b* were most strongly expressed, but, overall, their mRNA expression levels changed only weakly during PAB (Supplementary Fig. 2b), suggesting that their predicted contribution to the regulation of PAB-specific genes will involve other levels of molecular regulation, such as post-translational or epigenetic mechanisms.

In summary, the transition phase from compensated to decompensated RHF is associated with the activation of specific, relatively small gene regulatory networks encoding mainly protein networks involved in collagen and matrix metabolism. The time-dependent induction or repression of these genes correlates with different patterns of mRNAs encoding multiple secreted factors. Known biomarkers such as *Nppa* or *Nppb*, together with *Tgfa* or *Tgfb2*, steadily increase in the failing RV (see cluster 3), whereas cluster 5 genes such as *Col1a1*, *Co13A1* or *Pdfgra* already decrease when the compensated situation shifts toward decompensation, suggesting that this group may serve as a novel combination of biomarkers for early deterioration of RV function. Bioinformatic analyses also suggest that gene sets regulated in the same direction are under the control of specific TF networks.

### PAB or AOB regulate largely different sets of genes

Using the same strategies, we addressed the question as to which extent PAB-dependent gene sets would overlap with the 127 genes regulated upon AOB (Fig. 1c).

**Extended Data Fig. 6:**
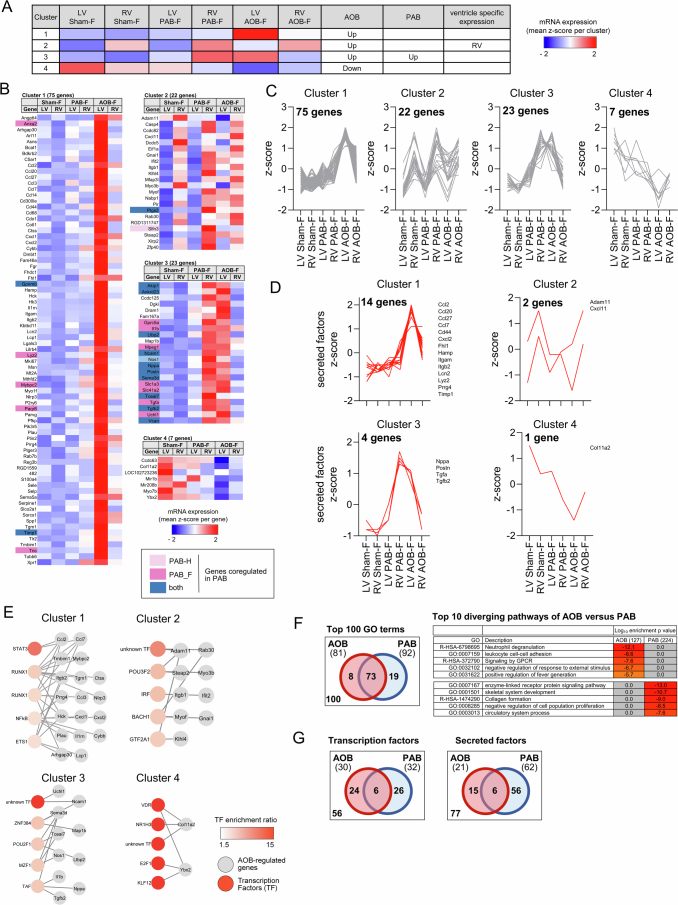
PAB or AOB conditions regulate largely different sets of genes in individual ventricles of the failing rat heart. (**a**) The mRNA expression values of all 127 AOB-regulated genes (see Fig. 1f) from RVs and LVs were segregated into four groups by hierarchical *k*-means clustering. The heatmap shows the averaged Z-score normalized read counts for all genes belonging to each of the four clusters across all indicated conditions in both LVs and RVs. The prevailing gene-regulatory phenotype is indicated in the three right columns. (**b**) Heatmap of Z-scaled mean expression values for all individual 127 genes across the four clusters and all conditions. Pink and blue colours mark PAB-regulated genes. (**c**) Line graphs showing Z-scaled expression changes of al individual genes of each cluster. The numbers of genes for each cluster are indicated in bold font. (**d**) Subgroups of genes from each cluster that encode secreted factors according to a recent annotation of the secreted proteome by^18^. (**e**) All genes of each cluster were examined for their overrepresentation in annotated transcription factor gene sets (TF) of the MSigDB data base. Shown are the top 5 significantly enriched TFs (reddish nodes) which are connected to their target genes (grey nodes) by grey connecting edges. The complete lists of the top 10 enriched unique TFs and all unique target genes are shown in Supplementary Fig. 3a. (**f**) All DEGs from AOB (127 genes) or PAB (224 genes) were examined for overrepresented pathway terms. The Venn diagram shows overlapping and distinct pathways for the top 100 most strongly enriched terms. The heatmap on the right shows the top 5 most strongly enriched terms that were unique for AOB or PAB. The complete lists of the top 100 pathway terms is provided as clustered heatmap in Supplementary Fig. 3b. (**g**) Venn diagrams demonstrating overlapping and distinct transcription and secreted factors identified in the DEG lists of PAB or AOB according to the analyses shown in Fig. 3 and Extended Data Fig. 6. Source data

As shown in Extended Data Fig. 6a–c, AOB genes were segregated into four patterns, of which cluster 1 contained 75 genes that were highly specifically induced in the LV only by AOB, with only seven genes (9%) also being regulated upon PAB. Cluster 2 genes were, on average, weakly downregulated in the LV upon AOB but were constitutively higher expressed in the RV compared to the LV. In contrast, cluster 3 genes largely overlapped between LV upon AOB and RV upon PAB, respectively. This set of genes contained *Nppa,* *Il1b*, *Tceal7, Tgfa* and *Tgfb2*. Interestingly, the inducible expression of these genes was strictly restricted to the corresponding ventricle adjacent to the banded vessel; thus, they increased in the LV upon AOB and in the RV upon PAB. Lastly, cluster 4 genes were strongly downregulated by AOB in the LV and weakly downregulated in the RV upon AOB but not regulated by PAB. These genes, therefore, represent an AOB-specific set that is negatively co-regulated in the LV and RV. Altogether, only 27 (21%) of the 127 AOB genes were also co-regulated in both PAB conditions.

Cluster 1 genes contained 14 secreted factors, including five chemokines (*Ccl2*, *Ccl20*, *Ccl27*, *Ccl7* and *Cxcl2*) and two integrins (*Itgam* and *Itgb2*), which regulate leukocyte trafficking and activation, whereas the other three clusters encoded only seven secreted factors (Extended Data Fig. 6d).

We identified 30 unique TFs suggested to regulate 64 AOB target genes of clusters 1–4 (Extended Data Fig. 6e and Supplementary Fig. 3a).

Of the top 100 enriched pathway terms, 27 were specific for AOB-regulated or PAB-regulated genes, and 73 terms overlapped (Extended Data Fig. 6f and Supplementary Fig. 3b). Three of the unique terms for AOB (R-HSA-6798695, GO:0007159 and GO:0031622) referred to inflammatory processes, consistent with the regulation of secreted inflammatory mediators found in the highly LV-specific and AOB-specific cluster 1 (Extended Data Fig. 6f).

In conclusion, the direct comparison of the AOB with the PAB models indicated major differences between RHF and LHF that manifested at the gene, secreted factor, TF network and pathway levels as shown by the Venn diagrams in Extended Data Fig. 6f,g.

### Spatially heterogeneous regulation of PAB-dependent genes

**Fig. 4:**
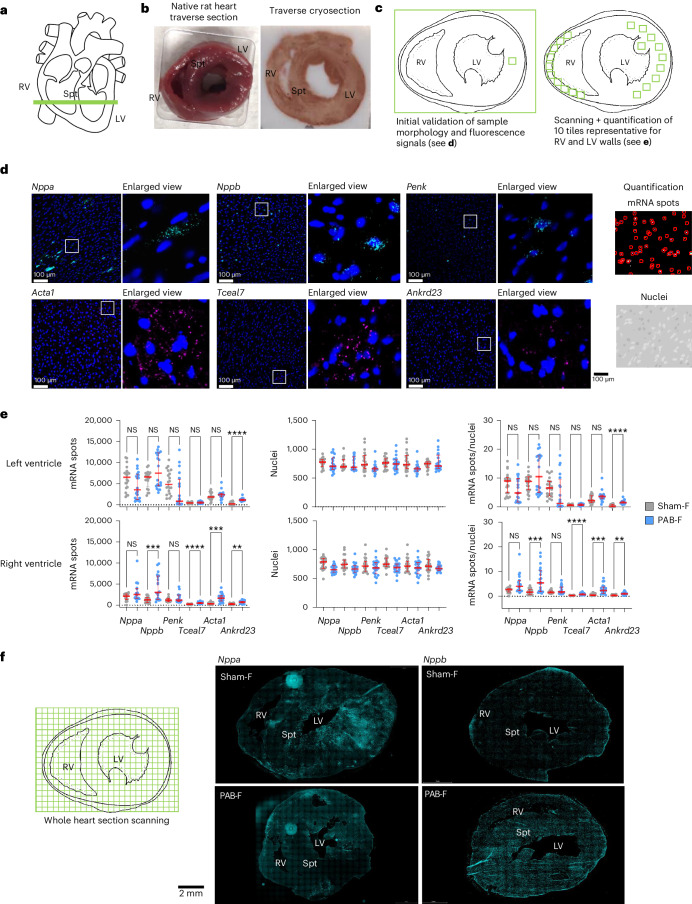
Spatial regulation of PAB-regulated genes in the whole rat heart revealed by RNA-FISH. **a**, Scheme of the location of traverse sections of the heart used for smRNA-FISH. **b**, Rat heart samples before and after processing for cryosections. **c**, Schematic of the strategy for scanning the walls of the RVs and LVs through 10 tiles of equal-sized sections each. **d**, Representative images of smRNA-FISH performed on 7-µm cryosections. Samples were hybridized with pairs of probes for the indicated transcripts, and mRNAs were visualized by two different fluorophores. Nuclei were stained in parallel with DAPI. White inserts are shown as enlarged view on the right side of each image to demonstrate the spatial distribution of mRNA signals in the dense cardiomyocyte cell layers. Right upper and lower panels demonstrate the automated detection and quantification of mRNA spots (upper image) or nuclei (lower image) of each section by Icy software (version 2.4.2.0) (https://icy.bioimageanalysis.org/)^81^. Scale bars indicate 100 µm. Images are representative for one out of two experiments with similar results. **e**, Rat hearts from controls or PAB-operated animals were obtained at the RHF states (Sham-F, PAB-F) and processed for smRNA-FISH as shown above. The graphs show quantification of mRNA spots, nuclei and mRNA spot signals normalized for cell number of each section according to the nuclei counts. Scatter plots show data from 20 sections derived from two biologically independent experiments. Red lines show medians, and whiskers show 1st and 3rd quartiles. Significant changes were identified by one-way ANOVA; asterisks indicate *P* values (*P ≤ 0.05, **P ≤ 0.01, ***P ≤ 0.001, ****P ≤ 0.0001). **f**, Left, schematic strategy for whole heart scans. Right, spatial distribution of *Nppa* and *Nppb* mRNA signals across the whole heart before and after PAB. Scale bar, 2,000 µm. Images are representative for one out of two experiments with similar results. NS, not significant; Spt, septum. Source data

**Extended Data Fig. 7:**
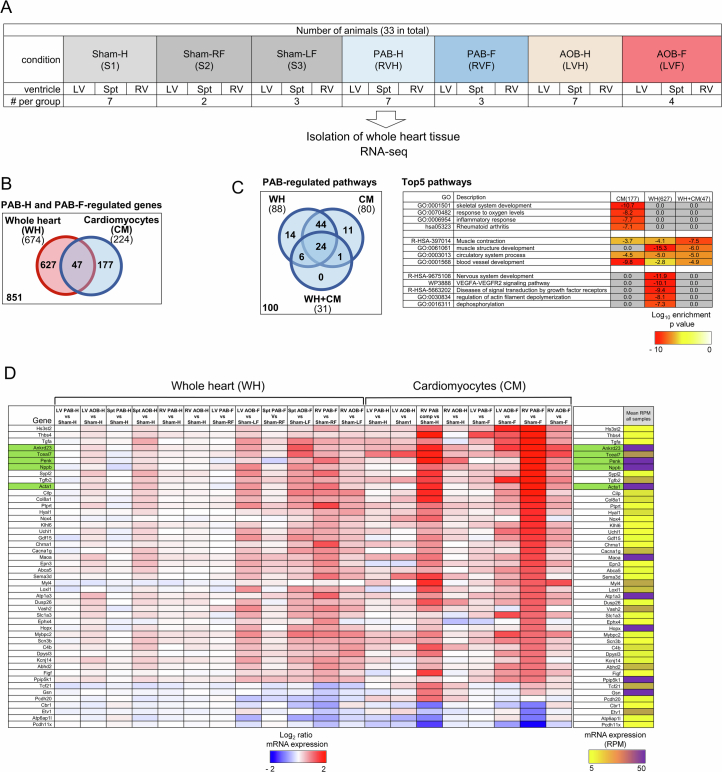
PAB or AOB regulated genes in whole hearts compared to isolated cardiomyocytes. (**a**) Overview of animal study design. (**b**) Whole heart (WH) and cardiomyocyte (CM) RNAseq data sets were normalized together and jointly and differentially expressed genes were obtained by Deseq2 analysis. Venn diagram showing the overlap of the 224 PAB regulated genes (based on the filter criteria shown in Fig. 1f) with 674 PAB-regulated genes (PAB-H and PAB-F combined) obtained from whole heart RNAseq based on significant changes (p ≤ 0.01) of DEGs in the RV. (**c**) All DEGs from whole heart (674 genes) or cardiomyocytes (224 genes) as described in (b) were examined for overrepresented pathway terms using Metascape^71^. The Venn diagram shows overlapping and distinct pathways for the top 100 most strongly enriched terms. The heatmap on the right shows the top 5 most strongly enriched common or unique terms. The complete lists of the top 100 pathway terms is provided in the Source data for Extended Data Fig. 7. (**d**) Heatmap showing fold changes across all conditions as described in (b) and mean overall expression values for all 47 genes jointly regulated in the whole heart or cardiomyocyte samples as described in (b). Source data

To validate DEGs regulated upon PAB, we first subjected LV, RV and septum samples of whole hearts from AOB and PAB animals to RNA-seq, to test the possibility that the isolation procedure of cardiomyocytes contributed per se to the regulation of DEGs (Extended Data Fig. 7a). Second, we examined and quantified the in situ mRNA expression of prototypical PAB-regulated genes by single-molecule RNA fluorescence in situ hybridization (smRNA-FISH) in whole heart sections (Fig. 4).

Forty-seven out of 674 DEGs observed in whole heart samples overlapped with the principal set of 224 PAB-regulated genes of cardiomyocytes (Extended Data Fig. 7b). These genes mapped to common pathways such as muscle contraction and development (R-HSA-397014, GO:0061061), circulatory system (GO:0003013) and blood vessel development (GO:0001568) (Extended Data Fig. 7c). Overlapping genes were more strongly regulated in isolated cardiomyocytes compared to whole heart samples, in line with whole heart transcriptome sequencing reducing the sensitivity for cardiomyocyte-specific DEGs (Extended Data Fig. 7d).

From the 47 genes, we chose *Ankrd23*, *Tceal7*, *Penk*, *Nppb* and *Acta1* for follow-up smRNA-FISH because they were strongly regulated in the RV and highly expressed in RNA-seq analyses, as shown by the heatmap in Extended Data Fig. 7d.

Traverse heart cryosections were hybridized with probes, and 10 representative sections of RV and LV walls were scanned (Fig. 4a–c). *Nppa*, *Nppb*, *Penk* and *Ankrd23* mRNA spots showed remarkably heterogeneous expression patterns, whereas *Acta1* and *Tceal7* mRNA spots were detected more evenly in many cardiomyocytes (Fig. 4d). Quantification confirmed significant PAB-dependent increases in *Nppb*, *Acta1*, *Tceal7* and *Ankrd23* specifically in the RV, whereas *Ankrd23* was the only gene significantly changing also in the LV (Fig. 4e). *Penk* mRNA also increased but only in a part of all sections (Fig. 4e).

Exemplarily mounted whole images from 400 scans of heart sections, covering the entire heart area, corroborated the single-cell variability of gene expression in individual, even adjacent, cardiomyocytes. In sham animals, *Nppa* appeared to be prevalently expressed in the LV, whereas, upon RHF, its expression increased also in more areas of the RV. *Nppb* was only sporadically found in the RV of sham animals, whereas its expression increased in both ventricles upon PAB (Fig. 4f).

Together, whole heart RNA-seq and RNA-FISH confirmed many of the PAB-regulated genes and further indicated that strong peaks of localized gene expression occur in both healthy and diseased hearts.

### Confirmation of rat PAB-regulated mRNAs at the proteome level

**Fig. 5:**
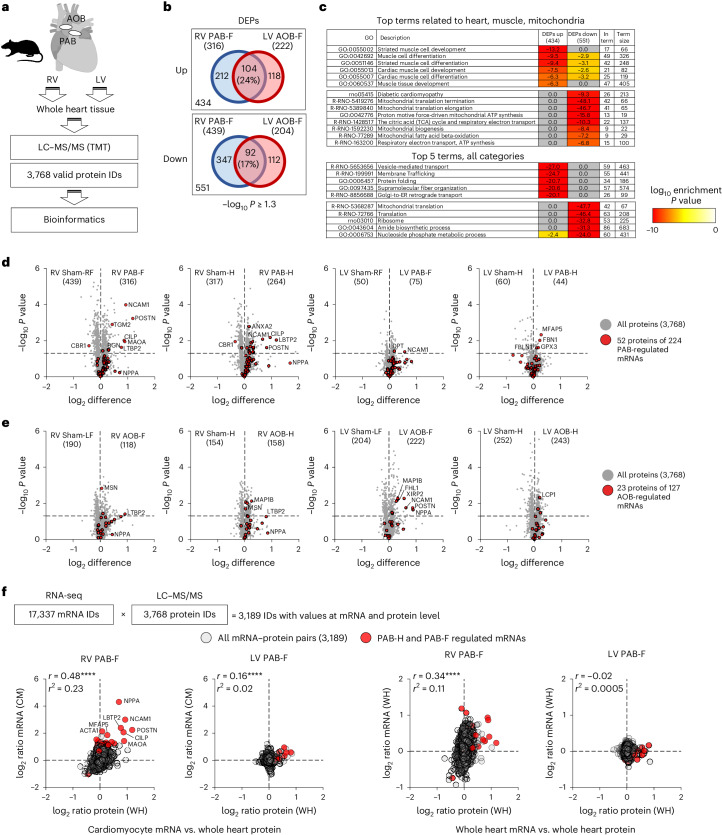
Proteome analysis of common and differentially regulated factors in the RV and LV of PAB and AOB rat models. **a**, Overview of proteomic analyses. Tryptic peptides derived from RV or LV heart tissues of rats subjected to PAB or AOB conditions were labeled with tandem mass tags (TMTs) (*n* = 8 rats per group). In total, 4,149 protein IDs were identified. Scaled, normalized data were log_2_ transformed and width normalized. The data matrix was reduced to 3,768 IDs based on 75% (that is, six out of eight) valid values in at least one group. **b**, Significantly differentially expressed proteins (DEPs) were identified based on pairwise comparisons of AOB or PAB conditions with sham controls using a −log_10_ *P* ≥ 1.3. Venn diagrams show the overlap of DEPs in the RV or LV upon PAB or AOB HF conditions. **c**, All upregulated or downregulated proteins in the PAB or AOB HF conditions as shown in **c** were pooled and analyzed separately for overrepresented pathway terms related to heart, (cardiac) muscle or mitochondria terms (upper table) or for all functional categories. **d**,**e**, Volcano plots show regulation and *t*-test results of all proteins in PAB or AOB conditions compared to the respective sham controls. Red symbols indicate proteins that matched the differentially expressed mRNAs of PAB (**d**) or AOB (**e**) conditions that we identified in Fig. 1f. Numbers in brackets within graphs highlight significantly upregulated or downregulated DEPs (−log_10_ *P* ≥ 1.3). **f**, RNA-seq and proteomic datasets were intersected to identify 3,189 genes with values for both mRNA and protein expression. Graphs show pairwise correlations of PAB-dependent changes for both RV and LV. *y* axes show changes of mRNA levels from cardiomyocytes (left graphs) or whole heart (right graphs), and *x* axes show changes of protein levels in whole heart. Red symbols highlight the factors of the principal set of 224 PAB-dependent genes that were regulated at the mRNA level. Pearson correlation coefficient (*r*) and coefficients of determination (*r*^2^) are indicated. Asterisks (****P ≤ 0.0001) indicate *P* values derived from an *F*-test to test the null hypothesis that the overall slope is zero. CM, cardiomyocyte; GO, Gene Ontology; WH, whole heart. Source data

By proteomic analysis, we identified 3,768 proteins from the rat models (Fig. 5a). Large groups of differentially expressed proteins (DEPs) characterized the different disease conditions, with RV PAB-F and LV AOB-F showing the strongest changes (Fig. 5b and Supplementary Fig. 4a). Multiple upregulated proteins mapped exclusively to (cardiac) muscle-related terms, whereas downregulated proteins were strongly associated with changes in mitochondrial biogenesis or energy metabolism (Fig. 5c, upper table). Further strongly enriched pathway terms were related to intracellular transport, (mitochondrial) translation and amide/nucleoside phosphate metabolism (Fig. 5c, lower table, and Supplementary Fig. 4b). Less than 25% of DEPs overlapped between PAB-regulated or AOB-regulated proteins in RV or LV, indicating that PAB or AOB change the cellular processes associated with HF through different sets of proteins (Fig. 5b,c).

Fifty-two proteins matching to 224 PAB-regulated transcripts identified by cardiomyocyte RNA-seq were also identified at the protein level and were among the most strongly regulated DEPs in the diseased RVs, as exemplified for Ncam1, Postn, Maoa, Cilp or Ltbp2 (Fig. 5d). This was also the case for 23 proteins out of 127 DEGs identified in AOB (Fig. 5e).

PAB-mediated changes of 3,189 factors with values in both transcriptomic and proteomic analyses correlated in the RV but not in the LV (Fig. 5f), and this effect was stronger with DEGs from cardiomyocyte RNA-seq (Fig. 5f, left graphs) compared to DEGs from whole heart RNA-seq (Fig. 5f, right graphs).

In summary, the measurable rat cardiac proteome was dominated by factors regulating muscle adaptation, mitochondrial and energy metabolism-related processes as well as intracellular transport and translation. Ventricle-related analysis revealed differential protein sets between PAB and AOB. Approximately 20% of DEGs identified by cardiomyocyte-specific RNA-seq analysis are recovered at the protein level, and many of these are among the most strongly regulated proteins.

### Functional validation of Penk in isolated rat cardiomyocytes

**Fig. 6:**
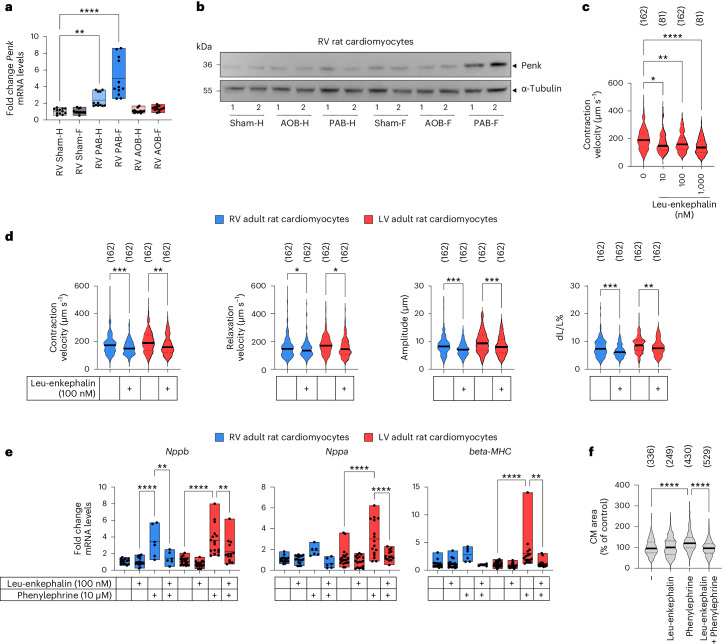
Validation of PAB-dependent regulation of Penk expression and suppression of cardiomyocyte contractility and hypertrophy by opioid peptides. **a**, *Penk* mRNA expression in RV cardiomyocytes isolated from sham, PAB or AOB conditions was analyzed by RT–qPCR. Box plots show data points from all individual animals with means and minimum/maximum values. Asterisks indicate significant changes according to one-way ANOVA (*P ≤ 0.05, **P ≤ 0.01, ***P ≤ 0.001, ****P ≤ 0.0001). Graphs show all values, including technical duplicates from six biologically independent cardiomyocyte preparations. **b**, Cell extracts were isolated from pooled RV cardiomyocytes of 6–8 animals per condition and analyzed by western blotting for the expression of Penk. Two independent samples (1 and 2) per condition are shown. Antibodies against tubulin served as a loading control. **c**,**d**, Isolated RV or LV cardiomyocytes were paced at 2 Hz. Contraction or relaxation velocity, contraction amplitude and load-free cell shortening (expressed as dL/L (%)) were assessed. **c**, Dose response of LV cardiomyocytes treated with Leu-enkephalin for 10 min. **d**, Decreased contractility parameters of RV or LV cardiomyocytes treated with 100 nM Leu-enkephalin for 10 min. Total cell numbers from six independent cardiomyocyte preparations are shown in brackets. **e**, Isolated RV or LV cardiomyocytes were stimulated with 10 µM phenylephrine for 24 h in the presence or absence of 100 nM Leu-enkephalin. Expression of hypertrophy marker genes was analyzed by RT–qPCR. Changes of mRNA levels were calculated relative to the mean of all untreated controls. Box plots show all data points (including technical replicates) with means and minimum/maximum values obtained from four independent cardiomyocyte preparations. Asterisks indicate significant changes according to one-way ANOVA (*P ≤ 0.05, **P ≤ 0.01, ***P ≤ 0.001, ****P ≤ 0.0001). **f**, Relative changes of cardiomyocyte area size under the conditions described in **e**. Total cell numbers from four independent cardiomyocyte preparations are shown in brackets. Solid lines in violin plots in **c**, **d** and **f** show medians; dotted lines show 1st and 3rd quartiles. Significant changes were identified by one-way ANOVA; asterisks indicate *P* values (*P ≤ 0.05, **P ≤ 0.01, ***P ≤ 0.001, ****P ≤ 0.0001). CM, cardiomyocyte. Source data

We chose *Penk* (proenkephalin) as an example for further in-depth validation of factors arising from the large-scale analyses, as the role of the opioid system in RV function is obscure^19^. Both mRNA and protein analysis confirmed a particularly strong upregulation of Penk in RV cardiomyocytes upon PAB (Fig. 6a,b). Penk protein is proteolytically processed into multiple opioid receptor agonistic peptides, including the pentapeptides Met-enkephalin and Leu-enkephalin, which activate µ-opioid and δ-opioid receptors in various parts of the body^20^. Leu-enkephalin dose-dependently suppressed contraction velocity and inhibited all additional functional parameters assessed in adult rat cardiomyocytes isolated from both the LV and the RV (Fig. 6c,d). Leu-enkephalin further suppressed the α1-adrenergic induction of LV or RV cardiomyocyte hypertrophy marker genes and prevented phenylephrine-induced increase in cardiomyocyte size (Fig. 6e,f). These data suggest that elevated Penk levels may contribute to anti-hypertrophic effects and reduced cardiomyocyte contractile function in failing RVs.

### Overlap of rat and human gene sets in right heart disease

**Fig. 7:**
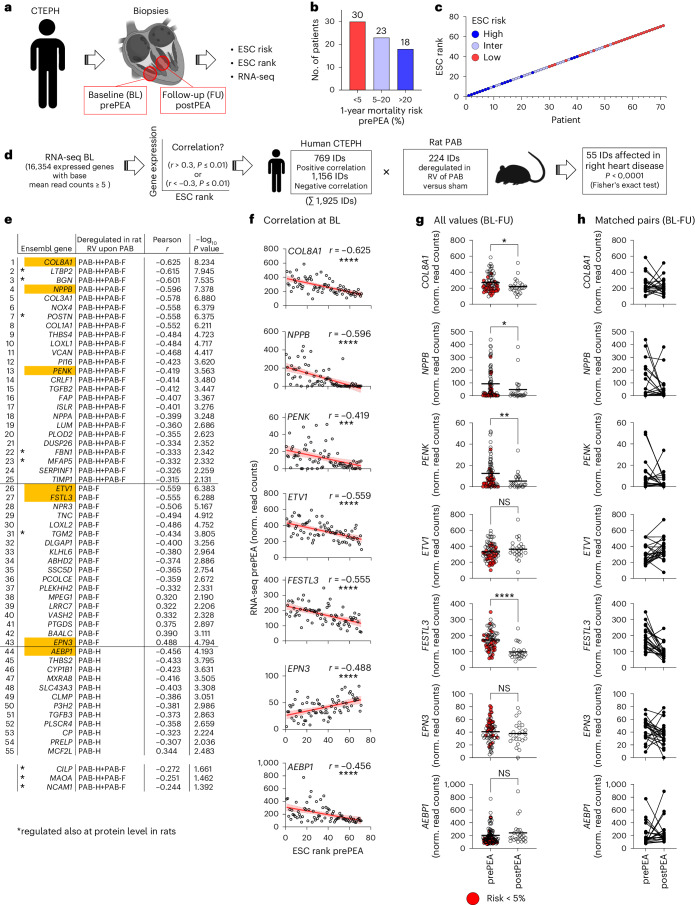
Rat PAB gene sets are deregulated in patients with CTEPH, and their expression levels correlate with disease severity. **a**, CTEPH patient cohorts and sample generation for RNA-seq. At baseline (BL, prePEA), RV wall tissues were collected during thoracic surgery of 71 patients. At follow-up (FU, postPEA), septum samples from 24 patients were obtained by right heart catheter. Clinical parameters were used to group patients at prePEA state according to the 1-year mortality risk (ESC risk) and rank them by disease severity (ESC rank) based on criteria of the European Society of Cardiology^21^. **b**, Proportion of patients prePEA with high, intermediate or low mortality risk. **c**, Relation of ESC rank to patient risk at prePEA state. **d**, Strategy to define expressed genes (IDs) at prePEA state that correlate significantly with ESC rank (Pearson *r* > or < 0.3 and *P* ≤ 0.01). This set of 1,925 IDs was intersected with the 224 PAB-regulated genes from the rat model (Fig. 1f), resulting in a significant (Fisher’s exact test, *P* < 0.0001) overlap of 55 genes. **e**, Fifty-five genes overlapping between rat and human RHF datasets, including Pearson correlation coefficients and *P* values at BL. Additionally, values for *CILP*, *MAOA* and *NCAM1* are shown. Asterisks mark genes with confirmation of regulation in rat PAB at the protein level (Fig. 5). **f**, Correlation of mRNA expression ((normalized (norm.) read counts)) with ESC rank at prePEA state for prototypical genes (marked in orange in **d**). The graphs display values for 71 patients, linear regression lines (in red), 95% confidence intervals (in gray), Pearson *r* and *P* values (*P ≤ 0.05, **P ≤ 0.01, ***P ≤ 0.001, ****P ≤ 0.0001). **g**, mRNA expression of genes from **f** at time of surgery (prePEA, *n* = 71) and FU (postPEA, *n* = 24). Red colors mark values from patients with lowest mortality risk. Black lines show means, and asterisks indicate significant changes (Mann–Whitney test, *P ≤ 0.05, **P ≤ 0.01, ***P ≤ 0.001, ****P ≤ 0.0001). **h**, mRNA expression values for 22 patients with paired samples at BL and FU. NS, not significant. Source data

To explore the conservation and relevance of the PAB-regulated gene sets for humans, we analyzed 95 RNA-seq datasets from patients with CTEPH. RV biopsies were obtained during thoracic surgery at baseline (BL, prePEA) and for 24 patients also from the septum (by right heart catheter) during follow-up, 12 months after PEA (FU, postPEA) (Fig. 7a). Disease severity of the patients at BL was scored based on cardiac index, TAPSE/systolic pulmonary arterial pressure (sPAP) and N-terminal pro-brain natriuretic peptide (NT-proBNP) in line with European Society of Cardiology (ESC) guidelines^21^. Patients were stratified into low, intermediate and high 1-year mortality risk groups (Fig. 7b) and ranked for disease severity by equally weighting these parameters, so that higher ranks correlated with lower mortality (Fig. 7c).

Of 16,354 genes that were expressed at BL, 1,925 correlated either negatively or positively with ESC rank (Fig. 7d). In this dataset, 55 genes overlapped with 224 PAB-regulated genes (Fig. 7d,e and Source Data Fig. 7).

Most genes showed negative correlation, such as *COL8A1*, *NPPB*, *PENK*, *ETV1*, *FSTL3* and *AEBP1*, indicating that their levels increased with more severe RHF (Fig. 7e,f). Only seven genes, such as *EPN3*, showed a positive correlation, indicating that their expression might be beneficial (Fig. 7e,f). *CILP*, *MAOA* and *NCAM1* mRNA changes prePEA were also significantly correlated with ESC rank but were below the stringent filtering criteria applied to the top 55 genes (Fig. 7e). Nine of these factors were confirmed to be regulated at the protein level in rats, as shown in Fig. 5.

There was a significant reduction of mean *COL8A1*, *NPPB*, *PENK* and *FSTL3* mRNA levels at FU (Fig. 7g), whereby data from patients at low risk were mostly below the average already at BL (see red dots in Fig. 7g). This was not the case for *ETV1*, *EPN3* and *AEBP1* (Fig. 7g). Similarly, gene expression of 22 available paired samples showed a mixed pattern (Fig. 7h), in line with the interpretation that averaged mRNA expression data obtained from two different regions of the heart (RV wall and septum) in a clinically heterogeneous cohort of patients are more variable and have limited sensitivity to reflect the course of disease.

A further comparison to human HF transcriptomic data was performed by intersecting the rat PAB-regulated and human CTEPH (hCTEPH) patients’ genes with 4,238 significant genes extracted from 14,041 genes across 16 human LHF (hLHF) studies^15^.

**Fig. 8:**
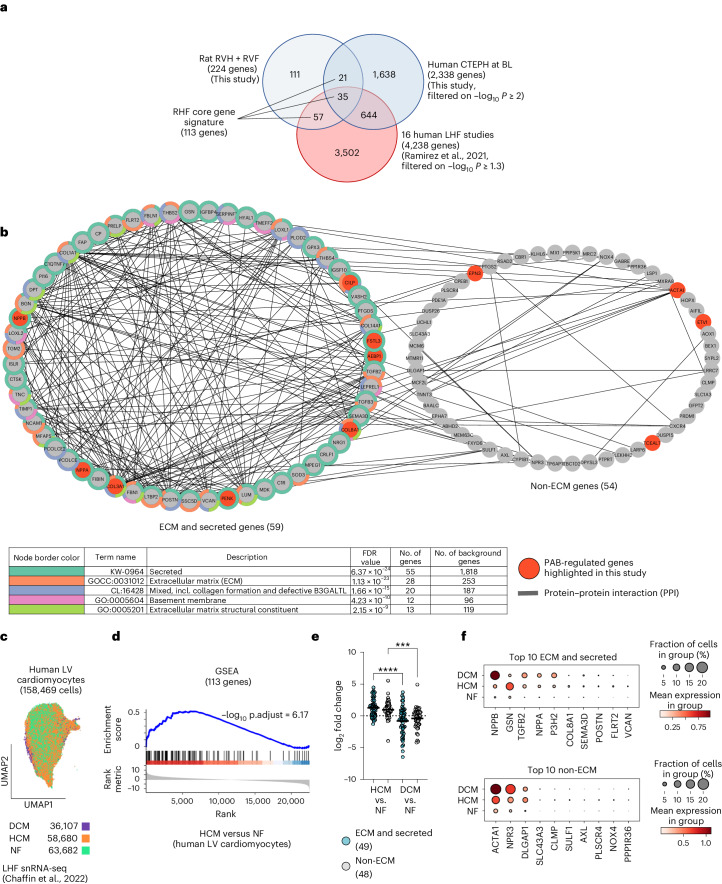
Meta-analyses of rat and human gene sets to derive a core gene signature for RHF. **a**, Intersection of 224 rat PAB-regulated genes with 2,338 genes of hCTEPH patients correlating significantly with ESC at BL (*P* values of −log_10_ ≥ 2; Fig. 7d) and with all 4,238 significant genes (−log_10_ meta-analysis Benjamini–Hochberg *P* ≥ 1.3) extracted from a total of 14,041 genes across 16 hLHF studies^15^. The overlapping 113 genes constitute a core signature for RHF. **b**, The 113 RHF core signature genes were examined for physical and functional protein networks for all genes (nodes) using STRING^80^. Based on the top five enriched pathways (colored in the table and the node borders), the network was split into two parts, representing 59 ECM and secreted or 54 non-ECM components, respectively. FDR indicates the false discovery rate for pathway enrichment. **c**, Uniform manifold approximation and projection (UMAP) plots of published snRNA-seq data of 158,469 LV cardiomyocytes from humans with DCM (*n* = 11) or HCM (*n* = 15) or from NF hearts (*n* = 16)^22^. **d**, Using a pseudo-bulk approach, data from **c** were ranked by differential gene expression comparing HCM with NF and subjected to GSEA with the 113 RHF core gene signature as the interesting set. The adjusted *P* value (p.adjust) indicates significant enrichment of these genes (shown by the blue line) in cardiomyocytes of HCM conditions. **e**, Data from **d** were filtered to identify significantly expressed components (mean reads > 5) of the two networks shown in **b**, resulting in 49 factors representing ECM and secreted components and 48 factors of the non-ECM network. The graphs show mean relative changes for each gene in cardiomyocytes comparing disease states with NF. Black lines show means, and asterisks show significant differences between HCM and DCM (one-way ANOVA, ***P ≤ 0.001, ****P ≤ 0.0001). **f**, Dot plots illustrating the fraction of cardiomyocytes (dot size) and the mean expression (color scale) for the top 10 regulated components of HCM conditions according to the ECM and secreted or the non-ECM groups. Source data

Only 92 (2.2%) hLHF genes overlapped with PAB-regulated genes, whereas the overlap was 679 (16%) between hCTEPH and hLHF, consistent with the notion that RHF and LHF are largely regulated by distinct transcriptomic responses (Fig. 8a).

In total, 113 genes overlapped between rat and human gene sets, including many that were highlighted in our study (marked with orange color in Fig. 8a,b). The fact that these genes were also found in the large hLHF dataset is consistent with severe LHF also causing eventually RHF in some patients.

The RHF-associated genes were separated into two large functional and physical protein interaction networks (Fig. 8b). Fifty-nine components show multiple interactions and are most highly enriched for secreted factors involved in the regulation of the extracellular, collagen-containing matrix (Fig. 8b, left network), whereas the remaining 54 components show fewer interactions, indicating that they have different functions, and their roles in RV disease still need to be defined (Fig. 8b, right network).

**Extended Data Fig. 8:**
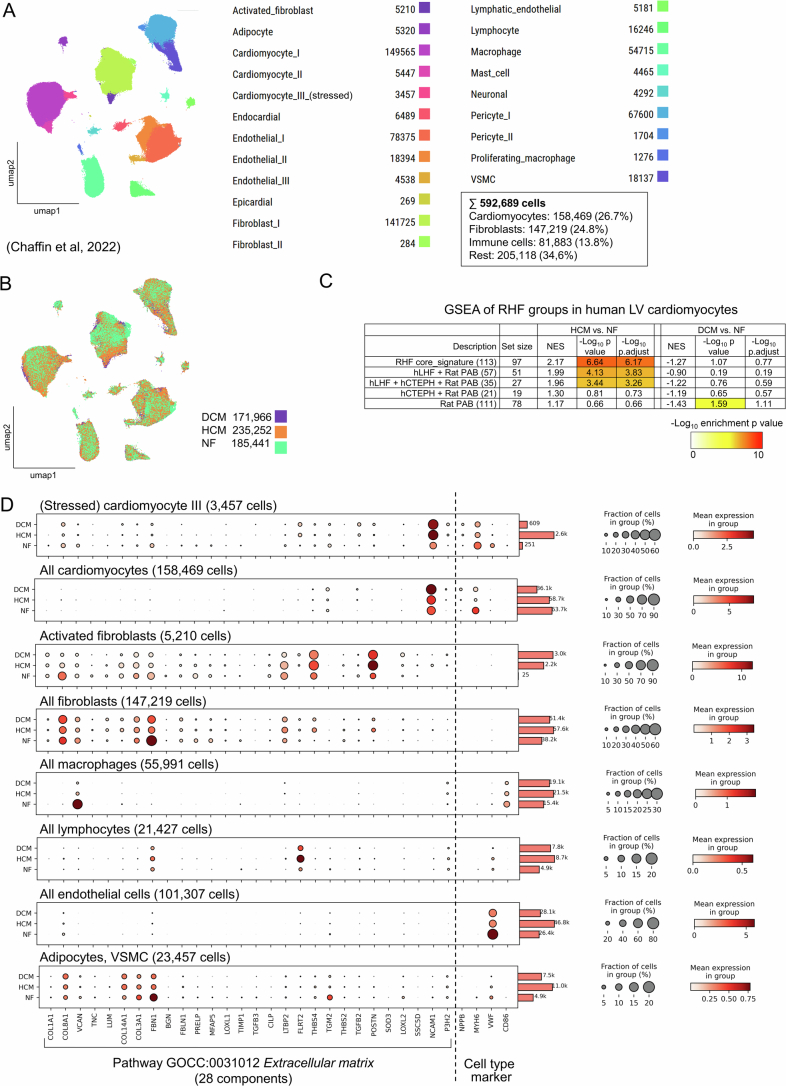
Cardiac cell type specificity of RHF gene signatures. (**a-b**) Uniform manifold approximation and projection (UMAP) plots of snRNAseq data of LV heart samples retrieved from humans with dilated cardiomyopathy (DCM, n=11), hypertrophic cardiomyopathy (HCM, n=15) or from non-failing hearts (NF, n=16). Data were downloaded from the Broad Institute’s Single Cell Portal using the identifier SCP1303 (https://singlecell.broadinstitute.org/single_cell/study/SCP1303/.) (Chaffin et al.^22^). (a) Shows identified cardiac cell types and (b) shows the distribution of cell types according to disease or NF conditions. (**c**) Data from all cardiomyocytes (Cardiomyocytes I-III) were aggregated to pseudo-bulk RNAseq data sets and ranked by differential gene expression comparing HCM or DCM with NF using Deseq2. Ranked lists were subjected to gene set enrichment analysis (GSEA) with all gene sets overlapping with rat PAB as shown in the Venn diagram of Fig. 8a. Genes with mean read counts < 5 were excluded, resulting in the indicated set sizes. Coloured, adjusted p values (p.adjust) indicate significant enrichments in cardiomyocytes according to HCM or DCM conditions. NES, normalized enrichment score. (**d**) Dot plots illustrating per group, the fraction of cardiac cell types expressing a gene (dot size), the mean expression of the gene (color scale) and the number of cells (bars) for 28 components pathway of GOCC:0031012 *Extracellular matrix* (as shown in the table of Fig. 8b). Source data

The partial overlap with the hLHF datasets allowed a refined mapping of gene sets to individual cardiac cell types, leveraging recent snRNA-seq data from healthy donors with non-failing hearts (NF) and patients with LV hypertrophic cardiomyopathy (HCM) or dilated cardiomyopathy (DCM)^22^ (Fig. 8c and Extended Data Fig. 8a,b). Gene set enrichment analysis (GSEA) showed that the 113 RHF core signature genes were significantly overrepresented in cardiomyocytes isolated from patients with HCM but not DCM (Fig. 8d and Extended Data Fig. 8c). In contrast, the 111 rat-specific RHF genes from the Venn diagram shown in Fig. 8a were not enriched in HCM and DCM cardiomyocytes, consistent with this part of the RHF gene signature being right heart specific (Extended Data Fig. 8c). Aggregate or gene-based visualization of the components from the two networks shown in Fig. 8b confirmed that cardiomyocytes inducibly express various ECM and secreted factors as well as non-ECM components in an HCM-dependent or DCM-dependent manner (Fig. 8e,f).

**Extended Data Fig. 9:**
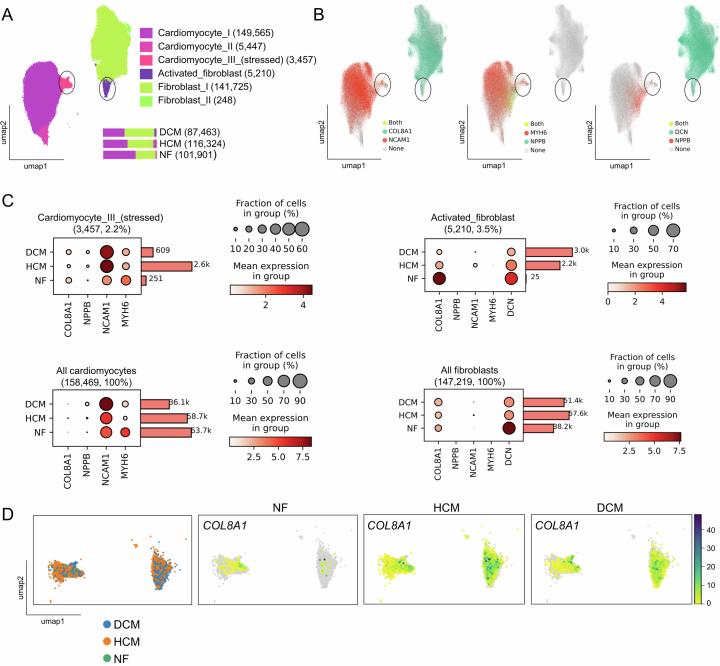
*COL8A1* expression in the activated cardiomyocyte and fibroblast niches. (**a**) Umap plots of cardiomyocyte and fibroblast subpopulations defined in hLHF^22,23^. Black circles mark the stressed cardiomyocyte and activated fibroblast fractions. (**b, c**) Umap (B) and dot plots (C) showing differential distribution of heart failure marker *NPPB* (natriuretic peptide B), ECM factors *NCAM1* (neural cell adhesion molecule 1) and *COL8A1* (collagen type VIII alpha 1 chain) along with cardiomyocyte (*MYH6*, myosin heavy chain 6) or fibroblast marker genes (*DCN*, decorin) in stressed / activated cardiomyocytes and cardiac fibroblasts compared with all cardiomyocytes or fibroblasts found in the LV. (**d**) Umap plots showing the distribution of *COL8A1* in the stressed cardiomyocyte and activated fibroblast fraction according to disease states.

At the example of 28 components of pathway GOCC:0031012 ‘Extracellular matrix’ (see table of Fig. 8b), we found that a small fraction of stressed (or activated) human cardiomyocytes defined by Chaffin et al.^22^ represents a potential source of ECM-related genes, such as *COL8A1*, *VCAN* and *FBN1* (Extended Data Fig. 8d). The expression patterns are distinct from a similarly sized small group of activated human fibroblasts and from all cardiac fibroblasts, which abundantly express ECM genes (Extended Data Fig. 8d). Adipocytes and vascular smooth muscle cells, but not immune cells (macrophages and lymphocytes) or endothelial cells, may also contribute to ECM gene expression of GOCC:0031012 to varying degrees (Extended Data Fig. 8d). The small, stressed (or activated) cardiomyocyte subpopulation represents 2.2% (3,357) of all cells and is distinct from *NPPB*-positive cardiomyocytes (Extended Data Fig. 9a,b). Stressed cardiomyocytes increase by 10-fold in HCM, whereas activated fibroblast subpopulations increase strongly in both HCM and DCM (Extended Data Fig. 9c). These cardiomyocytes appeared to adopt specific cellular states, characterized, for example, by the increased expression of *COL8A1*, one of most strongly regulated genes in rat PAB and hCTEPH (Extended Data Fig. 9d). They may, therefore, together with specific fibroblast subpopulations, be part of specialized cardiac niches, according to recent observations^23^. Collectively, the analyses of available snRNA-seq data provide additional support for cardiomyocytes as a source of the RHF genes identified in our study in response to pressure overload.

In conclusion, these data identified a unique set of RVH-related and RVF-related genes that overlapped between the rat PAB models and human RV disease. Many of these genes show a striking (mostly negative) correlation with disease severity in human patients. Therefore, akin to the study by Ramirez-Florez et al.^15^ for LHF, these genes define a first version of an RVF core gene signature.

### Sex-specific differences of RHF signature genes

**Extended Data Fig. 10:**
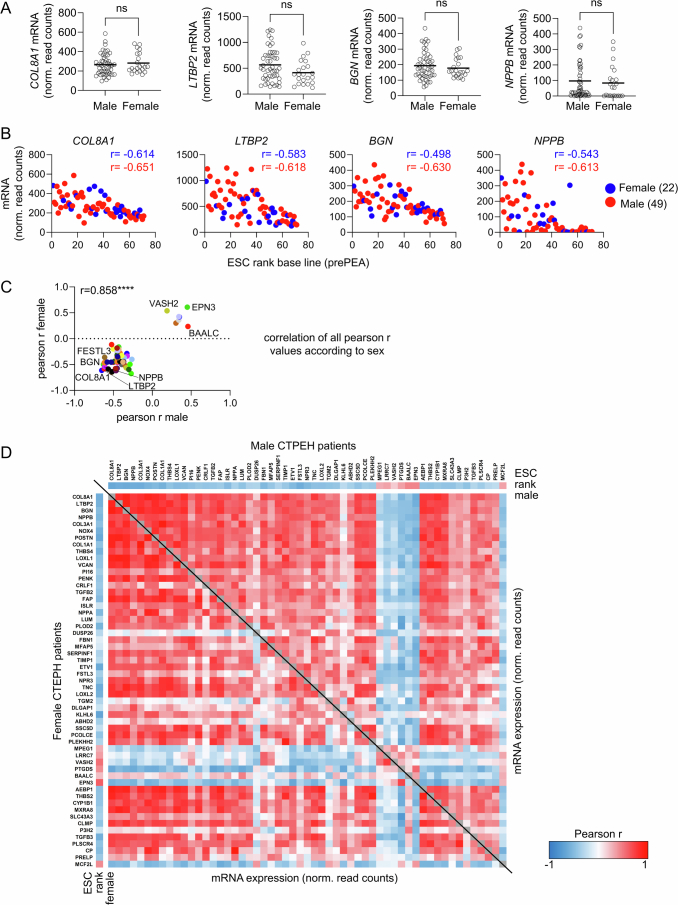
Sex-specific regulation of CTEPH-associated mRNAs. (**a**) Mean expression levels of four exemplary PAB-regulated genes in 71 human CTEPH patients (49 males, 22 females) before surgery (BL, prePEA) according to sex. Graphs show individual values, means and the results from Mann Whitney t-tests. ns, non significant difference between means. (**b**) Correlation of mRNA expression of the genes shown in (a) with ESC rank according to sex. Blue colors mark female and red colors mark male samples. Sex-specific Pearson correlation coefficients are shown in each graph. (**c**) Pearson r correlation coefficients for all 55 PAB-regulated genes that were also found in human CTEPH patients (see Fig. 7e) were calculated separately for male and female patients and correlated with each other for each gene, demonstrating a high a correlation between male and female samples (r=0.858, **** p ≤ 0.0001). (**d**) Aggregated sex-specific correlation matrices for ESC ranks and mRNA expression values for the 55 genes identified in Fig. 7e. Source data

In rat cardiomyocytes, nine (45%) out of 20 clusters of the 224 PAB-regulated genes and two (17%) out of 12 clusters of the 127 AOB-regulated genes showed sex-specific differences in the expression of individual genes (Supplementary Fig. 5a,b). Fifty-eight PAB-regulated proteins that matched mRNA changes showed significant sex-dependent differences in the RV, unlike the 25 proteins deregulated in the LV upon AOB (Supplementary Fig. 6a–c). Correlation analysis of all conditions revealed no general sex-specific differences in the rat heart proteomes but confirmed some differences between male and female samples in the failing RV (Supplementary Fig. 6d). No differences between male and female patient samples were observed in the genes overlapping between rats and humans, as exemplified for four individual genes and confirmed across all 55 genes (Extended Data Fig. 10a–d). Thus, although the highly standardized animal models suggest some sex-specific differences in the regulation of RV key HF genes, the significance for human RV disease needs to be investigated in much larger patient cohorts that are appropriately powered for this aspect.

## Discussion

In this study, systematic comparisons of cardiomyocyte genes deregulated in rat PAB models with (1) AOB conditions, (2) deregulated genes in patients with CTEPH and (3) published meta-analyses of gene lists from LHF studies in patients led to the identification of a set of common genes and gene regulatory networks that, we think, defines an RVF core gene signature.

A predominant observation relates to the altered mRNA expression of a large number of factors of the ECM in the diseased RV, already at the compensated state.

Genes encoding specific fibrillar type I and III collagens (*Col1a1* and *Col3a1*) were among the most strongly (up)regulated ECM factors in both rat models and patients with CTEPH.

Fibrillar collagens (types I, II, III, V, XI, XXIV and XXVII) provide three-dimensional frameworks for tissues and organs that confer mechanical strength as well as signaling and organizing functions through binding to cellular receptors and other components of the ECM^24^. Collagen I (Col I) and Collagen III (Col III) are abundant in the heart, whereby their ratios vary across species and with age^25,26^. Multiple studies have revealed changes in Col I and Col III levels in different models of LV diseases (reviewed in ref. ^27^).

Additionally, the non-fibrillar collagen-encoding gene *Col8a1* was strongly upregulated in both rats and humans^28^. Double-deficient mice for the network-forming collagen type VIII (encoded by the *Col8a1* and *a2* genes) exhibited increased mortality after AOB, and the data suggested that Col 8 protects from early mortality and LV dilatation in response to pressure overload by promoting myofibroblast differentiation and fibrosis^29^. Mice lacking *Col8* had reduced baseline blood pressure and altered ECM composition of carotid arteries, suggesting that Col 8 also negatively regulates elastic fiber formation in the ECM of blood vessels^30^.

The transforming growth factor (TGF)-β isoforms *Tgfb2* and *Tgfb3* were among the core RV-regulated genes. The heart harbors latent TGF-β in the ECM, which is released upon cardiac injury and plays an important role in heart remodeling and fibrosis^31,32^. The highly homologous isoforms TGF-β1, TGF-β2 and TGF-β3 share a receptor complex and activate similar signaling pathways but execute distinct expression patterns and specific functions in vivo^31^. Isoform-specific roles in heart function remain less understood, because previous studies have largely focussed on TGF-β1 (refs. ^33,34^). Additionally, TGF-β activates *Nox4*, another gene of the RV core signature, that was identified as a major source of oxidative stress in failing hearts^35–37^.

The ECM factors latent-transforming growth factor beta-binding protein 2 (LTBP2), periostin (POSTN) and versican (VCAN) were also found recently to be regulated in RV heart biopsies from patients suffering from pulmonary arterial hypertension (PAH), providing independent support of our results at the protein level in humans. This study also validated the regulation of LTBP-2 and COL18A1 and COL6A3 proteins in plasma from patients with PAH and suggested ECM factors as biomarkers for right heart disease^38^.

A particularly interesting question relates to the cellular sources of ECM factors, which were recently attributed largely to fibroblasts in stressed cardiac niches, which was supported by previous studies in mice with fibroblast-specific deletion of ECM genes^23,39^. Cardiomyocytes and fibroblasts represent approximately 50–60% of all cardiac cells in similar proportions, with cardiomyocyte numbers decreasing in disease (Extended Data Fig. 8)^22,40^. We cannot formally exclude the possibility that small amounts of contaminating fibroblasts contributed to the ECM signatures derived from the highly enriched rat cardiomyocyte preparations. However, our re-analysis of human snRNA-seq data from Chaffin et al.^22^ showed that an albeit small number of re-programmed cardiomyocytes, primarily activated in diseased hearts, may contribute to the expression of at least some ECM genes.

We also found multiple non-ECM genes that had not been previously implicated in RHF.

*Penk*, the gene encoding endogenous opioid receptor agonistic peptides, has long been known to be highly expressed in cardiac cell types^19,41^. In clinical studies of LHF, PENK plasma levels were prognostic for all-cause mortality and HF rehospitalization^42^. Recent trials, including the large Prevention of Renal and Vascular End-Stage Disease (PREVEND) study, led to the suggestion of a common PENK-dependent inter-organ pathway that affects both the left heart and the kidneys and in which high PENK levels might be detrimental, might reflect a counter-regulatory response of an over-activated opioid system or might be both initially protective and later on maladaptive^43–45^. Little is known about a (specific) role of Penk and the opioid system in RVH and RVF, but the highly consistent upregulation of *Penk* mRNA in RHF observed in our study and the in vitro suppressive role of Leu-enkephalin on cardiomyocyte contractility strongly suggest that its mechanistic role, the contribution to (mal)adaptive processes and the potential use as an early biomarker for RHF should be analyzed in the future, including in vivo studies of *Penk*-deficient animal models.

*Tceal7*, a poorly characterized member of the transcription elongation factor A (SII)-like family of genes, was discovered as a tumor suppressor^46^. TCEAL7 repressed cyclin D1 transcription, DNA binding activity of Myc and upregulation of Myc target genes^47^. *Tceal7* was also upregulated transiently after cardiotoxin-mediated skeletal muscle injury in mice during muscle differentiation^48^. *Tceal7* was transcriptionally induced by myogenic regulatory factors (MRFs—for example, Myod, Myf5 and Myf6) and suggested to repress myogenic proliferation in favor of muscle cell differentiation^48,49^.

The *Ankrd23* gene (encoding diabetes-related ankyrin repeat protein (DARP)) was isolated from hearts of diabetic mice^50^. Together with cardiac ankyrin repeat protein (CARP/Ankrd1) and ankyrin repeat domain protein 2/ankyrin repeat protein with PEST and proline-rich region (Ankrd2/Arpp), Ankrd23/DARP was classified as a member of stress-inducible muscle ankyrin repeat proteins (MARPs)^51^. The three MARP proteins are co-expressed in striated muscle tissues and share conserved motifs that interact with the giant filamentous titin polypeptide N2A segment to regulate stretch or stress responses^52^. MARPs localize also to the nucleus, and their expression is induced upon injury and hypertrophy (Ankrd1), stretch or denervation (Ankrd2) and during recovery after starvation (Ankrd23), but so far no LV cardiac phenotypes have been found in triple knockout mice after 14 d of TAC^51,52^.

We conclude that ample evidence implicates multiple ECM components in pathological remodeling of LV and blood vessels, emphasizing the necessity to study their specific functional contribution also to RVF^53^. The examples of *Penk*, *Tceal7* and *Ankrd23* highlight factors with some evidence for a function in muscle differentiation or LV heart diseases, which, however, so far have not been investigated for their specific roles in right heart (patho)physiology.

The bioinformatics network analyses at the levels of TFs, pathways and protein interactions further suggest that the RVF core signature genes do not function in isolation but should, rather, be viewed as highly adapted gene regulatory networks whose composition changes spatially and over time during the course of RVH and RVF.

Our study also has some limitations. Further investigations, which were beyond the scope of our current work, are required to track the local origin and quantitative contribution of cardiac cell types to RV core gene signature genes and to prove their functions in vivo. The list of 113 RV core gene signature genes resulted from the adoption of relatively rigorously defined filtering criteria based on a combination of relative expression changes, expression levels and *t*-test criteria, but, as with any bioinformatics analysis, it is clear that softening or tightening these filtering criteria would increase or decrease the number of disease-dependent regulated genes in the RV. Similarly, the annotation of DEGs as secreted, TF-regulated or protein network is dependent on underlying databases and the parameters set. Finally, the gene sets associated with CTEPH RVF may not be representative of all conditions resulting in pulmonary hypertension and RVF.

In conclusion, this study identifies multiple, functionally connected genes that are consistently differentially regulated in chronic RVF of rats and humans. Altough descriptive in nature, these datasets and their analyses provide a rich resource for future functional studies and the discovery of novel combinations of biomarkers that will serve to demarcate the transition from compensated RVH to decompensated RVF.

## Methods

### Animal studies

The experimental protocols were approved by the regional authorities and ethics committees for animal research (RP Giessen, registered under number G14-2017) and conformed to the guidelines of Directive 2010/63/EU of the European Parliament on the protection of animals and on ARRIVE 2.0 guidelines.

### Human studies

All patients provided written informed consent for their participation in the study, and approval of the institutional review board of the Justus Liebig University of Giessen (AZ 44/14, 144/11, 145/11, 146/11 and 199/15) was obtained. The investigation conforms to the principles outlined in the Declaration of Helsinki.

### AOB and PAB models in rats

Ascending AOB and PAB were performed in 7-week-old weanling Wistar × Lewis hybrid rats using non-constricting titanium clips (Weck Horizon, Teleflex Medical GmbH) with inner diameters of 0.6 mm (AOB) or 1.1 mm (PAB). Clip sizes were based on previous investigations for AOB^54^ or on preceding tests (for PAB) and resulted in similar hypertrophy rates by AOB or PAB in male and female rats. Anterolateral thoracotomy under isoflurane anaesthesia in fully ventilated rats was performed as published, with sham-operated animals serving as age-matched controls^55^. In growing-up animals, increasing vasoconstriction resulted in compensatory LV (AOB model) or RV hypertrophy (PAB model) 7 weeks after surgery, followed by the onset of LV (AOB model) or RV (PAB model) failure 22–26 weeks later.

Clinical conditions of animals were monitored by assessing (1) cardiac geometry and function using two-dimensional/M-mode echocardiography according to American Society of Echocardiography criteria (Vevo 2100 system, Fujifilm VisualSonics); (2) clinical signs of HF, including tachypnoea, pleural effusion, enlarged liver and ascites; and (3) recording body and ventricle weights.

Echocardiography was performed 1 week after PAB or AOB to prove correct clip positioning, at 7 weeks and during decompensation at 22 weeks (PAB-F/Sham-F) or 26 weeks (AOB-F/Sham-F) after surgery. These timepoints were chosen based on preceding experiments, in which the time-dependent progression of cardiac hypertrophy and deterioration of RV or LV function was determined.

Compensatory and decompensated states were defined based on echocardiographic analyses, hemodynamics (RV or LV pressures and contractility) and clinical parameters (plasma BNP and lung and liver weight) compared to the corresponding age-matched sham controls.

Compensatory RV hypertrophy was defined as (1) significantly increased RV systolic pressure and RV contractility (RV dp/dt maximum); (2) a 30–40% decrease in FAC or TAPSE; and (3) unchanged liver weight and RVEDP. At RV decompensation, liver weight and RVEDP increased, RV developed pressure (mmHg g^−1^ RV weight) decreased and FAC or TAPSE further went down by 60–70%.

Compensatory LV hypertrophy was characterized by (1) increased LV systolic pressure, LV contractility (LV dp/dt maximum) and LV posterior wall thickness and (2) unchanged lung weight and left ventricular end-diastolic pressure (LVEDP). At LV decompensation, lung weight and LVEDP increased, while EF, FS and LV developed pressure decreased.

No premature deaths occurred in the chronic phase of our study. All animals reached this stage and were included.

### Isolation of adult rat cardiomyocytes and RNA

Adult rat ventricular cardiomyocytes were isolated from rats as described previously^56^. In brief, hearts were excised under deep anesthesia, transferred to ice-cold saline and mounted on the cannula of a Langendorff perfusion system. Hearts were perfused first in a non-recirculating manner with 35 ml of calcium-free perfusion buffer followed by 20–25 min in recirculating buffer supplemented with collagenase and 25 µM CaCl_2_. RV and LV were minced separately and left for another 5 min. The resulting cell suspension was filtered through 200-µm nylon meshes, and cell pellets of RV and LV cardiomyocytes were collected at 25*g* for 3 min. Cardiomyocyte RNA was isolated by TRIzol reagent (Thermo Fisher Scientific) according to the manufacturer’s instructions. Integrity and quality of RNA were determined using an Agilent 2100 Bioanalyzer. Supernatants containing endothelial cells and fibroblasts were centrifuged at 250*g* for 10 min, and pellets were resuspended in 1 ml of endothelial cell basal medium (PromoCell). Microvascular endothelial cells were purified by magnetic beads (Thermo Fisher Scientific) pre-coated with anti-rat CD31 antibodies (Thermo Fisher Scientific) for 1 h at 4 °C with end-to-end rotation, washed and seeded on 35-mm culture dishes.

The remaining CD31-negative cells were seeded as cardiac fibroblasts in M199 medium supplemented with 10% FCS. Total RNA was isolated from confluent endothelial cells and fibroblasts as described above.

### Hemodynamic measurements

Wistar rats were intubated and anesthetized with 3–5% isoflurane/100% oxygen, placed on homoeothermic plates (AD Instruments) and ventilated at a constant frequency (SAR-830/P, Föhr Medical Instruments GmbH) with anesthesia maintained with 1–2% isoflurane/100% oxygen. 2F or 1.4F Micro-Tip pressure catheters (Millar Instruments) were placed into the LV through the left carotid artery or into the RV through the right jugular vein to measure LV or RV pressures, respectively. PowerLab data acquisition systems (MPVS-Ultra Single Segment Foundation System, AD Instruments) and LabChart 7 were used to collect and analyze data.

### Histological assessment of cardiomyocyte hypertrophy

After fixation in 4% (v/v) formalin, 10-µm transverse cryosections of the RV and the LV were stained with fluorescein isothiocyanate (FITC)-conjugated wheat germ agglutinin (WGA-FITC; Sigma-Aldrich Chemie GmbH; 1:8 in 1 mM Tris pH 7.4) for 1 h at room temperature. Sections were washed twice in PBS, and nuclei were stained with DAPI in VECTASHIELD mounting medium (BIOZOL Diagnostica Vertrieb GmbH) and subjected to digital slide scanning using a morphometric system (Qwin, Leica Microsystems). Transnuclear cross-sectional size of cardiomyocytes^57^ was quantified with ImageJ software (National Institutes of Health).

### Blood samples

Rats starved for 12 h were killed by cervical dislocation, and blood was obtained by aortic puncture. After removal of cellular components by centrifugation, supernatants (serum) were stored at −80 °C. BNP concentration was measured using commercial ELISA (BNP-45 EIA Kit, Phoenix Pharmaceuticals) according to the manufacturer’s instructions. PBMCs were isolated from blood samples with Ficoll density gradient medium in 15-ml SepMate tubes (STEMCELL Technologies). PBMCs were collected by centrifugation at 1,200*g* for 10 min at room temperature and washed in PBS plus 2% FBS, and RNA was isolated by TRIzol reagent.

### In vivo microCT

Quantitative multi-phase cine cardiac images were acquired using a Quantum GX microCT scanner (PerkinElmer) in conjunction with the contrast agent eXIA160XL (Binitio Biomedical) as described previously^58^.

Before imaging, the rat was restrained, and an intravenous catheter pre-filled with heparinized saline solution was introduced into a lateral tail vein. Rats were transferred into induction chambers and anesthetized with 3% isoflurane in oxygen. Animals were placed on a scanner bed, and 1.0–1.5% isoflurane in oxygen was supplied with a nose cone. Continuous electrocardiographic monitoring was carried out by electrodes placed on right and left front paws. Then, 5 µl g^−1^ of body weight contrast agent was infused at 0.3 ml min^−1^ using an infusion pump.

The scanner bed was translated longitudinally to align the animal chest within the center of the field of view, and images were acquired by the scanner’s complementary metal–oxide–semiconductor X-ray flat-panel detector with an X-ray tube voltage of 90 kV and current of 80 μA.

MicroCT data were collected in list mode over a single complete gantry rotation with a rotation time of 4 min to collect 14,688 frames in total. Thereafter, rats were allowed to recover fully from anesthesia under supervision. Projection images acquired at 16-ms temporal resolution were transferred to an analysis workstation, and retrospectively reconstructed volumes were loaded into Analyze 12 software (AnalyzeDirect). Short-axial image reformation was performed before LV and RV endocardial contour delineation, and the individual LV and RV volumes were calculated for all reconstructions. Fourier fitting was applied to reduce the fluctuation of the first derivative (MATLAB, MathWorks).

### Immunofluorescence analysis of isolated rat cardiomyocyte and non-cardiomyocyte fractions

Freshly isolated cardiomyocytes were seeded on laminin-treated glass-bottom dishes (CELLview cell culture dish, Greiner Bio-One) and fixed with 4% paraformaldehyde at 37 °C for 20 min. Cells were permeabilized with 2% BSA, 0.2% Triton X-100 in PBS at room temperature for 1 h. Cardiomyocyte or fibroblast samples were stained with antibodies against cardiac troponin T antibody (Thermo Fisher Scientific, MA5-12960, 1:200) or vimentin (Cell Signaling Technology, 5741, 1:100) at 4 °C overnight followed by anti-mouse Alexa Fluor 488 and anti-rabbit Alexa Fluor 555 secondary antibodies, respectively. Endothelial cells were stained with Griffonia Simplicifolia Lectin I (Isolectin B4, DyLight 594, Vector Laboratories, 1:100) at room temperature for 1 h. Nuclei were stained with DAPI, and photomicrographs were taken on a BZ X800 fluorescence microscope (Keyence).

### Western blotting

Proteins were extacted from isolated cardiomyocytes in 50 mmol L^−1^ Tris HCl, 150 mmol L^−1^ NaCl, 5 mmol L^−1^ EDTA, 1% SDS and 1% sodiumdeoxycholate, including protease and phosphatase inhibitor cocktails (Sigma-Aldrich Chemie GmbH). Then, 25 µg of proteins was separated by SDS-PAGE and transferred to nitrocellulose, and membranes were hybridized with antibodies against Penk (Aviva Systems Biology, OAAN01715, 1:1,000) or α-tubulin (Cell Signaling Technology, 2144, 1:1,000) in 2.5% non-fat dry milk in TBS-T. After incubation with peroxidase-conjugated secondary antibody, antigens were detected by enhanced chemiluminescence using a Fusion FX7 imaging system (Peqlab Biotechnologie GmbH).

### Assessment of cardiomyocyte cell shortening

Two biphasic electrical, opposingly oriented rectangular 50-V stimuli of 5-ms duration were used to stimulate cardiomyocytes via two AgCl electrodes at a frequency of 2 Hz^59^. Four signals per cell were averaged to assess the contractile responsiveness of any given cell. Cells were kept in M199 medium with a calcium concentration of 1.25 mM, and cell lengths were measured at a rate of 500 Hz via a line camera. For ΔL/L (%) values, the shortening amplitude (ΔL) was calculated relative to the diastolic cell length (L). Furthermore, maximal contraction and relaxation velocity (µm s^−1^) was analyzed.

### Analysis of rat heart fibrosis

Tissue sections from frozen heart samples of LV or RV were stained with a Picro-Sirius Red Stain Kit (Abcam, ab150681).

### RNA isolation from whole heart tissue

Tissues from isolated ventricles, washed with ice-cold PBS and stored in liquid nitrogen were cut into small pieces and homogenized (rough surface glass douncer, 2 ml, Teflon pestle, B. Braun Melsungen) in 1 ml of TRIzol. After a second homogenization step in a fresh tube using a plastic pestle, total RNA was isolated as above, and integrity and quality were determined using an Agilent 2100 Bioanalyzer system.

### RT–qPCR

To analyze mRNA expression, 0.5 − 1 µg of rDNAse-treated TRIzol-purified total RNA, cleaned up by column purification (Macherey-Nagel Nucleospin RNA Isolation Kit), was reverse transcribed into cDNA using RevertAid Reverse Transcriptase (Thermo Fisher Scientific, EP0441) in a total volume of 20 µl. Then, 1 µl or 2 µl of this reaction mixture was amplified by Fast SYBR Green PCR Master Mix (Applied Biosystems/Thermo Fisher Scientific) using the primer listed in Supplementary Table 9 (as described^60^.

### Cardiomyocyte RNA-seq

Cardiomyocytes were isolated from RVs and LVs of 41 animals, resulting in 82 RNA-seq datasets (Fig. 1a). Ribosomal RNA depletion of total RNA isolated from cardiomyocytes, library preparation and deep sequencing were performed by Novogene on the Illumina NovaSeq platform (150-bp paired-end setup). Raw data were processed using Nextflow^61^ and the nf-core/RNA-seq pipeline (version 3.2)^62^. Sequence reads were trimmed by Trim Galore (version 0.6.6) and mapped to the rat reference genome Rnor6.0 with UCSC feature annotations using STAR (version 2.6.1d)^63^, and read counts were extracted with FeatureCounts (Rsubread version 2.2.6), disabling multiple overlaps^64^. Over 90% of all reads present in the 82 samples were mapped to genomic features of the rat genome, and more than 80% were uniquely mapped and represented by at least 25 million reads per sample. Differential expression was analyzed by DESeq2 (version 1.28.1) using the independent filtering option and beta_prior LFC-shrinkage^65^ and the Galaxy^66^ platform of the Justus Liebig University of Giessen.

### Whole heart tissue RNA-seq

RV, septum and LV tissues were separated from hearts of 33 animals, resulting in 99 RNA-seq datasets in total (Extended Data Fig. 7a). Polyadenylated RNA was purified from 500 ng of total RNA using the NEBNext poly(A) mRNA magnetic isolation module kit in 34-µl reaction volumes with adapted amounts of beads and buffers. RNA-seq libraries were prepared using NEBNext Ultra II directional RNA library prep kit for Illumina, and the manufacturer’s protocol was adapted to reduce reaction volumes to 1/3 (7.7 µl of master mix added to RNA-loaded beads). Libraries were quality controlled using Agilent Bioanalyzer high-sensitivity DNA chips, and DNA concentrations were determined using Qubit Analyzer with Qubit high-sensitivity DNA reagent (Thermo Fisher Scientific). Pooled libraries were prepared using SPRIselect beads (Beckman Coulter) and sequenced on an Illumina NextSeq 500 platform (75-bp single-end setup). Raw data were processed using the Nextflow^61^ and nf-core/RNA-seq pipeline, version 1.5 (ref. ^62^). Sequence reads were trimmed by Trim Galore (version 0.6.4) and mapped to the rat reference genome Rnor6.0 with UCSC feature annotations using STAR (version 2.6.1d)^63^, and read counts were extracted with FeatureCounts (Rsubread version 2.2.6), disabling multiple overlaps^64^. For all 99 samples, over 95% of reads were mapped to Rnor6.0 features, and more than 75% were uniquely mapped and represented by at least 27 million per sample. Differential expression was analyzed by DESeq2 (version 1.28.1) as described above.

### Classification of the CTEPH cohort

The present prospective cohort study included a total of 73 patients (all-comers) with a final diagnosis of CTEPH who were treated by PEA at the Kerckhoff Heart and Thorax Center between 2016 and 2020. Data on demographics, symptoms and comorbidities were collected for all patients. Baseline characteristics concerning LV and RV dimensions, function and pressure gradients were further evaluated by transthoracic echocardiography and right heart catheterization (RHC). Seventy-one patients, for which biopsy samples at the prePEA stage were available, were grouped according to 1-year mortality risk by applying a modified version of the ESC guidelines risk stratification model^21^, using the following criteria: low risk (<5%): cardiac index (CI) ≥ 2.0 L/min/m^2^, NT-proBNP < 300 ng/L, TAPSE/sPAP > 0.32 mm/mmHg; intermediate risk (5–20%): NT-proBNP: 300–1,100 ng/L, TAPSE/sPAP: 19–32 mm/mmHg; and high risk (>20%): CI < 2.0 L/min/m^2^, NT-proBNP > 1,100 ng/L, TAPSE/sPAP < 0.19 mm/mmHg. Additionally, in the low or high groups, one parameter was allowed to differ, but two out of three had to meet the pre-defined values. Otherwise, patients were assigned to the intermediate group.

### RNA isolation and RNA-seq analysis of samples from patients with CTEPH

Biopsies of the free RV wall from 71 patients were collected at BL (prePEA) during PEA. In 24 patients, RV myocardial biopsies were obtained during RHC 12 months after PEA (FU, postPEA). In this case, to account for technical and safety aspects, the specimens were taken from the interventricular septum. RNA samples prePEA and postPEA were available for 22 patients, while from two additional patients only postPEA samples were available. Isolation of total RNA from both types of heart tissues, comprising 95 samples in total, was performed with Qiagen miRNeasy Micro Kit and Covaris Cryo-Prep homogenization. All tissue specimens were processed by blinded staffs. In total, 100 ng to 1 µg of total RNA was used for HI Mammalian whole transcriptome preparation (Takara Bio), and sequencing was performed on a NextSeq 2000 instrument with 72-bp single-end setup. Trimmomatic (version 0.39) was employed to trim reads after a quality drop below a mean of Q15 in a window of five nucleotides and keeping only filtered reads longer than 15 nucleotides^67^. Reads were aligned versus Ensembl human genome version hg38 (Ensembl release 104) with STAR 2.7.10a^63^. Aligned reads were filtered to remove duplicates, multi-mapping events and ribosomal or mitochondrial reads using Picard version 2.27.1 (Picard Toolkit 2019, Broad Institute, GitHub repository, https://broadinstitute.github.io/picard/, RRID:SCR_006525). Gene counts were established with featureCounts version 2.0.2 by aggregating reads overlapping exons on the correct strand, excluding those overlapping multiple genes^64^. The raw count matrix was normalized with DESeq2 version 1.30.1 (ref. ^65^). Contrasts were created with DESeq2 based on the raw count matrix. Genes were classified as significantly differentially expressed at average count greater than 5, multiple testing adjusted *P* < 0.05 and −0.585 < log_2_FC > 0.585. The Ensembl annotation was enriched with UniProt data.

### smRNA-FISH

Heart ventricles were washed with ice-cold PBS and frozen in liquid nitrogen. Then, 7-µm traverse tissue sections, prepared at the two chamber ventricle level (omitting valves), were analyzed by smRNA-FISH using RNAscope Fluorescent Multiplex Assay (Advanced Cell Diagnostics, Bio-Techne) according to the manufacturer’s protocol for fresh frozen tissue. In brief, tissue was permeabilized by dehydration and protease treatment, followed by hybridization of the selected RNA probes for *Nppa*, *Nppb*, *Penk*, *Acta1*, *Ankrd23* and *Tceal7* and amplification of the signals. Negative control probes for each channel (C1 and C2) were directed against a bacterial RNA and provided by ACD Bio-Techne. Microscopy analysis was performed using a Leica THUNDER imager (Leica Microsystems CMS GmbH) and Leica Application Suite X (version 3.7.4.23463), using pre-defined exposure times (Phase contrast: 55 milliseconds (ms), DAPI: 80 ms, C1 (555nanometer (nm)): 100 ms, C2 (635 nm): 700 ms). After bright-field preview scanning, preserved morphologies of whole heart sections and of fluorescence signals of single tiles were validated. Tile selections for region-specific quantification were based on bright-field scanning. Spots and nuclei (×200 magnification) were detected and quantified by Icy software (version 2.4.2.0) (https://icy.bioimageanalysis.org) using the following settings. For nuclei, the thresholder value was adjusted to 15 *k*-means classes and HK-means to intensity classes of 12 and a minimum object size of 20 pixels. For spot detection, the object size was defined as 1 pixel with a sensitivity of 30. Spot numbers of each image were divided by the numbers of detected nuclei and normalized using a background factor derived from dividing mean background level by the background of the individual experiment. Imaging of all tiles across the entire section resulted in whole section overview images using Leica Application Suite X.

### Proteomic analyses of rat heart tissues

Rat LV and RV tissue samples from 56 animals (eight per group), resulting in 112 samples, were placed in Lysing Matrix D tubes containing 4 M GuHCl buffer (4 M GuHCl, 25 mM EDTA, 50 mM sodium acetate, pH 5.8, with protease/phosphatase inhibitors) and homogenized using Lysing Matrix D in a FastPrep24 tissue homogenizer at 6,500 r.p.m. for 15 s, twice. The supernatant was sonicated twice for 10 s before centrifugation for 20 min at 16,000*g* at 4 °C. Protein concentration was measured with a Pierce BCA Protein Assay Kit (Thermo Fisher Scientific). For each sample, 50 µg of protein was precipitated using 10× volume of absolute ethanol and incubated at −20 °C overnight. The samples were centrifuged for 30 min at 16,000*g* at 4 °C, and the pellets was dried using a SpeedVac (Thermo Fisher Scientific). Protein pellets were dissolved and denatured by 6 M urea/2 M thiourea, reduced by 10 mM dithiothreitol for 1 h at 37 °C and alkylated using 50 mM iodoacetamide for 1 h at room temperature in the dark. The samples were precipitated by using 1 ml of pre-chilled acetone and incubated at −20 °C overnight. The samples were centrifuged for 30 min at 16,000*g* at 4 °C, and the supernatant was discarded. The protein pellet was dried and resuspended in 0.1 M triethylammonium bicarbonate (TEAB, pH 8.5). Proteins were digested by Trypsin/LysC (Promega, enzyme:protein ratio = 1:25) overnight at 37 °C with shaking. The digestion was stopped by adding 1% trifluoroacetic acid (TFA), and peptides were cleaned using C18 cartridges on an AssayMAP Bravo robot (Agilent). After SpeedVac, the dried peptides were resuspended in 0.1 M TEAB to obtain a concentration of 1 μg μl^−1^. A pooled sample was made by mixing the same amount of each sample. Then, 20 μg of samples along with pooled samples were labeled with TMTpro 18plex reagents (Thermo Fisher Scientific) according to the manufacturer’s instructions. After quenching with 5% hydroxylamine, one pooled sample and 16 samples labeled with different TMTpro tags were mixed together, dried and resuspended in 0.1% triethylamine. Peptide fractionation was performed using high-pH reversed-phase high-pressure liquid chromatography (HPLC) on a ZORBAX Extend 300 C18 column (Agilent, 4.6 × 150 mm), and 16 fractions were collected for 300 µg of each mixed TMT-labeled sample. The fractionated samples were dried using a SpeedVac and resuspended in 37.5 μl of LC solution (2% acetonitrile and 0.05% trifluoroacetic acid in liquid chromatography–mass spectrometry (LC–MS)-grade water). For LC–MS/MS analysis, 5 μl of each fractionated sample was injected and separated by reversed-phase nano-flow HPLC (Thermo Fisher Scientific, UltiMate 3000 RSLCnano) on a 50-cm EASY-SPRAY C18 column (Thermo Fisher Scientific) over a 2-h gradient at a flow rate of 0.25 μl min^−1^ as follows: 0–1 min, 1% B; 1–6 min, 1–6% B; 6–40 min, 6–18% B; 40–70 min, 18–35% B; 70–80 min, 35–45% B; 80–81 min, 45–99% B; 81–89.8 min, 99% B; 90–120 min, 1% B, where A = 0.1% formic acid in LC–MS-grade water and B = 80% acetonitrile and 0.1% formic acid in LC–MS-grade water. The separated peptides were directly injected into an Orbitrap Fusion Lumos Tribrid mass spectrometer (Thermo Fisher Scientific) interfaced with FAIMS Pro Duo in front. In the FAIMS, three compensation voltages (CV = −40 V, −55 V and −70 V) were used alternatively to further separate the peptides in gas phase. The peptides were analyzed using a data-dependent MS2 method with Full MS scan range (400–1,600 *m*/*z* and resolution 120,000) in Orbitrap, and data-dependent MS2 scans were performed on the most abundant precursors from Full MS scan using higher-energy collisional dissociation (HCD) and detected in Orbitrap with resolution 50,000 and isolation windows of 0.7 *m*/*z*. The TMTpro reporter ions were also generated during the HCD fragmentation and recorded in the same MS2 spectra. Cycle time was set at 4.5 s (1.5 s for each CV), and dynamic exclusion was enabled. Data analysis was performed using Proteome Discoverer version 2.4.1.15 (Thermo Fisher Scientific) with Mascot 2.6.0 (Matrix Sciences). The following parameters were used: a combined RAT reference proteome database complemented with MOUSE and HUMAN UniProt/SwissProt database protein entries (release 2022_01, 21589 + 17107 + 20376 protein entries) was used because the rat SwissProt database is not as complete as mouse and human; trypsin was used as enzyme, and two missed cleavages were allowed; precursor mass tolerance was set at 10 ppm, and fragment mass tolerance was 20 mmu; TMTpro 18plex tag on lysine and peptide N-terminal, carbamidomethylation on cysteine were set as static modifications; and oxidation on methionine, lysine and proline was set as variable modifications. Afterwards, identified protein lists were manually checked, and, for proteins identified in different species, rat proteins were kept as first choice, followed by mouse proteins and human proteins. In total, 4,149 protein IDs were identified. TMT reporter ion signal was used as peptide quantitative value and summed up to represent the protein abundances. The data were normalized using the total peptide abundance and then further scaled using the pooled sample abundance as control, correcting for technical variation between injections and TMT groups. The scaled abundance values were exported into Excel and used for further analysis.

Scaled, normalized LC–MS/MS data derived from TMT labeling were log_2_ transformed and width normalized using Perseus software, version 1.6.15.0 (ref. ^68^). The eight biological replicates were assigned to one analysis group per condition. The data matrix was reduced to 3,768 IDs based on 75% (that is, six out of eight) valid values in at least one group. For separation of sex-specific proteomes, condition groups were split according to sex, resulting in 4,039 IDs with 75% valid values. Significantly differentially expressed proteins (DEPs) between groups were identified based on *t*-tests and a –log_10_ *P* ≥ 1.3 using Perseus functionalities. Subsequent filtering steps and heatmap representations were performed in Excel 2016 according to the filtering criteria and thresholds described in the figure legends.

### Bioinformatic analysis and data visualization

RNA and protein samples were collected at different timepoints and stored frozen at −80 °C. RNA-seq or MS runs were performed together, respectively. z-scores were based on the formula z = (x − μ) / σ, where x is the gene expression value, µ is the average mRNA expression per group and σ is the standard deviation. All graphs and statistical tests of non-omics data (two-sided *t*-tests and ANOVA, correlation analyses) were performed using GraphPad Prism version 9.4.1 (GraphPad Software) or Excel 2016. Venn diagrams were generated online using a Venn diagram tool (http://bioinformatics.psb.ugent.be/webtools/Venn/). For heatmap illustrations, z-score calculations or cluster analyses, Excel 2016 or the web tool Morpheus (https://software.broadinstitute.org/morpheus) were used. Gene sets were segregated by *k*-means clustering with ‘one minus pearson correlation’ based on row values. PPI network analyses were based on the newest version of the STRING database (https://string-db.org/)^69^ and visualized using Cytoscape version 3.9.1 using the integrated STRING functionalities and all ontology databases of the STRING application^70^.

Pathway enrichment analyses were performed online by Metascape (https://metascape.org/) with the following default settings. For individual or multiple gene lists, KEGG Pathway, GO Biological Processes, Reactome Gene Sets, Canonical Pathways, CORUM, WikiPathways and PANTHER pathways were used as ontology sources. All genes in the genome were used as the enrichment background. Terms with *P* < 0.01, a minimum count of 3 and an enrichment factor (ratio between observed counts and counts expected by chance) greater than 1.5 were collected and clustered by their membership similarities. *P* values were calculated based on the cumulative hypergeometric distribution, and *q* values were calculated using the Benjamini–Hochberg procedure to account for multiple testings. Kappa scores were used as the similarity metric for hierarchical clustering of enriched terms, and sub-trees with a similarity of greater than 0.3 were considered a cluster. The most statistically significant term within a cluster was chosen to represent the cluster^71^. In case of multiple input gene lists, these were merged into one list called ‘_FINAL’. If terms were enriched in several individual gene lists and/or in the _FINAL gene list, the best *P* value was chosen as the final *P* value.^71^.

Enrichment analyses for TF-regulated gene sets were performed online using using the network TF target functionality of WebGestalt (https://www.webgestalt.org/)^72^ and the TF gene sets of the Molecular Signatures Database (MSigDB) (https://www.gsea-msigdb.org/gsea/msigdb)^73^ with the following settings. Enrichment method: ORA; organism: rnorvegicus; enrichment categories: network_Transcription_Factor_target; ID type: genesymbol; reference list: all mapped entrezgene IDs from the selected platform genome. Parameters for the enrichment analysis: minimum number of IDs in the category: 5; maximum number of IDs in the category: 2,000; false discovery rate method: Benjamini–Hochberg; significance level: top 10.

Secretome annotations were based on the published list of 6,943 high-confidence human secreted proteins that were generated from 330,427 human proteins derived from databases of UniProt, Ensembl, AceView and RefSeq (SPRomeDB, www.unimd.org/SPRomeDB). In total, 6,267 of 6,943 (90.3%) of these proteins have supporting evidence from a large amount of MS and RNA-seq data as published in ref. ^18^.

### Re-analysis of scRNA-seq or snRNA-seq datasets

For processing of published single-cell and single-nucleus transcriptomic data, snRNA-seq data from LVs of hearts from donors and patients with DCM or HCM were downloaded from the Single Cell Portal of the Broad Institute (https://singlecell.broadinstitute.org/single_cell/study/SCP1303/)^22^. In total, processed information for 592,689 cells was obtained in h5ad format (downloaded file: human_dcm_hcm_scportal_03.17.2022.h5ad). The file was converted into h5seurat format and subsequently loaded into the R environment (https://www.R-project.org/) using Seurat version 5.0.0^74^. The same procedures were followed to re-use scRNA-seq/snRNA-seq data from the human heart atlas, version 2, comprising 704,296 individual cells representing 12 cardiac cell types (https://www.heartcellatlas.org/index.html)^17,23^. Differential gene expression between disease conditions and control donors was calculated in cardiomyocytes (I, II, III or the union of all subtypes) using a pseudo-bulk approach as outlined in https://hbctraining.github.io/scRNA-seq_online/lessons/06a_integration_harmony.html. Data were converted into a single-cell experiment object, and subsets of cardiomyocyte compartments were selected (based on cell_type_leiden0.6 column)^75^. Data were split according to samples and cell types, and, subsequently, DESeq2 version 1.42.0 was used to identify DEGs for contrasts HCM versus non-failing (NF) and DCM versus NF^65^. Subsequently, genes with a base mean of fewer than five reads were removed, and GSEAs were performed across five different gene signatures: RHF core signature (113), hLHF+Rat PAB (57), hLHF+hCTEPH+Rat PAB (35), hCTEPH+ Rat PAB (21) and Rat PAB (111). GSEA was performed using the GSEA function of the clusterProfiler package based on the Wald test statistic derived from the DESeq2 analysis^76^. Furthermore, the h5ad file was loaded into a CELLxGENE instance extended with the functionality of the VIP plug-in, which was used to generate Fig. 8c,f and Extended Data Figs. 3d, 4b, 8 and 9 (refs. ^77,78^). The R code for the pseudo-bulk analysis of differential gene expression in cardiomyocytes from published scRNA-seq/snRNA-seq data and subsequent GSEA analysis of gene signatures is accessible at 10.5281/zenodo.10973971.

### Reporting summary

Further information on research design is available in the Nature Portfolio Reporting Summary linked to this article.

### Supplementary information

Supplementary information Supplementary Figs. 1–6 and STROBE checklist.

Reporting Summary

Supplementary Data 1 Statistical source data for supplementary figures.

Supplementary Table 1 Echocardiographic and clinical characterization of rat HF models.

Supplementary Table 2 Hemodynamic characterization of rat HF models.

Supplementary Table 3 RNA-seq data rat cardiomyocytes.

Supplementary Table 4 RNA-seq data whole rat hearts.

Supplementary Table 5 Proteome data rat heart.

Supplementary Table 6 RNA-seq data human CTEPH patients.

Supplementary Table 7 Proteome data rat heart separated by sex.

Supplementary Table 8 Pseudobulk analysis of human single nucleus RNA-seq data of the heart.

Supplementary Table 9 List of primers for PCR

### Source data

Source Data Fig. 1 Statistical source data.

Source Data Fig. 2 Statistical source data.

Source Data Fig. 3 Statistical source data.

Source Data Fig. 4 Statistical source data.

Source Data Fig. 5 Statistical source data.

Source Data Fig. 6 Statistical source data, unprocessed western blots.

Source Data Fig. 7 Statistical source data.

Source Data Fig. 8 Statistical source data.

Source Data Extended Data Fig./Table 1 Statistical source data.

Source Data Extended Data Fig./Table 2 Statistical source data.

Source Data Extended Data Fig./Table 3 Source data Extended Data Fig./Table 3 statistical source data.

Source Data Extended Data Fig./Table 4 Statistical source data.

Source Data Extended Data Fig./Table 5 Statistical source data.

Source Data Extended Data Fig./Table 6 Statistical source data.

Source Data Extended Data Fig./Table 7 Statistical source data.

Source Data Extended Data Fig./Table 8 Statistical source data.

Source Data Extended Data Fig./Table 10 Statistical source data.

## Data Availability

The rat cardiomyocyte and whole heart RNA-seq datasets of this study have been submitted to the Gene Expression Omnibus (GEO): GSE216263 and GSE216264. The hCTEPH RNA-seq data have also been submitted to the GEO: GSE249697. The mass spectrometry proteomics data have been deposited to the ProteomeXchange Consortium via the PRIDE^79^ partner repository with the dataset identifier PXD047022. The remaining data generated in this study are provided in the Supplementary Information and Source Data sections. Source data are provided with this paper.
