# Supplementary material for: A common gene signature of the right ventricle in failing rat and human hearts
Source: Nat Cardiovasc Res. 2024 Jul 5;3(7):819–40. doi: 10.1038/s44161-024-00485-1 (PMC11358011; doi:10.1038/s44161-024-00485-1)
Supplement: Supplementary file 1 — Supplementary Figs. 1–6 and STROBE checklist. [file 44161_2024_485_MOESM1_ESM.pdf]

nature cardiovascular research

Supplementary information https://doi.org/10.1038/s44161-024-00485-1

A common gene signature of the right ventricle in failing rat and human hearts

In the format provided by the authors and unedited

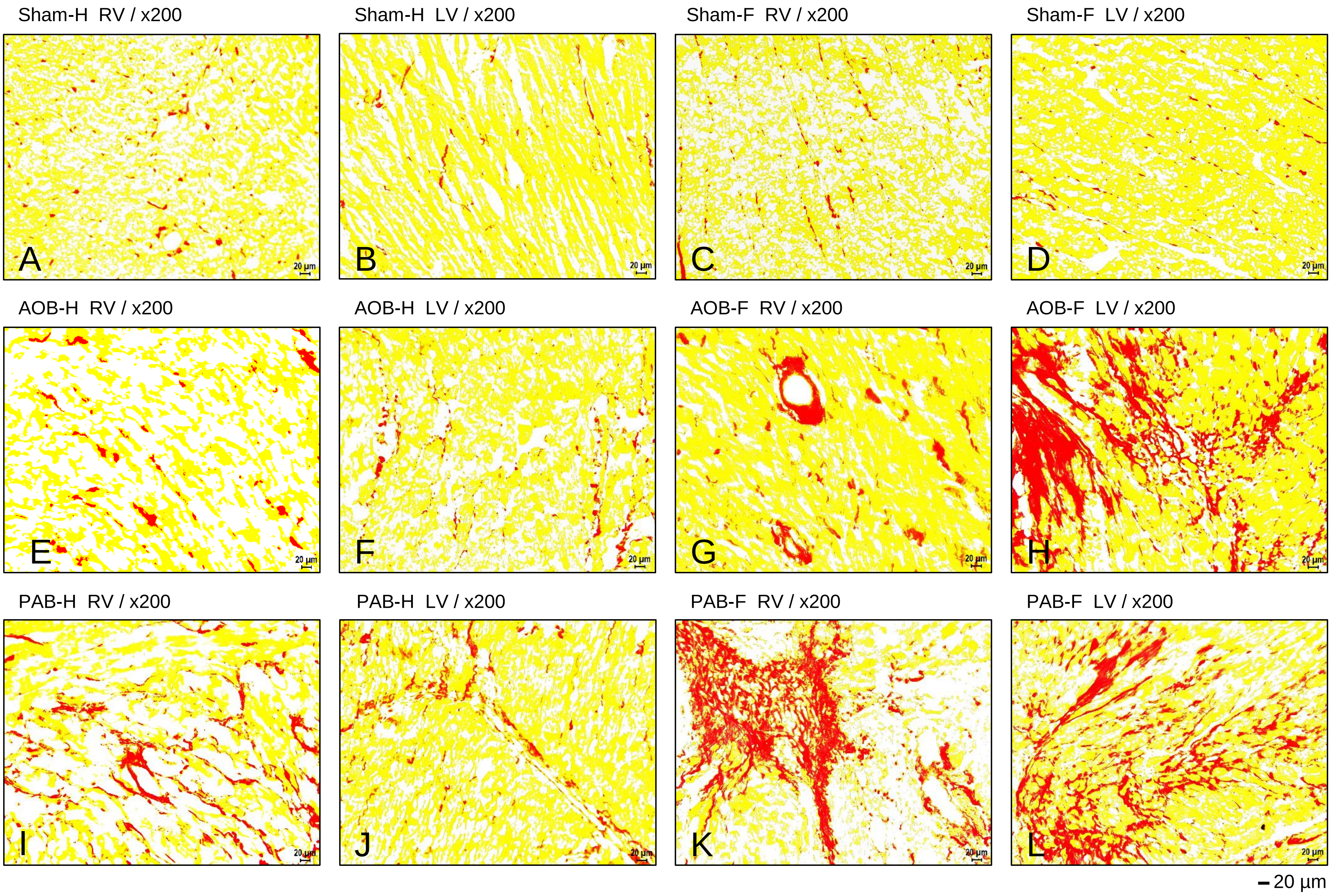

Supplementary Fig. 1. Increased collagen deposition in rat hearts from PAB or AOB models.

The images show collagen staining with Sirius Red of the RV and LV in Sham animals and in rats submitted to AOB or PAB. All photos were taken with magnification x200. All Sham animals at all time points (A-D) show normal collagen quantity and distribution in the RV and the LV, with faint red staining in the perimysium of cardiomyocytes, lacking broad interstitial septa. Animals with AOB or PAB at compensatory hypertrophy (AOB-H and PAB-H) (E, F, I, J) show normal or very mild increase in collagen that is perceivable in the interstitial space / perimysium of the RV and the LV. Similar mild changes can be seen in the RV of animals with AOB at the time point of cardiac decompensation (AOB-F) (G). Strong increase of collagen deposition, with broad interstitial septa and scar-like fibrosis of the myocardium is observed in animals with PAB during cardiac decompensation (PAB-F) within the RV and to a lesser degree in the LV (K, L), as well as in AOB-F animals within the LV (H). The analysis was repeated four times independently with similar results.

A

32 unique TFs

CBFA2T2/3
CEBPB
EBF2
ETS2
FOXA1
FOXL1
FOXO1A
GATA1
GCM1
HSF1
IRF1
ITGAL
JUN
MEF2
MEF2A
MLLT7
NF1
OLF1 / OR5I1
PAX2
PITX2
POU2F1
POU3F2
SMAD3
SREBF1
SRF
STAT5A
STAT5B
TCF8
unknown TF
ZIC3
ZNF238
ZNF384

106 unique TF targets
(of 224 PAB-regulated genes)

Abca5	Ddr2	Ltbp2	Ptk2b
Abhd2	Dgkg	Map4k1	Rxfp1
Acta1	Dlgap1	Mdk	Scn3a
Adcy7	Dpysl3	Mfap5	Scn3b
Aebp1	Ecm1	Mmp2	Sema3b
Aif1l	Epha7	Mrc2	Sema3d
Apba1	Ephx4	Myl4	Serpinf1
Asb4	Epn3	Ncam1	Serping1
Atp1a3	Etv1	Nos1	Sfrp2
Axl	Fap	Nppb	Ssc5d
Bcl11b	Fibin	Npr3	Ston1
Bex1	Fmo2	Nrg1	Sulf1
C2	Fmo3	Numbl	Sv2a
C4b	Fstl1	Olr59	Tceal7
Cacna1g	Fstl3	P2rx1	Tcf21
Camk1d	Fuca1	P3h2	Tgfb2
Camkk1	Fxyd6	Pcdh11x	Tgfb3
Cmklr1	Gabrb2	Pcdh20	Thbs2
Col1a1	Gpnmb	Pcolce	Tmeff2
Col3a1	Hs3st2	Pde1a	Tmem100
Col8a1	Igfbp4	Pdgfra	Tmem229b
Cp	Igfbp6	Pi16	Ttll9
Crlf1	Il25	Plekhh2	Uchl1
Csrp2	Kcnj14	Plod2	Vash2
Ctsk	Lrp1	Prdm1	Vcan
Cxcr4	Lsp1	Prelp	Wdr62
Cyfip2			Xdh

B

Gene	RV Sham-H	RV PAB-H	Log_2 ratio	P value	RV Sham-F	RV PAB-F	Log_2 ratio	P value
Jun	13.2	12.9	-0.3	1.3	13.0	12.6	-0.3	1.7
Srf	11.4	11.2	-0.2	0.9	11.2	10.9	-0.2	1.4
Nf1	11.3	11.3	0.0	0.1	11.3	11.2	-0.1	0.2
Mef2a	11.5	11.0	-0.5	2.8	11.6	10.8	-0.7	6.5
Cebpb	10.3	10.2	-0.1	0.3	10.3	10.1	-0.2	0.4
Stat5b	10.5	10.4	-0.2	1.7	10.5	10.2	-0.3	3.8
Ets2	10.1	10.1	0.0	0.0	10.0	9.9	-0.1	0.2
Smad3	10.1	9.9	-0.3	1.8	9.9	9.6	-0.3	1.7
Hsf1	9.3	9.3	0.0	0.0	9.3	9.3	0.0	0.0
Irf1	9.4	9.5	0.2	0.3	9.1	9.2	0.0	0.1
Srebf1	8.9	8.7	-0.2	0.9	8.8	8.5	-0.3	1.3
Stat5a	8.8	8.5	-0.3	1.6	8.8	8.4	-0.4	2.9
Pou2f1	7.5	7.3	-0.2	1.2	7.5	7.2	-0.3	1.4
Ebf2	6.5	7.3	0.7	2.4	6.7	7.1	0.4	1.0
Itgal	6.6	6.6	-0.1	0.1	6.5	6.7	0.1	0.4
Pitx2	3.5	2.9	-0.5	0.7	3.0	1.7	-0.9	1.9
Foxa1	3.0	2.8	-0.2	0.2	3.4	3.2	-0.1	0.1

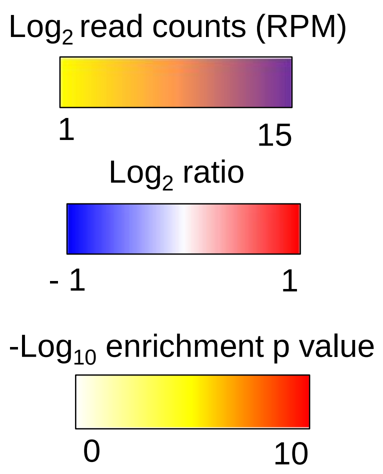

Supplementary Fig. 2. Transcription factors (TFs) regulating PAB target genes.

(A) Top 10 TFs derived from all clusters of PAB-regulated genes (corresponding to 32 unique TFs) and their target genes. (B) Out of 32 TFs, 17 TFs are expressed in the RV of Sham or PAB animals. The heat map shows normalized read counts, fold changes and their significance.

A

30 unique TFs	64 unique TF targets (of 127 AOB-regulated geens)
BACH1	Adam11
E2F1	Angptl4
ETS1	Ankrd23
ETS2	Anxa2
ETV4	Arhgap30
ETV7	Bdkrb2
GFI1	Ccl2
GTF2A1	Ccl20
IRF	Ccl3
KLF12	Ccl7
LEF1	Col11a2
MZF1	Ctss
NFKB	Cxcl1
NKX6-2	Cxcl2
NR1H3	Cybb
PAX4	Dmbt1
PITX2	Eif1a
POU2F1	Fam167a
POU3F2	Fgr
RFX1	Fhl1
RUNX1	Gnai1
SF1	Hck
STAT3	Ifit2
TAF	Il1b
UBP1	Il1rn
unknown TF	Itgb1
USF	Itgb2
VDR	Klhl4
YY1	Lcp1
ZNF384	Ltbp2
	Map1b
	Msn
	Mybpc2
	Myo1f
	Myo3b
	Myo7b
	Myof
	Ncam1
	Nlrp3
	Nos1
	Nppa
	Plau
	Postn
	Prrg4
	Ptgs2
	Rab30
	Reg3b
	S100a4
	Sele
	Selp
	Sema3d
	Serpine1
	Slc1a3
	Slco2a1
	Sorcs1
	Steap2
	Tceal7
	Tgfb2
	Tgm1
	Timp1
	Tmbim1
	Uchl1
	Xpr1
	Ybx2

B

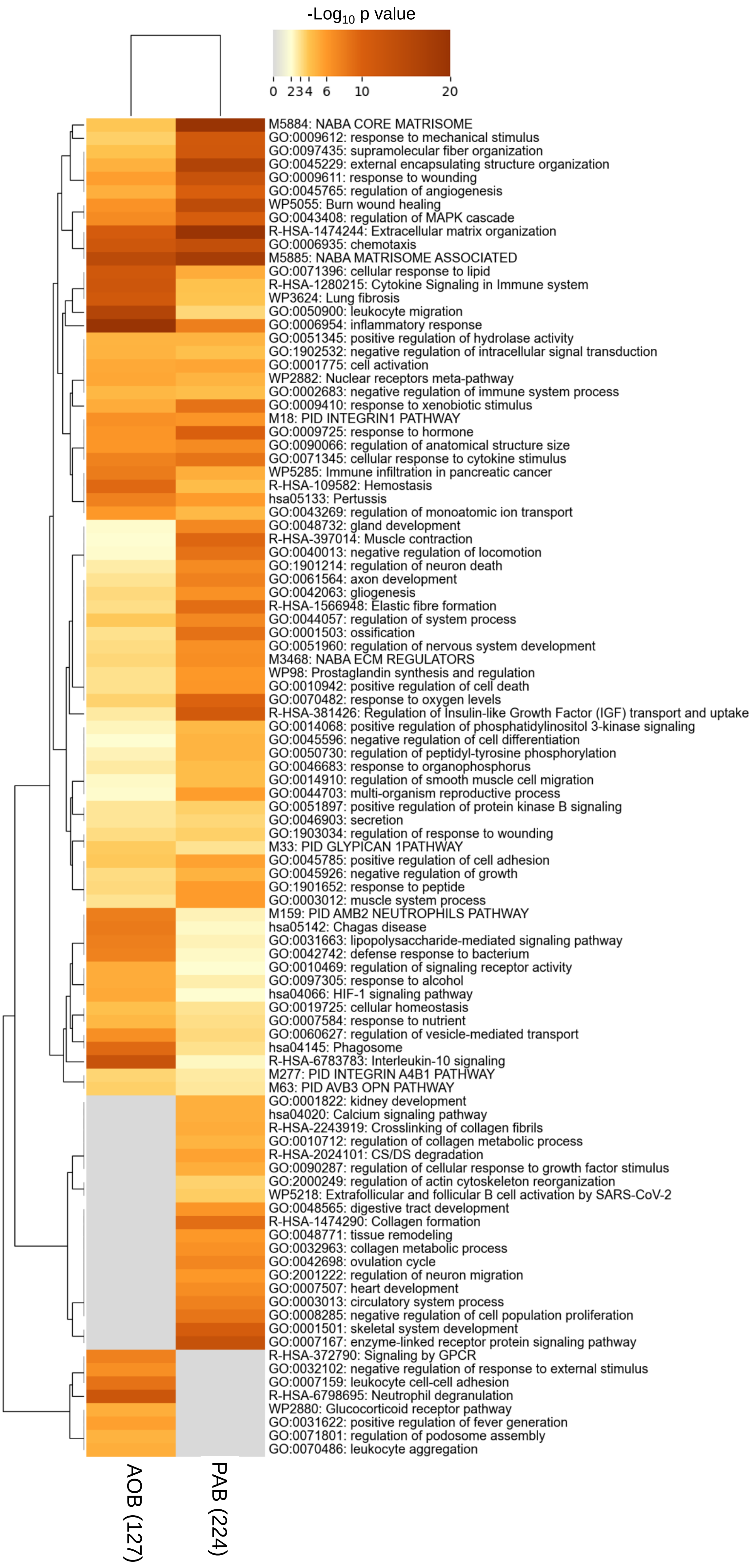

Supplementary Fig. 3. Transcription factors (TFs) regulating AOB target genes and top 100 pathways terms associated with AOB or PAB

(A) Top 10 TFs derived from all clusters of AOB-regulated genes (corresponding to 40 unique TFs) and their target genes. (B) The gene ontologies of all 224 PAB and 127 AOB target genes were determined by pathway overrepresentation analysis. The clustered heat map shows the top 100 enriched pathway terms.

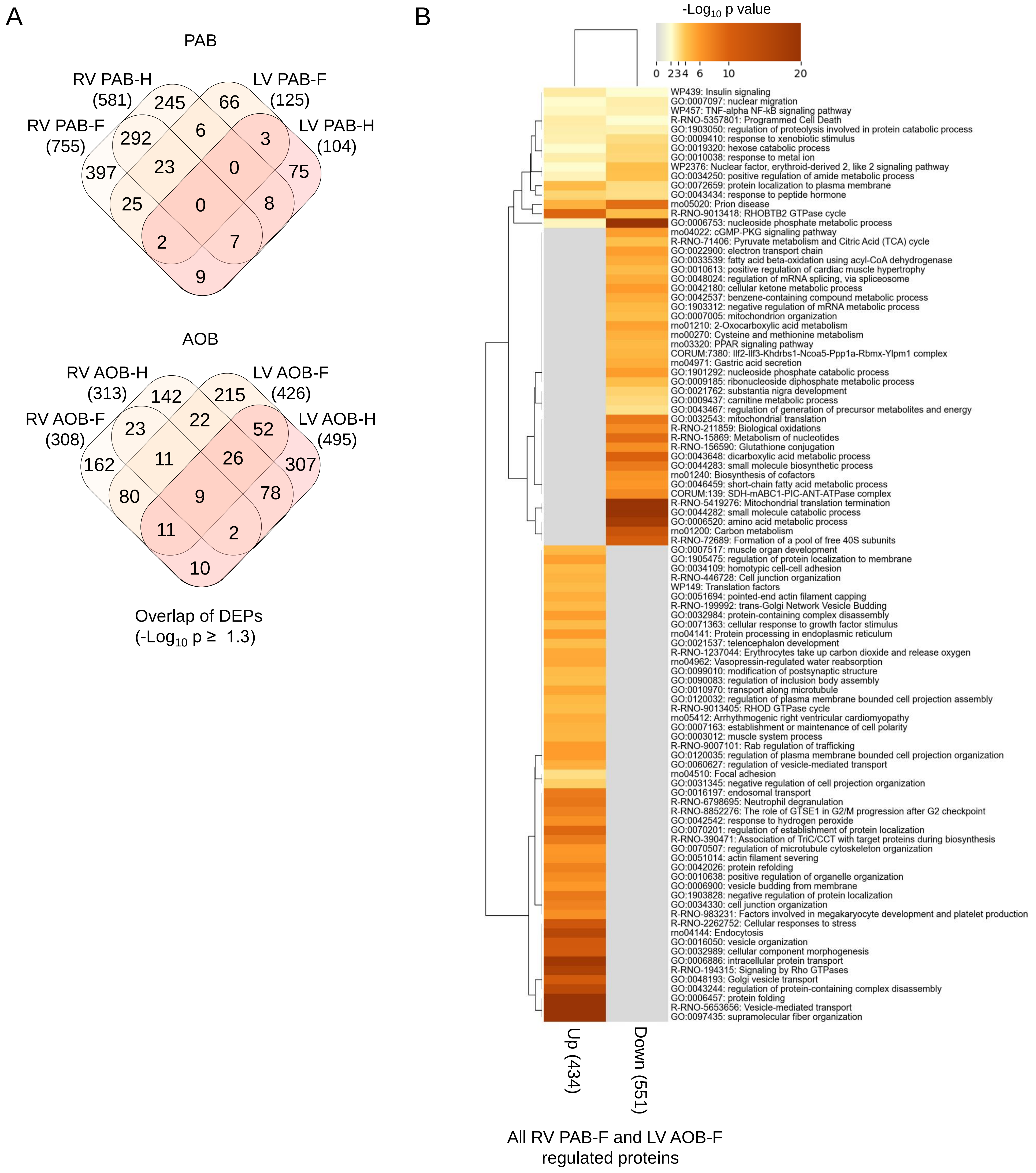

Supplementary Fig. 4. Distinct and overlapping sets of DEPs in PAB and AOB conditions.

(A) Venn diagrams showing the overlap of all DEPs in PAB or AOB conditions. (B) Clustered heatmap of the top 100 enriched pathway terms mapping to all up- or downregulated DEPs in the LV of AOB-F or the RV of PAB-F conditions demonstrating common and unique sets of genes in rat heart failure conditions.

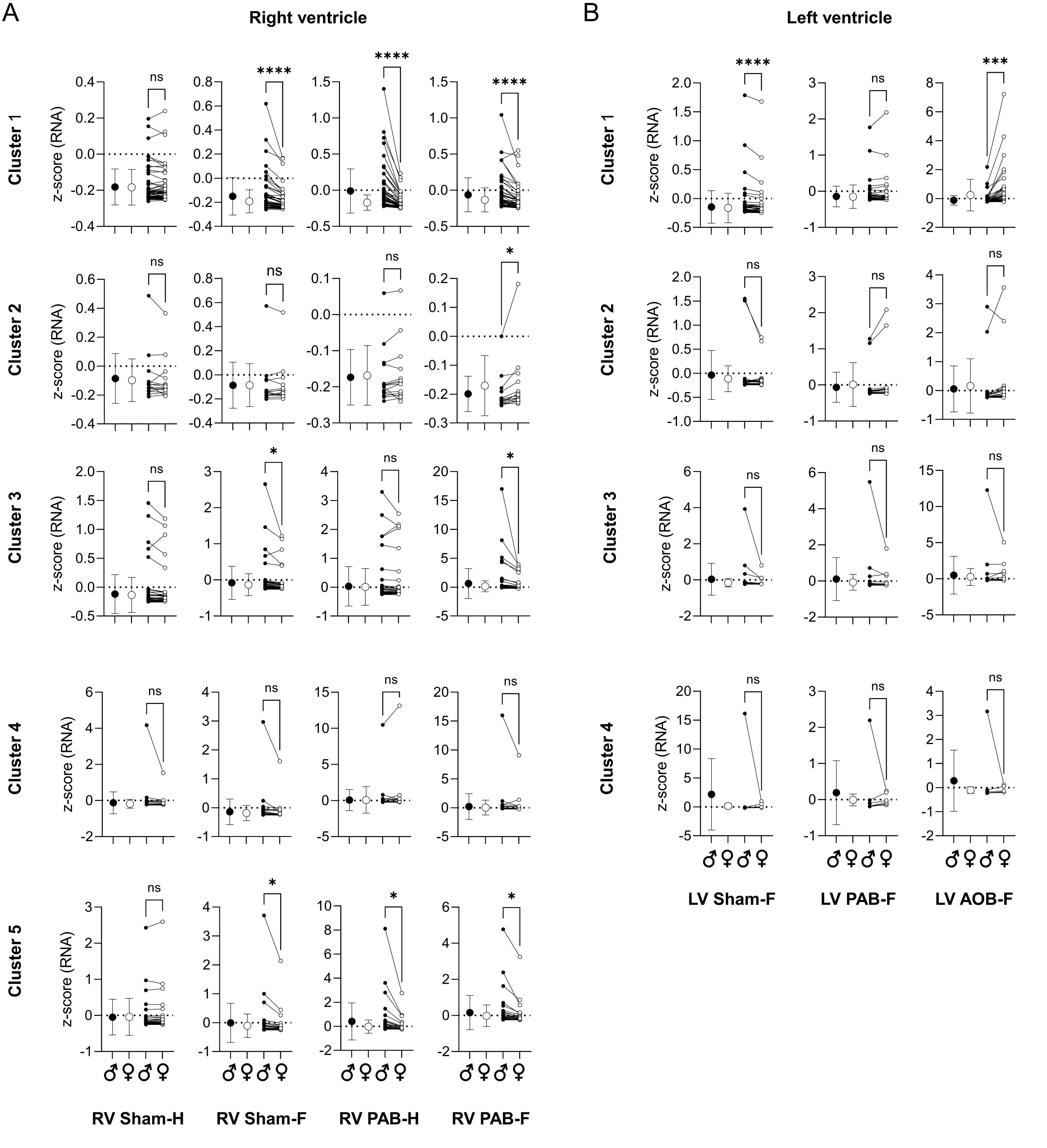

Supplementary Fig. 5. Sex-specific regulation of PAB or AOB-regulated genes.

(A) Z-scored gene expression values of clusters 1-5 of all 224 PAB regulated genes in the RV (see Fig. 3) were separated according to sex. Graphs show mean expression ± s.d. on the left and pairwise aligned expression values for individual genes on the right. Solid black and white symbols mark male and female samples, respectively. Asterisks indicate significance of changes between male and female animals obtained by paired t-tests (*$p \leq 0.05$, **$p \leq 0.01$, ***$p \leq 0.001$, ****$p \leq 0.0001$). (B) Z-scored gene expression values of clusters 1-4 of all 127 AOB-regulated genes in the LV (see Extended Data Fig. 6C) were separated according to sex. Graphs show mean expression ± s.d. on the left and pairwise aligned expression values for individual genes on the right. Solid black and white symbols mark male and female samples, respectively. Asterisks indicate significance of changes between male and female animals obtained by paired t-tests (*$p \leq 0.05$, **$p \leq 0.01$, ***$p \leq 0.001$, ****$p \leq 0.0001$).

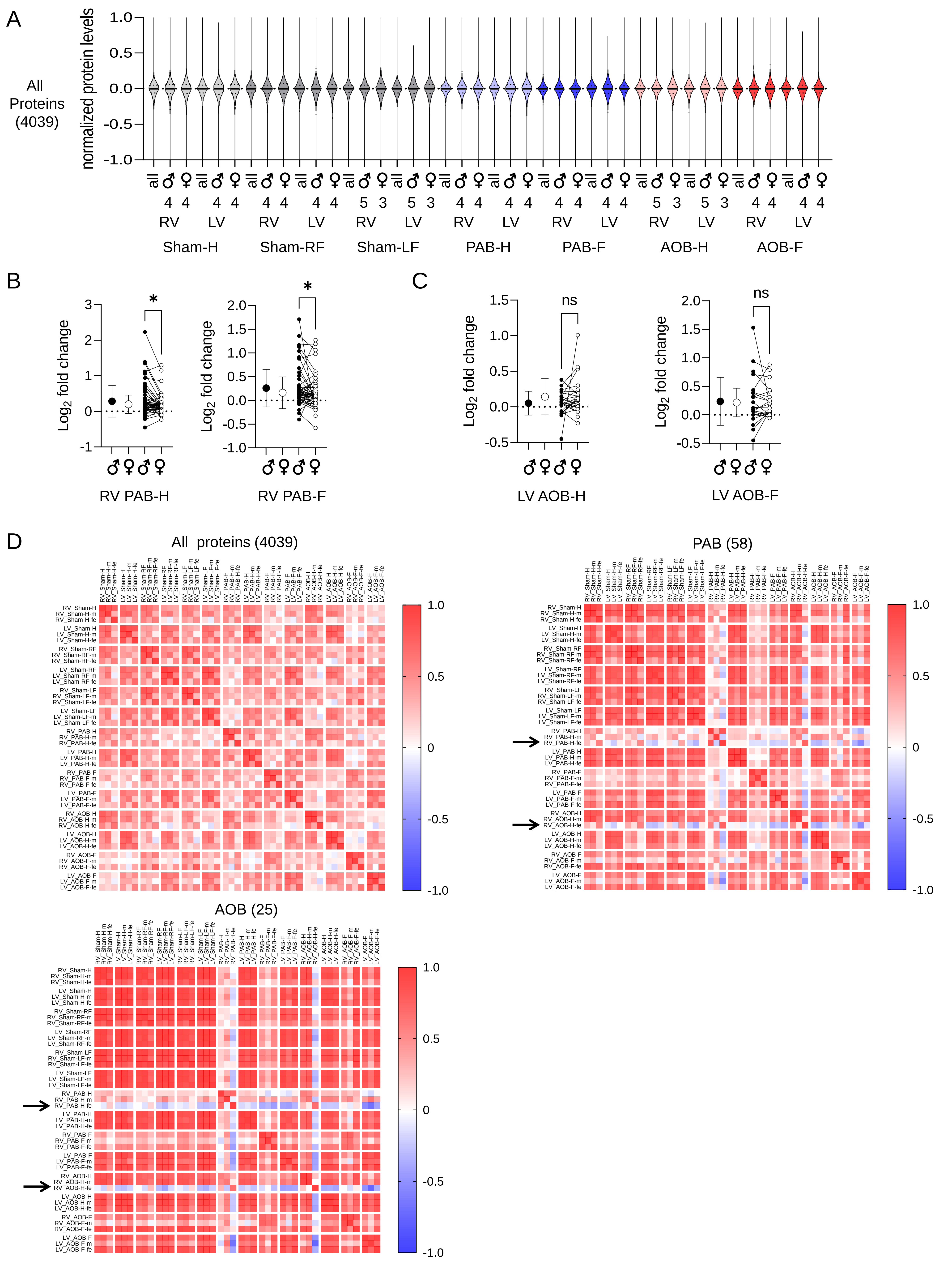
A
All Proteins (4039)
normalized protein levels
1.0
0.5
0.0
-0.5
-1.0
all ♂ ♀
4 4
5 3
RV
LV
Sham-H
Sham-RF
Sham-LF
PAB-H
PAB-F
AOB-H
AOB-F
B
Log2 fold change
*
RV PAB-H
RV PAB-F
C
ns
LV AOB-H
LV AOB-F
D
All proteins (4039)
PAB (58)
AOB (25)
1.0
0.5
0
-0.5
-1.0

Supplementary Fig. 6. Sex-specific regulation of PAB- or AOB-regulated proteins.

(A) Proteomic data were width normalized and split according to sexes resulting in 4039 protein IDs with at least 75% valid values in at least one male or female group. Violin plots show distribution of protein intensity values across all conditions. (B-C) Sex-dependent expression changes of 58 proteins matching to 224 PAB regulated genes in the RV or 25 proteins matching to 127 AOB regulated genes in the LV. Graphs show mean fold change ± s.d. on the left and pairwise aligned Log_2 ratio values for individual DEPs on the right. Solid black or white symbols mark male and female samples, respectively. Asterisks indicate significance of changes between male and female animals obtained by paired t-tests (*$p \leq 0.05$, **$p \leq 0.01$, ***$p \leq 0.001$, ****$p \leq 0.0001$). (D) Correlation matrices comparing the expression of all 4039 proteins or PAB- or AOB-regulated proteins separated by sex across all experimental conditions. Arrows mark differences in correlations of protein expression between male and females in the RV. Colour bars show Pearson r.

STROBE Statement—Checklist of items that should be included in reports of ***cohort studies***

	Item No	Recommendation
**Title and abstract**	1	Indicate the study's design with a commonly used term in the title or the abstract<br>In this study, human samples were used in comparison to the data set obtained in a well characterized animal model. We did not primarily report results of a clinical cohort study.
		Provide in the abstract an informative and balanced summary of what was done and what was found<br>"Intersection of rat PAB-specific gene sets with transcriptome data sets from human patients with chronic thromboembolic pulmonary hypertension (CTEPH) led to the identification of more than 50 genes whose expression levels correlated with the severity of right heart disease, including multiple matrix-regulating and secreted factors. These data define a conserved, differentially regulated genetic network associated with right heart failure in rats and humans."
**Introduction**		
Background/rationale	2	Explain the scientific background and rationale for the investigation being reported<br>"Similar resources and datasets are not available for RHF. In the present study, we performed a systematic investigation of rat models of chronic RHF (pulmonary artery banding, PAB) or LHF (aortic banding, AOB) to uncover the transcriptomic and proteomic changes that occur over months in the failing RV compared with the failing LV. Deep bioinformatics analyses, including comparisons of rat PAB-specific gene sets with transcriptomic data from patients with chronic thromboembolic pulmonary hypertension (CTEPH) before and after pulmonary endarterectomy (pre/postPEA) resulted in the identification of more than 50 genes whose expression levels correlated with the severity of right heart disease in humans. Together, these data define a genetic network representing a first version of a core gene signature of the failing RV that appears to coordinate progressive RHF."
Objectives	3	State specific objectives, including any prespecified hypotheses<br>"…transcriptomic data from patients with chronic thromboembolic pulmonary hypertension (CTEPH) before and after pulmonary endarterectomy (pre/post PEA) resulted in the identification of more than 50 genes whose expression levels correlated with the severity of right heart disease in humans."
**Methods**		
Study design	4	Present key elements of study design early in the paper<br>"The present prospective cohort study included a total number of 73 patients (all-comers) with a final diagnosis of chronic thromboembolic pulmonary hypertension (CTEPH), who were treated by pulmonary endarterectomy (PEA) at the Kerckhoff Heart and Thorax Center between 2016 and 2020. Data on demographics, symptoms and comorbidities were collected for all patients. Baseline characteristics concerning LV and RV dimensions, function and pressure gradients were further evaluated by transthoracic echocardiography and right heart catheterisation. 71 patients analyzed by RNAseq at the prePEA stage were grouped according to 1 year mortality risk by applying a modified version of the European Society of Cardiology (ESC) guidelines risk stratification model 21, using the following criteria. Low risk (< 5%): cardiac index (CI) ≥ 2.0 L / min / m2, NT-proBNP < 300 ng / L, TAPSE / systolic pulmonary arterial pressure (sPAP) > 0.32 mm / mmHg, intermediate risk (5-20%): NT-proBNP: 300-1100 ng / L, TAPSE / sPAP: 19 – 32

		mm / mmHg and high risk (> 20%): CI < 2.0 L / min/m2, NT-proBNP > 1100 ng / L, TAPSE / sPAP < 0.19 mm/mmHg. Additionally, in the low or high groups 1 parameter was allowed to differ, but 2 out of 3 had to meet the pre-defined values. Otherwise, patients were assigned to the intermediate group.”
Setting	5	Describe the setting, locations, and relevant dates, including periods of recruitment, exposure, follow-up, and data collection<br>“The present prospective cohort study included a total number of 73 patients (all-comers) with a final diagnosis of chronic thromboembolic pulmonary hypertension (CTEPH), who were treated by pulmonary endarterectomy (PEA) at the Kerckhoff Heart and Thorax Center between 2016 and 2020.”<br>“Biopsies of the free RV wall from 71 patients were collected at base line (BL, prePEA) during PEA. In 24 patients, RV myocardial biopsies were obtained during right heart catheterization (RHC) 12 months after PEA (follow-up, FU, postPEA). In this case, to account for technical and safety aspects, the specimens were taken from the interventricular septum.”
Participants	6	(*a*) Give the eligibility criteria, and the sources and methods of selection of participants. Describe methods of follow-up<br>See above (Setting)
		(*b*) For matched studies, give matching criteria and number of exposed and unexposed<br>Matched RNA-seq data are available for 22 patients prePEA and postPEA.
Variables	7	Clearly define all outcomes, exposures, predictors, potential confounders, and effect modifiers. Give diagnostic criteria, if applicable<br>“Data on demographics, symptoms and comorbidities were collected for all patients. Baseline characteristics concerning LV and RV dimensions, function and pressure gradients were further evaluated by transthoracic echocardiography and right heart catheterisation. 71 patients, for which biopsy samples at the prePEA stage were available, were grouped according to 1 year mortality risk by applying a modified version of the European Society of Cardiology (ESC) guidelines risk stratification model, using the following criteria. Low risk (< 5%): cardiac index (CI) ≥ 2.0 L / min / m2, NT-proBNP < 300 ng / L, TAPSE / systolic pulmonary arterial pressure (sPAP) > 0.32 mm / mmHg, intermediate risk (5-20%): NT-proBNP: 300-1100 ng / L, TAPSE / sPAP: 19 – 32 mm / mmHg and high risk (> 20%): CI < 2.0 L / min/m2, NT-proBNP > 1100 ng / L, TAPSE / sPAP < 0.19 mm/mmHg. Additionally, in the low or high groups 1 parameter was allowed to differ, but 2 out of 3 had to meet the pre-defined values. Otherwise, patients were assigned to the intermediate group.”
Data sources/ measurement	8*	For each variable of interest, give sources of data and details of methods of assessment (measurement). Describe comparability of assessment methods if there is more than one group<br>“For RNA isolation, biopsies of the free RV wall from 71 patients were collected at base line (BL, prePEA) during PEA. In 24 patients, RV myocardial biopsies were obtained during right heart catheterization (RHC) 12 months after PEA (follow-up, FU, postPEA). In this case, to account for technical and safety aspects, the specimens were taken from the interventricular septum. RNA samples prePEA and postPEA were available for 22 patients, while only postPEA samples were available from two additional patients. Isolation of total RNA from both types of heart tissues, comprising 95 samples in total, was performed with Qiagen miRNeasy micro Kit and Covaris Cryo-Prep homogenization. All tissue specimens were

		processed by blinded staffs. In total, 100 ng to 1 µg of total RNA was used for Hi-Mammalian whole transcriptome preparation (Takara Bio) and sequencing was performed on Nextseq2000 instrument with (72 bp single end set-up). Trimmomatic (v. 0.39) was employed to trim reads after a quality drop below a mean of Q15 in a window of 5 nucleotides and keeping only filtered reads longer than 15 nucleotides. Reads were aligned versus Ensembl human genome version hg38 (Ensembl release 104) with STAR 2.7.10a. Aligned reads were filtered to remove duplicates with Picard 2.27.1 (Picard Toolkit. 2019. Broad Institute, GitHub Repository. https://broadinstitute.github.io/picard/; Broad Institute; RRID:SCR_006525), multi-mapping events and ribosomal or mitochondrial reads. Gene counts were established with featureCounts 2.0.2 by aggregating reads overlapping exons on the correct strand excluding those overlapping multiple genes. The raw count matrix was normalized with DESeq2 version 1.30.1. Contrasts were created with DESeq2 based on the raw count matrix. Genes were classified as significantly differentially expressed at average count > 5, multiple testing adjusted p-value < 0.05, and -0.585 < log2FC > 0.585. The Ensemble annotation was enriched with UniProt data.”
Bias	9	Describe any efforts to address potential sources of bias<br>All tissue specimens were processed in a standardized manner by experienced staffs, which were blinded to clinical data.<br>“All tissue specimens were processed by blinded staffs.”
Study size	10	Explain how the study size was arrived at<br>We performed a single center all-comers study. All patients were treated according to SOP and GCP by the same team. All patients undergoing PEA at the Kerckhoff Heart and Thorax Center between 2016 and 2020 that gave informed consent were included. Samples were used to evaluate data derived from animal studies in human tissue samples.
Quantitative variables	11	Explain how quantitative variables were handled in the analyses. If applicable, describe which groupings were chosen and why<br>Disease severity of the patients was scored based on cardiac index, TAPSE / systolic pulmonary arterial pressure und N-terminal pro-brain natriuretic peptide in line with European Society of Cardiology (ESC) guide lines. Patients were stratified into low, intermediate and high one-year mortality risk groups and ranked for disease severity by equally weighting these parameters, so that higher ranks correlated with lower mortality.<br>Patients were characterized by the clinical staff by the score as described above. We did not perform additional analyses of clinical data or any clinical intervention related to our study. Score levels were used to group the patients.<br>Results: ”Disease severity of the patients at BL was scored based on cardiac index, TAPSE / systolic pulmonary arterial pressure (sPAP) und N-terminal pro-brain natriuretic peptide (NT-proBNP) in line with European Society of Cardiology (ESC) guide lines. Patients were stratified into low, intermediate and high one-year mortality risk groups and ranked for disease severity by equally weighting these parameters, so that higher ranks correlated with lower mortality.”
Statistical methods	12	(*a*) Describe all statistical methods, including those used to control for confounding<br>Statistical methods are described within the figure legends (presentation of data from human samples in Figure 7 of the manuscript):<br>Fig. 7D: Strategy to define expressed genes (IDs) at prePEA state that correlate significantly with ESC rank (Pearson r > or < 0.3 and a p value ≤ 0.01). This set of

		1925 IDs was intersected with the 224 PAB-regulated genes from the rat model, resulting in a significant (Fisher's exact test, $p < 0.0001$) overlap of 55 genes.<br>Fig. 7F: Correlation of mRNA expression (normalized (norm.) read counts)) with ESC rank at prePEA state for prototypical genes. The graphs display values for 71 patients, linear regression lines (in red), 95% confidence intervals (in gray), Pearson r and p values (*p ≤ 0.05, **p ≤ 0.01, ***p ≤ 0.001, ****p ≤ 0.0001).<br>Fig. 7G: mRNA expression of genes at time of surgery (prePEA, n=71) and follow up (postPEA, n=24). Red colors mark values from patients with lowest mortality risk. Black lines show means and asterisks indicate significant changes (Mann-Whitney test, *p ≤ 0.05, **p ≤ 0.01, ***p ≤ 0.001, ****p ≤ 0.0001).<br>No control for confounding was performed.
		(*b*) Describe any methods used to examine subgroups and interactions<br>No interactions were analyzed.
		(*c*) Explain how missing data were addressed<br>The clinical risk score was assessed for the grouping of all prePEA patients (100%). No further data were analyzed.
		(*d*) If applicable, explain how loss to follow-up was addressed<br>Separate presentation of all 24 patients with follow-up (presented in Fig. 7G+H)
		(*e*) Describe any sensitivity analyses<br>No sensitivity analysis was performed.
**Results**		
Participants	13*	(a) Report numbers of individuals at each stage of study—eg numbers potentially eligible, examined for eligibility, confirmed eligible, included in the study, completing follow-up, and analysed<br>"To explore the conservation and relevance of the PAB-regulated gene sets for humans, we analyzed 95 RNAseq data sets from CTEPH patients. RV biopsies were obtained during thoracic surgery at base line (BL, prePEA) and for 24 patients from the septum (by right heart catheter) during follow up, 12 months after pulmonary endarterectomy (FU, postPEA) (Fig. 7A)."
		(b) Give reasons for non-participation at each stage<br>Septum biopsies were taken from all patients that underwent standardized 12 months follow up (24 patients). 47 patients did not undergo follow up biopsies due to withdrawn consent.
		(c) Consider use of a flow diagram<br>Not applicable.
Descriptive data	14*	(a) Give characteristics of study participants (eg demographic, clinical, social) and information on exposures and potential confounders<br>Additional characteristics are presented in the accompanying manuscript by Jafari et al. (NCVR-2023-07-1957).
		(b) Indicate number of participants with missing data for each variable of interest<br>RNA-seq data sets:<br>baseline investigation, prePEA: 71 patients<br>follow-up investigation, postPEA: 24 patients<br>only baseline investigation, prePEA: 47 patients<br>only follow-up investigation, postPEA: 2 patients
		(c) Summarise follow-up time (eg, average and total amount)<br>follow up, 12 months after pulmonary endarterectomy
Outcome data	15*	Report numbers of outcome events or summary measures over time

		Additional measures are presented in the accompanying manuscript by Jafari et al. (NCVR-2023-07-1957).
Main results	16	(*a*) Give unadjusted estimates and, if applicable, confounder-adjusted estimates and their precision (eg, 95% confidence interval). Make clear which confounders were adjusted for and why they were included<br>No confounders were adjusted.
		(*b*) Report category boundaries when continuous variables were categorized<br>Not applicable: no continuous variables were categorized
		(*c*) If relevant, consider translating estimates of relative risk into absolute risk for a meaningful time period<br>Not applicable: We did not perform risk analyses with regard to outcome. Our data represent the analyses of gene expression changes (RNA-Seq).
Other analyses	17	Report other analyses done—eg analyses of subgroups and interactions, and sensitivity analyses<br>Our data show correlation of the expression of genes in relation to the ESC rank prePEA for (i) all patients, (ii) males and (iii) females.
**Discussion**		
Key results	18	Summarise key results with reference to study objectives<br>"To explore the conservation and relevance of the PAB-regulated gene sets for humans, we analyzed 95 RNAseq data sets from CTEPH patients. RV biopsies were obtained during thoracic surgery at base line (BL, prePEA) and for 24 patients from the septum (by right heart catheter) during follow up, 12 months after pulmonary endarterectomy (FU, postPEA) (Fig. 7A). Disease severity of the patients was scored based on cardiac index, TAPSE / systolic pulmonary arterial pressure (sPAP) und N-terminal pro-brain natriuretic peptide (NT-proBNP) in line with European Society of Cardiology (ESC) guide lines. Patients were stratified into low, intermediate and high one-year mortality risk groups (Fig. 7B) and ranked for disease severity by equally weighting these parameters, so that higher ranks correlated with lower mortality (Fig. 7C).<br>Of 16354 genes that were expressed at BL, 1925 correlated either negatively or positively with ESC rank (Fig. 7D). In this data set, 55 genes overlapped with 224 PAB-regulated genes (Fig. 7D-E, Source Data Fig. 7).<br>Most genes showed negative correlation, such as COL8A1, NPPB, PENK, ETV1, FSTL3 and AEBP1, indicating that their levels increased with more severe RHF (Fig. 7E-F). Only 7 genes, such as EPN3, showed a positive correlation, indicating that their expression might be beneficial (Fig. 7E-F). CILP, MAOA and NCAM1 mRNA changes prePEA were also significantly correlated with ESC rank but were below the stringent filtering criteria applied to the top 55 genes (Fig. 7E). Nine of these factors were confirmed to be regulated at the protein level in rats as shown in Fig. 5.<br>There was a significant reduction of mean COL8A1, NPPB, PENK and FSTL3 levels at FU (Fig. 7G), whereby data from patients at low risk were mostly below the average already at BL (see red dots in Fig. 7G). This was not the case for ETV1, EPN3, AEBP1 (Fig. 7G). Similarly, gene expression of 22 available paired samples showed a mixed pattern (Fig 7H), in line with the interpretation that averaged mRNA expression data obtained from two different regions of the heart (RV wall, septum) in a clinically heterogeneous cohort of patients are more variable and have limited sensitivity to reflect the course of disease."

Limitations	19	Discuss limitations of the study, taking into account sources of potential bias or imprecision. Discuss both direction and magnitude of any potential bias<br>"…the gene sets associated with CTEPH RVF may not be representative of all conditions resulting in pulmonary hypertension and RVF"
Interpretation	20	Give a cautious overall interpretation of results considering objectives, limitations, multiplicity of analyses, results from similar studies, and other relevant evidence<br>Discussion: comparison of data from the animal model and the patients.<br>Limitations: "Gene sets associated with CTEPH RVF may not be representative of all conditions resulting in pulmonary hypertension and RVF."
Generalisability	21	Discuss the generalisability (external validity) of the study results<br>"Gene sets associated with CTEPH RVF may not be representative of all conditions resulting in pulmonary hypertension and RVF."
**Other information**		
Funding	22	Give the source of funding and the role of the funders for the present study and, if applicable, for the original study on which the present article is based<br>The study work was supported by grants from the Deutsche Forschungsgemeinschaft (DFG, German Research Foundation). No conflict of interest was declared by any of the authors. No funding from pharmaceutical companies was provided in connection with this cohort study.

*Give information separately for exposed and unexposed groups.

Note: An Explanation and Elaboration article discusses each checklist item and gives methodological background and published examples of transparent reporting. The STROBE checklist is best used in conjunction with this article (freely available on the Web sites of PLoS Medicine at http://www.plosmedicine.org/, Annals of Internal Medicine at http://www.annals.org/, and Epidemiology at http://www.epidem.com/). Information on the STROBE Initiative is available at http://www.strobe-statement.org.
