# Supplementary material for: A common gene signature of the right ventricle in failing rat and human hearts
Source: Nat Cardiovasc Res. 2024 Jul 5;3(7):819–40. doi: 10.1038/s44161-024-00485-1 (PMC11358011; doi:10.1038/s44161-024-00485-1)
Supplement: Supplementary file 2 — Reporting Summary [file 44161_2024_485_MOESM2_ESM.pdf]

nature portfolio

Corresponding author(s): Susanne Rohrbach, Michael Kracht

Last updated by author(s): 07.06.2024

Reporting Summary

Nature Portfolio wishes to improve the reproducibility of the work that we publish. This form provides structure for consistency and transparency in reporting. For further information on Nature Portfolio policies, see our Editorial Policies and the Editorial Policy Checklist.

Statistics

For all statistical analyses, confirm that the following items are present in the figure legend, table legend, main text, or Methods section.

n/a	Confirmed	
☐	☒	The exact sample size (*n*) for each experimental group/condition, given as a discrete number and unit of measurement
☐	☒	A statement on whether measurements were taken from distinct samples or whether the same sample was measured repeatedly
☐	☒	The statistical test(s) used AND whether they are one- or two-sided<br>*Only common tests should be described solely by name; describe more complex techniques in the Methods section.*
☒	☐	A description of all covariates tested
☐	☒	A description of any assumptions or corrections, such as tests of normality and adjustment for multiple comparisons
☐	☒	A full description of the statistical parameters including central tendency (e.g. means) or other basic estimates (e.g. regression coefficient) AND variation (e.g. standard deviation) or associated estimates of uncertainty (e.g. confidence intervals)
☐	☒	For null hypothesis testing, the test statistic (e.g. *F*, *t*, *r*) with confidence intervals, effect sizes, degrees of freedom and *P* value noted<br>*Give P values as exact values whenever suitable.*
☒	☐	For Bayesian analysis, information on the choice of priors and Markov chain Monte Carlo settings
☒	☐	For hierarchical and complex designs, identification of the appropriate level for tests and full reporting of outcomes
☐	☒	Estimates of effect sizes (e.g. Cohen's *d*, Pearson's *r*), indicating how they were calculated

Our web collection on statistics for biologists contains articles on many of the points above.

Software and code

Policy information about availability of computer code

Data collection

For rat cardiomyocyte (CM) samples, ribosomal RNA depletion of total RNA isolated from cardiomyocytes, library preparation and deep sequencing was performed by Novogene on the Illumina Novaseq platform (150 bp paired end set up).

For rat whole heart samples, polyadenylated RNA was purified from 500 ng of total RNA using the NEBNext® Poly(A) mRNA magnetic isolation module kit in 34 µl reaction volumes with adapted amounts of beads and buffers. RNAseq libraries were prepared using NEBNext® Ultra™ II directional RNA library prep kit for Illumina® and the manufacturer's protocol was adapted to reduce reaction volumes to 1 / 3 (7.7 µl mastermix added to RNA loaded beads). Libraries were quality controlled using Agilent Bioanalyzer high sensitivity DNA chips and DNA concentrations were determined using Qbit Analyzer with Qbit high sensitivity DNA reagent (Thermo Fisher scientific). Pooled libraries were prepared using SPRIselect beads (Beckman Coulter) and sequenced on an Illumina NextSeq 500 platform (75 bp single end set up).

For human heart samples, isolation of total RNA from both types of heart tissues, comprising 95 samples in total, was performed with Qiagen miRNeasy micro Kit and Covaris Cryo-Prep homogenization. All tissue specimens were processed by blinded staffs. 100 ng to 1 µg of total RNA was used for Hi-Mammalian whole transcriptome preparation (Takara Bio) and sequencing was performed on a Nextseq2000 instrument (72 bp single end set-up).

For rat smRNA-FISH, isolated ventricles were washed with ice-cold phosphate buffered saline and frozen in liquid nitrogen. 7 µm tissue slices from adverse sections obtained from the two chamber ventricle level (without valves) were analysed by smRNA FISH using RNAscope fluorescent multiplex assay (Advanced Cell Diagnostics, Bio-techne.com) according to the manufacturer's protocol for fresh frozen tissue. Briefly, tissue was permeabilized by dehydration and protease treatment, followed by hybridization of the selected, commercial RNA probes for Nppa, Nppb, Penk, Acta1, Ankrd23 and Tceal7 and amplification of the signals. Negative control probes for each channel (C1, C2) were directed against a bacterial RNA and provided by ACD Bio-techne.com.

For proteomic analyses of rat hearts, rat LV and RV heart tissue samples were placed in Lysing Matrix D tubes containing 4 M GuHCl buffer (4 M GuHCl, 25 mM EDTA, 50 mM sodium acetate, pH=5.8, with protease / phosphatase inhibitors) and homogenized using Lysing Matrix D in a FastPrep24 tissue homogenizer at 6500 rpm for 15 seconds, twice. The supernatant was sonicated twice for 10 seconds, prior to centrifugation for 20 minutes at 16,000 g at 4°C. Protein concentration was measured with Pierce BCA Protein Assay Kit (Thermo Fisher Scientific). For each sample, 50 µg of protein was precipitated using 10x volume of absolute ethanol and incubated at -20°C overnight. The samples were centrifuged for 30 minutes at 16,000 g at 4°C, and the pellets was dried using a SpeedVac (Thermo Fisher Scientific). Protein pellets were dissolved and denatured by 6 M urea / 2 M thiourea, reduced by 10 mM dithiothreitol for 1 h at 37°C, and alkylated using 50 mM iodoacetamide for 1 h at room temperature in the dark. The samples were precipitated by using 1 ml of pre-chilled acetone and incubated at -20°C overnight. The samples were centrifuged for 30 minutes at 16,000 g at 4°C, and the supernatant was discarded. The protein pellet was dried and resuspended in 0.1 M triethylammonium bicarbonate (TEAB, pH 8.5). Proteins were digested by Trypsin / LysC (Promega, enzyme: protein ratio = 1:25) overnight at 37°C with shaking. The digestion was stopped by adding 1% trifluoroacetic acid (TFA) and peptides were cleaned using C18 cartridges on a Bravo AssayMAP robot (Agilent). After SpeedVac, the dried peptides were resuspended in 0.1 M TEAB to obtain a concentration of 1 µg / µl. A pooled sample was made by mixing the same amount of each sample. 20 µg of samples along with pooled samples were labelled with TMTpro 18plex Reagents (Thermo Fisher Scientific) according to the manufacturer's instructions. After quenching with 5% hydroxylamine, 1 pooled sample and 16 samples labelled with different TMTpro tags were mixed together, dried and resuspended in 0.1% triethylamine. Peptide fractionation was performed using high pH Reversed-Phase HPLC on a ZORBAX Extend 300 C18 column (Agilent, 4.6 x 150 mm) and 16 fractions were collected for 300 µg of each mixed TMT-labelled sample. The fractionated samples were dried using SpeedVac and resuspended in 37.5 µl LC solution (2% acetonitrile and 0.05% trifluoroacetic acid in LC-MS grade H2O). For LC-MS/MS analysis, 5 µl of each fractionated sample was injected and separated by reversed-phase nano-flow HPLC (Thermo Fisher Scientific, Ultimate 3000 RSLCnano) on a 50 cm EASY-SPRAY C18 column (Thermo Fisher Scientific) over 2 h gradient at a flow rate of 0.25 µl / minute as follows: 0-1 minutes, 1% B; 1-6 minutes, 1%-6% B; 6-40 minutes, 6%-18% B; 40-70 minutes, 18%-35% B; 70-80 minutes, 35%-45% B; 80-81 minutes, 45-99% B; 81-89.8 minutes, 99% B; 90-120 minutes, 1% B; where A = 0.1% formic acid in LC-MS grade H2O, B = 80% acetonitrile and 0.1% formic acid in LC-MS grade H2O. The separated peptides were directly injected into an Orbitrap Fusion Lumos Tribrid Mass Spectrometer (Thermo Fisher Scientific) interfaced with FAIMS Pro Duo in front. In the FAIMS, 3 compensation voltages (CV = -40V, -55V, and -70V) were used alternatively to further separate the peptides in gas phase. The peptides were analyzed using a data-dependent MS2 method with Full MS scan range 400-1600 m/z, resolution 120,000, in Orbitrap, and data-dependent MS2 scans were performed on the most abundant precursors from Full MS scan using higher-energy collisional dissociation (HCD) and detected in Orbitrap with resolution 50,000 and isolation windows 0.7 m/z. The TMTpro reporter ions were also generated during the HCD fragmentation and recorded in the same MS2 spectra. Cycle time was set at 4.5 seconds (1.5 seconds for each CV) and dynamic exclusion was enabled. The data analysis was performed using Proteome Discoverer version 2.4.1.15 (Thermo Fisher Scientific) with Mascot 2.6.0 (Matrix Sciences). The following parameters were used: a combined RAT reference proteome database complemented with MOUSE and HUMAN UniProt/SwissProt database protein entries (release 2022_01, 21589+17107+20376 protein entries) was used because the rat SwissProt database is not as complete as mouse and human; trypsin was used as enzyme and 2 missed cleavages were allowed; precursor mass tolerance was set at 10 ppm and fragment mass tolerance was 20 mmu; TMTpro 18plex tag on lysine and peptide N-terminal, carbamidomethylation on cysteine were set as static modifications; oxidation on methionine, lysine, and proline was set as variable modifications. Afterwards, identified protein lists were manually checked and for proteins identified in different species, rat proteins were kept as first choice, followed by mouse proteins and human proteins. In total, 4149 protein IDs were identified. TMT reporter ion signal was used as peptide quantitative value and summed up to represent the protein abundances. The data were normalized using the total peptide abundance then further scaled using the pooled sample abundance as control, correcting for technical variation between injections and TMT groups. The scaled abundance values were exported into Excel and used for further analysis.

Data analysis

For rat cardiomyocyte RNA samples, raw data were processed using Nextflow and the nf-core / RNAseq pipeline (vs. 3.2). Sequence reads were trimmed by Trimgalore (v0.6.6), mapped to the rat reference genome Rnor6.0 with UCSC feature annotations using STAR (v2.6.1d) and read counts were extracted with FeatureCounts (Rsubreads v2.2.6), disabling multiple overlaps. Over 90% of all reads present in the 82 samples were mapped to genomic features of the rat genome and > 80% were uniquely mapped and represented by at least 25 million reads per sample. Differential expression was analysed by DESeq2 (v1.28.1) using the independent filtering option and beta_prior LFC-shrinkage, and the Galaxy instance of the Justus Liebig University Giessen.

For rat whole heart RNA samples, raw data were processed using the Nextflow and nf-core / RNAseq pipeline, version 1.5. Sequence reads were trimmed by Trimgalore (v0.6.4), mapped to the rat reference genome Rnor6.0 with UCSC feature annotations using STAR (v2.6.1d) and read counts were extracted with FeatureCounts (Rsubreads v2.2.6), disabling multiple overlaps. For all 99 samples, over 95% of reads were mapped to Rnor6.0 features, > 75% were uniquely mapped and represented by at least 27 million per sample. Differential expression was analysed by DESeq2 (v1.28.1) as described above.

For human heart RNA samples, Trimmomatic (v. 0.39) was employed to trim reads after a quality drop below a mean of Q15 in a window of 5 nucleotides and keeping only filtered reads longer than 15 nucleotides. Reads were aligned versus Ensembl human genome version hg38 (Ensembl release 104) with STAR 2.7.10a. Aligned reads were filtered to remove duplicates with Picard 2.27.1 (Picard Toolkit. 2019. Broad Institute, GitHub Repository. https://broadinstitute.github.io/picard/; Broad Institute; RRID:SCR_006525), multi-mapping events and ribosomal or mitochondrial reads. Gene counts were established with featureCounts 2.0.2 by aggregating reads overlapping exons on the correct strand excluding those overlapping multiple genes. The raw count matrix was normalized with DESeq2 version 1.30.1. Contrasts were created with DESeq2 based on the raw count matrix. Genes were classified as significantly differentially expressed at average count > 5, multiple testing adjusted p-value < 0.05, and -0.585 < log2FC > 0.585. The Ensemble annotation was enriched with UniProt data (Activities at the Universal Protein Resource (UniProt)).

Z scores were based on the formula $z = (x - \mu) / \sigma$, where x is the gene expression value, µ is the average mRNA expression per group and σ is the standard deviation. All graphs and statistical tests of non-omics data (two-sided t-tests and ANOVA, correlation analyses) were performed using GraphPad Prism, v 9.4.1 (GraphPad Software, LLC) or Excel2016. Venn diagrams were generated online using a Venn diagram tool (http://bioinformatics.psb.ugent.be/webtools/Venn/). For heatmap illustrations, z-score calculations or cluster analyses, Excel 2016 or the web tool Morpheus (https://software.broadinstitute.org/morpheus) were used. Gene sets were segregated by k-means clustering with "one minus pearson correlation" based on row values. Protein-protein interaction network analyses were based on the newest version of the STRING database (https://string-db.org/) and visualized using Cytoscape 3.9.1 using the integrated STRING functionalities and all ontology data bases of the STRING application.

Pathway enrichment analyses were performed online by Metascape (www.metascape.org) with the following default settings: for individual or multiple gene lists, KEGG Pathway, GO Biological Processes, Reactome Gene Sets, Canonical Pathways, CORUM, WikiPathways, and

PANTHER pathways were used as ontology sources. All genes in the genome were used as the enrichment background. Terms with a p-value < 0.01, a minimum count of 3, and an enrichment factors (ratio between observed counts and counts expected by chance) > 1.5 were collected and clustered by their membership similarities. P values were calculated based on the cumulative hypergeometric distribution, and q values were calculated using the Benjamini-Hochberg procedure to account for multiple testings. Kappa scores were used as the similarity metric for hierarchical clustering of enriched terms, and sub-trees with a similarity of > 0.3 were considered a cluster. The most statistically significant term within a cluster were chosen to represent the cluster 71. In case of multiple input gene lists, these were merged into one list called "_FINAL". If terms were enriched in several individual gene lists and / or in the _FINAL gene list, the best p value was chosen as the final p value.

Enrichment analyses for transcription factor-regulated gene sets were performed online using using the network transcription factor (TF) target functionality of WebGestalt (www.webgestalt.org) and the transcription factor gene sets (TF) of the Molecular Signatures Database (MSigDB) (https://www.gsea-msigdb.org/gsea/msigdb) with the following settings. Enrichment method: ORA, organism: rnorvegicus, enrichment categories: network_Transcription_Factor_target, ID type: genesymbol, reference list: all mapped entrezgene IDs from the selected platform genome. Parameters for the enrichment analysis: minimum number of IDs in the category: 5, maximum number of IDs in the category: 2000, FDR method: Benjamini-Hochberg, significance level: Top 10.

Secretome annotations were based on the published list of 6,943 high-confidence human secreted proteins which were generated from 330,427 human proteins derived from databases of UniProt, Ensembl, AceView, and RefSeq (SPRomeDB; www.unimd.org/SPRomeDB). 6,267 of 6,943 (90.3%) of these proteins have the supporting evidences from a large amount of mass spectrometry (MS) and RNA-seq data.

For analysis of RNA-FISH signals, negative control probes for each channel (C1, C2) were directed against a bacterial RNA and provided by ACD Bio-techne.com. Microscopy analysis was performed using a Leica THUNDER imager (Leica Microsystems CMS GmbH) and Leica Application suite X (Version 3.7.4.23463), using pre-defined exposure times (Phase contrast: 55ms, DAPI: 80ms, C1 (555nm): 100ms, C2 (635nm): 700ms). After bright field preview scanning, preserved morphologies of whole heart sections and of fluorescence signals of single tiles were validated. Tile selections for region specific quantification were based on bright field scanning. Spots and nuclei (200 x magnification) were detected and quantified by Icy software (v.2.4.2.0) (https://icy.bioimageanalysis.org) using the following settings: For nuclei, the thresholder value was adjusted to 15 k-means classes and HK-means to intensity classes of 12 and a minimum object size of 20 px. For spot detection, the object size was defined as 1 px with a sensitivity of 30. Spot numbers of each image were divided by the numbers of detected nuclei and normalized using a background factor derived from dividing mean background level by the background of the individual experiment. Imaging of all tiles across the entire section resulted in whole section overview images using Leica Application Suite X.

For proteomic analyses, scaled, normalized LC-MS / MS data derived from TMT labeling were Log2 transformed and width normalized using Perseus software, version 1.6.15.0. The eight biological replicates were assigned to one analysis group per condition. The data matrix was reduced to 3768 IDs based on 75% (i.e. 6 out of 8) valid values in at least one group. For separation of sex-specific proteomes, condition groups were split according to sex, resulting in 4039 IDs with 75% valid values. Significantly differentially expressed proteins (DEPs) between groups were identified based on t-tests and a –log10 p value ≥ 1.3 using Perseus functionalities. Subsequent filtering steps and heatmap representations were performed in Excel 2016 according to the filtering criteria and thresholds described in the figure legends.

For processing of single nucleus RNAseq data from left ventricles of hearts from donors and patients with dilated or hypertrophic cardiomyopathy, data were downloaded from the Single Cell Portal of the Broad Institute (https://singlecell.broadinstitute.org/single_cell/study/SCP1303/). In total, processed information for 592,689 cells was obtained in h5ad-format (downloaded file: human_dcm_hcm_scportal_03.17.2022.h5ad). The file was converted into h5seurat-format and subsequently loaded into the R environment (https://www.R-project.org/) using Seurat version 5.0.0. The same procedures were followed to re-use sc/sn RNAseq data from the human heart atlas, version 2, comprising 704,296 individual cells representing 12 cardiac cell types (https://www.heartcellatlas.org/index.html).

Differential gene expression between disease conditions and control donors was calculated in cardiomyocytes (I, II, III or the union of all subtypes) using a pseudo-bulk approach as outlined in (https://hbctraining.github.io/scRNA-seq_online/lessons/06a_integration_harmony.html). Data were converted into a single-cell experiment object and subsets of cardiomyocyte compartments were selected (based on cell_type_leiden0.6 column). Data were split according to samples and cell types and subsequently DESeq2 (v.1.42.0) was used to identify differentially expressed genes for contrasts HCM vs NF and DCM vs NF. Subsequently, genes with a base mean of less than 5 reads were removed and gene set enrichment analyses (GSEA) were performed across 5 different gene signatures: RHF core signature (113), hLHF+Rat PAB (57), hLHF+hCTEPH+Rat PAB (35), hCTEPH+ Rat PAB (21) and Rat PAB (111). GSEA was performed using the GSEA function of the clusterProfiler package based on the Wald test statistic derived from the DESeq2 analysis. Furthermore, the h5ad file was loaded into a cellxgene instance extended with the functionality of the VIP plug-in, which was used to generate Fig. 8C, Fig. 8F, Extended Data Figs. 3D, 4B, 8 and 9).

The R code for the pseudo-bulk analysis of differential gene expression in Cardiomyocytes from published sc / sn RNA-seq data and subsequent GSEA analysis of gene signatures is accessible at (https://doi.org/10.5281/zenodo.10973971).

For manuscripts utilizing custom algorithms or software that are central to the research but not yet described in published literature, software must be made available to editors and reviewers. We strongly encourage code deposition in a community repository (e.g. GitHub). See the Nature Portfolio guidelines for submitting code & software for further information.

Data

Policy information about availability of data

All manuscripts must include a data availability statement. This statement should provide the following information, where applicable:

- Accession codes, unique identifiers, or web links for publicly available datasets
- A description of any restrictions on data availability
- For clinical datasets or third party data, please ensure that the statement adheres to our policy

For cardiomyocyte (CM) RNAseq, CM were isolated from RVs and LVs of 41 animals resulting in 82 RNAseq data sets (see Fig. 1A). For whole heart RNAseq, RV, septum and LV tissues were separated from hearts of 33 animals resulting in 99 RNAseq data sets in total (see Extended Data Fig. 7A).

The rat cardiomyocyte and whole heart RNAseq data sets of this study have been submitted to GEO: GSE216263 (https://www.ncbi.nlm.nih.gov/geo/query/acc.cgi?

acc=GSE216263) and GSE216264 (https://www.ncbi.nlm.nih.gov/geo/query/acc.cgi?acc=GSE216264).

For RNAseq of human CTEPH patient heart tissues, biopsies of the free RV wall from 71 patients were collected at base line (BL, prePEA) during PEA. In 24 patients, RV myocardial biopsies were obtained during right heart catheterization (RHC) 12 months after PEA (follow-up, FU, prePEA). The human CTEPH RNAseq data have been submitted to GEO: GSE249697 (https://www.ncbi.nlm.nih.gov/geo/query/acc.cgi?acc=GSE249697).

For proteomic analyses of rat hearts, right and left ventricle were separated from 56 animals (n = 8 per group), resulting in 112 samples for proteomic analysis in total. The mass spectrometry proteomics data have been deposited to the ProteomeXchange Consortium via the PRIDE partner repository with the dataset identifier PXD047022 (https://proteomecentral.proteomexchange.org/cgi/GetDataset?ID=PXD047022).

For processing of published single cell nucleus transcriptomic data, single nucleus RNAseq data from 592,689 cells of left ventricles from hearts from donors and patients with dilated or hypertrophic cardiomyopathy were downloaded from the Single Cell Portal of the Broad Institute (https://singlecell.broadinstitute.org/single_cell/study/SCP1303/).
For processing of sc/sn RNAseq data from the human heart atlas, version 2, comprising 704,296 individual cells representing 12 cardiac cell types, data were downloaded from the Heart Cell Atlas (https://www.heartcellatlas.org/index.html).
The following publically accessible data bases were used: STRING database (https://string-db.org/); Metascape (https://www.metascape.org) ; WebGestalt (www.webgestalt.org); Molecular Signatures Database (MSigDB) (https://www.gsea-msigdb.org/gsea/msigdb); SPRomeDB (https://www.unimd.org/SPRomeDB).
The remaining data generated in this study are provided in the Supplementary Information / Source Data sections. Source data are provided with this paper.

Research involving human participants, their data, or biological material

Policy information about studies with human participants or human data. See also policy information about sex, gender (identity/presentation), and sexual orientation and race, ethnicity and racism.

Reporting on sex and gender: In this study, all RNAseq and proteomic data were additionally analyzed according to sex.

Reporting on race, ethnicity, or other socially relevant groupings: Patients were not grouped according to race, ethnicity or other social criteria. 71 Patients prePEA were grouped according to 1 year mortality risk by applying a modified version of the European Society of Cardiology (ESC) guidelines risk stratification model (Humbert et al., 2023), using the following criteria. Low risk (< 5%): cardiac index (CI) ≥ 2.0 L / min / m2, NT-proBNP < 300 ng / L, tricuspid annular plane systolic excursion (TAPSE) / systolic pulmonary arterial pressure (sPAP) > 0.32 mm / mmHg, intermediate risk (5-20%): NT-proBNP: 300-1100 ng / L, TAPSE / sPAP: 19 – 32 mm / mmHg and high risk (> 20%): CI < 2.0 L / min/m2, NT-proBNP > 1100 ng / L, TAPSE / sPAP < 0.19 mm/mmHg. Additionally, in the low or high groups 1 parameter was allowed to differ, but 2 out of 3 had to meet the pre-defined values. Otherwise, patients were assigned to the intermediate group.

Population characteristics: Data on demographics, symptoms and comorbidities were collected for all patients. Baseline characteristics concerning LV and RV dimensions, function and pressure gradients were further evaluated by transthoracic echocardiography and right heart catheterisation. The 71 patients analyzed by RNAseq at the prePEA stage included 49 male and 22 female patients with a mean age of 58.8 years (range 20.1-80.2 years).

Recruitment: The present prospective study comprised a total number of 73 patients (including two without prePEA RNAseq data sets) with a final diagnosis of chronic thromboembolic pulmonary hypertension (CTEPH), who were treated by pulmonary endarterectomy (PEA) at the Kerckhoff Heart and Thorax Center between 2016 and 2020. Biopsies of the free right ventricular wall from 71 patients were collected at base line (BL, prePEA) during PEA. In 24 patients, RV myocardial biopsies were obtained during right heart catheterization (RHC) 12 months postPEA at follow-up (FU). In this case, to account for technical and safety aspects, the specimens were taken from the interventricular septum. RNA samples prePEA and postPEA were available for 22 patients, while only postPEA samples were available from two additional patients.

Ethics oversight: All patients provided written informed consent for their participation in the study and approval of the institutional review board of the Justus Liebig University of Giessen (AZ 44 / 14, 144 / 11, 145 / 11, 146 / 11, 199 / 15) was obtained. The investigation conforms to the principles outlined in the Declaration of Helsinki.

Note that full information on the approval of the study protocol must also be provided in the manuscript.

Field-specific reporting

Please select the one below that is the best fit for your research. If you are not sure, read the appropriate sections before making your selection.

☒ Life sciences ☐ Behavioural & social sciences ☐ Ecological, evolutionary & environmental sciences

For a reference copy of the document with all sections, see nature.com/documents/nr-reporting-summary-flat.pdf

Life sciences study design

All studies must disclose on these points even when the disclosure is negative.

Sample size: No sample size calculation was performed using statistical methods.
For rat cardiomyocytes, more than five animals were included in each group according to recent guide lines (https://doi.org/10.1111/bph.15868). For rat whole heart samples, some groups were smaller due to limited availability of animals.

	The samples sizes of human biopsies used for RNA-seq analysis were based on the availability of tissue samples obtained during pulmonary endarterectomy and at follow-up during right heart catheterization. Sample sizes for microscopy analyses are indicated in each figure. Samples sizes for functional in vitro analyses of adult rat cardiomyocytes are indicated in each figure.
Data exclusions	No data were excluded.
Replication	All major findings reported in this study for rat animal studies were reproduced in independent biological experiments. The number of biologically independent replicates and individual data points are indicated in the figure legends. Patient-derived data represent Independent replicates.
Randomization	Rats from the same strain were randomly assigned to study groups. No randomization was performed for patient-derived data sets and patients were grouped according to their clinical characteristics.
Blinding	All RNA-sequencing and LC-MS / MS runs were performed blinded. No blinding was performed for data analysis. RNA-FISH hybridization and data analyses were performed unblinded by two independent researchers who assessed the predefined experimental groups using automated software-based quantification of microscopic raw data.

Reporting for specific materials, systems and methods

We require information from authors about some types of materials, experimental systems and methods used in many studies. Here, indicate whether each material, system or method listed is relevant to your study. If you are not sure if a list item applies to your research, read the appropriate section before selecting a response.

Materials & experimental systems

n/a	Involved in the study
☐	☒ Antibodies
☒	☐ Eukaryotic cell lines
☒	☐ Palaeontology and archaeology
☐	☒ Animals and other organisms
☐	☒ Clinical data
☒	☐ Dual use research of concern
☒	☐ Plants

Methods

n/a	Involved in the study
☒	☐ ChIP-seq
☒	☐ Flow cytometry
☒	☐ MRI-based neuroimaging

Antibodies

Antibodies used	The following antibodies were used: Rabbit polyclonal anti Penk antibody (Aviva Systems Biology, Cat. # OAAN01715, Lot #20400101, 1:1000 in TBS-T, 2.5% milk); Rabbit polyclonal anti alpha-Tubulin antibody (Cell Signaling Technology, Cat. # 2144, Lot # 7, 1:1000 in TBS-T, 2.5% milk); Mouse monoclonal anti troponin T antibody (ThermoFisher Cat. # MA5-12960, Lot # 2676914, 1:200 in 2% BSA, 0.2% Triton-X-100 in PBS); Mouse monoclonal anti vimentin antibody (Santa Cruz Biotechnology Cat. # sc-6260, Lot # C2523, 1:100 in 2% BSA, 0.2% Triton-X-100 in PBS); Horse polyclonal anti-mouse HRP-linked secondary antibody (Cell Signaling Technology, Cat. # 7076, Lot # 0, 1:10,000 in TBS-T, 2.5% milk); Goat polyclonal anti-rabbit, HRP-linked secondary antibody (Cell Signaling Technology, Cat. # 7074, Lot # 30, 1:10,000 in TBS-T, 2.5% milk); Donkey polyclonal anti-mouse Alexa Fluor® 488 secondary antibody (ThermoFisher Cat. # A-21202, Lot # 1890861, 1:1000 in PBS), Donkey polyclonal anti-rabbit Alexa Fluor® 555 secondary antibody (ThermoFisher Cat. # A-31572, Lot # 1891766, 1:1000 in PBS).
Validation	All antibodies used in this study were validated by the correspondening commercial manufacturers and the information is accessible on their websites.<br>Anti penk antibody: validated for Western Blot and Immunohistochemistry; anti alpha-tubulin antibody: validated for Western Blot, Flow Cytometry, Immunocytochemistry and Immunohistochemistry¸ anti troponin T antibody: Western Blot, Flow Cytometry, Immunocytochemistry; anti vimentin antibody: validated for Western Blot, Flow Cytometry, Immunoprecipitation, Immunocytochemistry and Immunohistochemistry; anti mouse HRP-linked secondary antibody validated for Western Blot; anti rabbit HRP-linked secondary antibody validated for Western Blot; anti mouse Alexa Fluor® 488 secondary antibody: Immunocytochemistry and Immunohistochemistry; anti rabbit Alexa Fluor® 555 secondary antibody: Flow Cytometry, Immunocytochemistry and Immunohistochemistry.

Animals and other research organisms

Policy information about studies involving animals; ARRIVE guidelines recommended for reporting animal research, and Sex and Gender in Research

Laboratory animals	Ascending aortic banding (AOB) and pulmonary artery banding (PAB) were performed in 7 weeks old weanling hybrid rats (Wistar Kyoto x Lewis rats).
Wild animals	This study did not involve wild animals.

Reporting on sex

The sex of rats used for cardiomyocyte and whole heart isolation is indicated. The sex of patients is indicated.

Field-collected samples

This study did not involve field-collected samples.

Ethics oversight

Animal studies
The experimental protocols were approved by the regional authorities and ethics committees for animal research (RP Giessen, registered under the number G14-2017) and conformed to the guidelines from Directive 2010/63/EU of the European Parliament on the protection of animals and ARRIVE guidelines 2.0.

Note that full information on the approval of the study protocol must also be provided in the manuscript.

Clinical data

Policy information about clinical studies
All manuscripts should comply with the ICMJE guidelines for publication of clinical research and a completed CONSORT checklist must be included with all submissions.

Clinical trial registration

Not applicable as this was an observational study.

Study protocol

The present prospective study included a total number of 73 patients with a final diagnosis of chronic thromboembolic pulmonary hypertension (CTEPH), who were treated by pulmonary endarterectomy (PEA) at the Kerckhoff Heart and Thorax Center between 2016 and 2020. Biopsies of the free right ventricular wall from 71 patients were collected at base line (BL) during PEA. In 24 patients, RV myocardial biopsies were obtained during right heart catheterization (RHC) 12 months after PEA (follow-up, FU). In this case, to account for technical and safety aspects, the specimens were taken from the interventricular septum.

Data on demographics, symptoms and comorbidities were collected for all patients. Baseline characteristics concerning LV and RV dimensions, function and pressure gradients were further evaluated by transthoracic echocardiography and right heart catheterisation. In brief, clinical examination, echocardiography, 12-lead electrocardiogram, laboratory tests, 6-minuts walk test, ventilation-perfusion-scan, computed tomography angiography, right-left-heart catheterization, and pulmonary angiography were assessed for all patients at the baseline (BL). The final diagnosis of CTEPH was made according to the guidelines in symptomatic patients after three months of effective anticoagulation, with a mean pulmonary arterial pressure (mPAP)≥25 mmHg at rest and typical obstructive pulmonary vascular lesions on imaging diagnostics (Galiè et al., 2016).

All patients provided written informed consent for their participation in the study and approval of the institutional review board of the University of Giessen (AZ 44 / 14, 144 / 11, 145 / 11, 146 / 11, 199 / 15) was obtained. The investigation conforms to the principles outlined in the Declaration of Helsinki.

Data collection

For RNA isolation, biopsies of the free right ventricular wall from 71 patients were collected at base line (BL) during PEA. In 24 patients, RV myocardial biopsies were obtained during right heart catheterization (RHC) 12 months after PEA (follow-up, FU). In this case, to account for technical and safety aspects, the specimens were taken from the interventricular septum. All patients provided written informed consent for their participation in the study and approval of the institutional review board of the University of Giessen (AZ 44 / 14, 144 / 11, 145 / 11, 146 / 11, 199 / 15) was obtained. The investigation conforms to the principles outlined in the Declaration of Helsinki.

Outcomes

71 patients were grouped according to 1 year mortality risk by applying a modified version of the European Society of Cardiology (ESC) guidelines risk stratification model (Humbert et al., 2023), using the following criteria. Low risk (< 5%): cardiac index (CI) ≥ 2.0 L / min / m2, NT-proBNP < 300 ng / L, tricuspid annular plane systolic excursion (TAPSE) / systolic pulmonary arterial pressure (sPAP) > 0.32 mm / mmHg, intermediate risk (5-20%): NT-proBNP: 300-1100 ng / L, TAPSE / sPAP: 19 – 32 mm / mmHg and high risk (> 20%): CI < 2.0 L / min/m2, NT-proBNP > 1100 ng / L, TAPSE / sPAP < 0.19 mm/mmHg. Additionally, in the low or high groups 1 parameter was allowed to differ, but 2 out of 3 had to meet the pre-defined values. Otherwise, patients were assigned to the intermediate group.
